# The Network Model of Mentalization, Social Vulnerability, and the Self in Autism: A Comparison With Neurotypical Adults

**DOI:** 10.1002/aur.70263

**Published:** 2026-06-16

**Authors:** Szilárd Holka, Dániel Sörnyei, Ágota Vass, Levente Rónai, Kinga Farkas

**Affiliations:** ^1^ Department of Psychiatry and Psychotherapy Semmelweis University Budapest Hungary; ^2^ HUN‐REN Institute of Cognitive Neuroscience and Psychology Research Centre of Natural Sciences Budapest Hungary; ^3^ Department of Clinical Psychology Semmelweis University Budapest Hungary; ^4^ University Research and Innovation Center Physiological Controls Research Center; and John von Neumann Faculty of Informatics Óbuda University Budapest Hungary; ^5^ Institute of Psychology ELTE Eötvös Loránd University Budapest Hungary; ^6^ Institute of Psychology University of Szeged Szeged Hungary

**Keywords:** autism, mentalization, network analysis, psychosocial vulnerability, schizotypy, self‐perception

## Abstract

Autism spectrum disorder (ASD) is characterized by alterations in social understanding and self‐related experience that overlap with broader dimensions of psychosocial vulnerability. These domains are tightly interconnected, motivating the use of analytic approaches that can capture their organization as complex associations rather than as isolated dimensions. We applied network analysis to autistic adults (*N* = 156) and demographically matched neurotypical controls (*N* = 454). Gaussian graphical models were estimated using sum scores of psychosocial constructs, and networks were compared using centrality metrics and the network comparison test. Compared to neurotypical participants, autistic individuals showed higher levels of psychosocial difficulties and lower global network strength. Mentalization emerged as a central node in both groups, while autistic traits were more central in the neurotypical network and trait anxiety showed relatively higher centrality in the ASD network. These findings suggest that psychosocial vulnerability in autism is characterized by a distinct and less integrated network organization, with mentalization playing a central role across groups and anxiety showing a relatively greater centrality in ASD. Network‐based approaches may therefore help identify mechanism‐relevant targets for intervention and refine dimensional models of social and self‐related functioning in autism.

## Summary

Autism affects how people understand themselves and others, including social relationships, emotions, and everyday stress. These experiences are closely connected rather than independent. We studied how these different experiences relate to each other in autistic and non‐autistic adults using a method that looks at patterns of connections rather than single traits. In autism, these connections were less tightly linked, suggesting a more fragmented organization of psychosocial experiences. Across both groups, understanding mental states (mentalization) played a key role, while anxiety had a stronger influence in autism, highlighting possible targets for support and intervention.

## 1 Introduction

Autism spectrum disorder (ASD) offers a unique point of view into the mechanisms that organize social and self‐related experience. Although autism is historically linked to schizophrenia (SCH) through overlapping features of social and cognitive disturbance (Ozbek et al. 2023), ASD is now recognized as a distinct condition; each has its own distinct developmental trajectory and typical age of onset (American Psychiatric Association 2022; Bleuler 1950; Rosen et al. 2021). However, the two conditions share fundamental phenomenological and mechanistic similarities, and their co‐occurrence exceeds chance levels, contributing to poorer prognosis and reduced quality of life (Hossain et al. 2020; Kincaid et al. 2017; Lai et al. 2019): both are characterized by difficulties in managing interpersonal relationships and by pervasive atypicalities in self‐organization (Chisholm et al. 2015; Jutla et al. 2022; Vass et al. 2024). Conceptual frameworks developed in psychosis research have historically informed autism studies (Jutla et al. 2022), while comparisons with neurotypical (NTP) populations may help clarify the broader structure of general psychological vulnerability. When investigating constructs related to self‐ and interpersonal relationships, it is essential to consider the related dimensions of social cognition and functioning.

Differences in social understanding and self‐organization in autism have been linked to various psychosocial constructs that jointly shape interpersonal functioning. Previous research assessing interpersonal dimensions separately has shown that ASD and SCH individuals tend to exhibit lower levels of mentalization or reflective functioning (Chung et al. 2014; Ciaramidaro et al. 2015; Oliver et al. 2021; Tin et al. 2018), greater attachment insecurity (Akdemir et al. 2009; Capps et al. 1994; Sörnyei et al. 2024; Taylor et al. 2008), and reduced perceived social support (Bishop‐Fitzpatrick et al. 2018; Hossain et al. 2020). In non‐clinical populations, these alterations are often accompanied by elevated depression and anxiety symptoms (Buckley et al. 2009; Lai et al. 2019) and higher autistic and schizotypal trait scores (Baron‐Cohen et al. 2001; Kwapil and Barrantes‐Vidal 2015; Ruzich et al. 2015). Self‐related disturbances (ranging from alterations in minimal self‐experience to disruptions in narrative self‐coherence) have also been documented across both autism and psychosis spectra (Henriksen et al. 2025; Sass and Parnas 2003; Vass et al. 2024; Zahavi 2010). Psychological flexibility, an ability to adaptively regulate inner experience, has been found to buffer these difficulties and predict higher well‐being (Aller et al. 2022; Hayes et al. 2006; Levin et al. 2014; Uddin 2021). But how are these constructs related to each other, and what kind of system do they form?

Several theoretical frameworks converge in highlighting the interdependence of the aforementioned psychosocial domains. The mentalization‐based developmental model (Fonagy et al. 2004; Luyten et al. 2020) posits that the capacity to understand self and others arises from early attachment relationships and supports social cognition, affect regulation, and resilience. Empirically, mentalization has been identified as a central transdiagnostic factor associated with symptom severity, interpersonal functioning, and treatment outcome across a range of psychiatric conditions (Bateman et al. 2023; Debbané et al. 2016; Luyten et al. 2020). Ineffective mentalization predicts greater emotional dysregulation (Fonagy et al. 2016), challenges in social adaptation (Chung et al. 2014), and higher depressive and anxiety symptoms in both clinical and subclinical samples (Schwarzer et al. 2021; Taubner et al. 2011). Phenomenological and enactive accounts emphasize the embodied and interactive nature of self‐experience and social understanding (Gallagher 2024; Zahavi 2010), other theoretical models suggest that disturbances in minimal and narrative self‐experience may differentially shape social understanding and self‐organization (Henriksen et al. 2025; Lombardo and Baron‐Cohen 2011; Nelson et al. 2014; Sass and Parnas 2003; Zahavi 2010). Complementing these perspectives, developmental frameworks (Cicchetti and Toth 2009; Debbané et al. 2016; Debbané and Barrantes‐Vidal 2015) conceptualize such processes as mutually influencing elements of a dynamic, multi‐level developmental system, whose atypical configurations may underlie transdiagnostic vulnerability. Within this framework, the disorganized dimension of schizotypy that captures disturbances in thought and behavior organization has been identified as a core marker of such vulnerability (Debbané and Barrantes‐Vidal 2015; Kwapil and Barrantes‐Vidal 2015). It represents a developmental pathway where atypicalities in self‐organization may amplify differences in mentalization and social functioning. By integrating these theoretical perspectives, our approach conceptualizes self‐ and other‐related processes as components of a complex system of mutual influence. This view supports the implementation of a network approach to capture how these interconnected constructs organize the subjective and interpersonal world in autism and related conditions.

Understanding the organization of psychosocial constructs in autism requires tools that can capture the mutual dependencies among multiple interrelated variables. The network approach provides a theoretical and methodological framework for conceptualizing psychological phenomena as systems of interacting components, rather than as manifestations of latent traits (Borsboom and Cramer 2013; Fried et al. 2017; Schmittmann et al. 2013). In this approach, nodes in a network structure represent arbitrary variables, and edges represent statistical interactions between them. The resulting network structure allows for the investigation of predictability and centrality metrics, such as *strength*, *expected influence (EI)*, *closeness*, and *betweenness*, quantifying the relative importance of each node within the system (Borsboom et al. 2021; Bringmann et al. 2019; Haslbeck and Fried 2017; Robinaugh et al. 2020). Highly central nodes may play a major role in maintaining or alleviating patterns associated with mental health and neurodevelopmental conditions (Borsboom and Cramer 2013; Castro et al. 2024). Novel methodological developments make it possible to compare networks between groups by examining differences in overall network connectivity, as well as in centrality and edge weights (van Borkulo et al. 2023). Prior work using network analysis indicates that, compared with typically developing peers, autistic young adults show a sparsely connected social‐cognition network with reduced efficiency, whereas NTP networks are more integrated and highlight Theory of Mind as a central/bridging node when social cognitive capacities were measured (Vagnetti et al. 2020). In the general population, autistic traits and psychotic‐like experiences form distinct communities with bridges through social‐withdrawal (interaction‐difficulty) features (Hajdúk et al. 2023). Complementary evidence from comparative network analysis from ASD and SCH individuals highlights difficulties in interpersonal communication as key aspects for both phenomena (Han et al. 2023). These findings underscore the value of network methods for mapping clinically relevant interdependencies between different conditions, with a particular focus on social cognition and functioning.

Consistent with the network approach commonly used to study mental health, we aimed to examine how constructs regarding self, interpersonal functioning, and vulnerability organized into a complex system in autism, schizophrenia,^1^ and neurotypical populations. First, we expected that autistic and neurotypical participants would differ in the levels of several psychosocial variables, reflecting the well‐documented group differences in social‐ and self‐related functioning. Second, we hypothesized that within the autistic group, mentalization‐related differences and disorganized schizotypal traits would emerge as the most central nodes in the network (Barneveld et al. 2011; Bora 2020; Chung et al. 2014; Polner et al. 2021; Velikonja et al. 2019). We also expected that alterations in the minimal self would be more central in autism compared to its lower relevance in neurotypicals and examined the role of the narrative self in an exploratory manner. Third, we anticipated differences in overall network structure, expecting a more integrated organization of variables representing psychosocial vulnerability, higher global strength, and structure invariance in the autistic group, based on results from network studies of other mental health conditions (Peel et al. 2021; Yokoyama et al. 2022). Through this approach, we aimed to delineate both shared and group‐specific organizational patterns, thereby advancing the understanding of self‐ and social functioning in autism and the broader spectrum of mental health–related processes across symptom dimensions.

## 2 Methods

### 2.1 Participants

Overall 1942 participants completed the entire survey. After preprocessing, data from 1701 participants were included in the analyses: 1483 individuals from the general population (neurotypicals, NTP); 156 individuals with diagnoses of ASD; and 62 individuals with a diagnosis of SCH. The NTP group consisted of individuals who reported neither a formal diagnosis nor a self‐diagnosis of these conditions, nor a first‐degree relative with either diagnosis. In the ASD group (*N* = 156), 48.4% (*n* = 75) reported at least one additional psychiatric diagnosis. The most common co‐occurring diagnoses were mood disorder/depression (40.6%, *n* = 63) and anxiety/panic disorder/phobia (38.7%, *n* = 60). Further diagnoses included ADHD (17.4%, *n* = 27), OCD (16.1%, *n* = 25), eating disorder (12.9%, *n* = 20), and personality disorders (9.7%, *n* = 15). Bipolar disorder (3.2%, *n* = 5), schizophrenia (1.3%, *n* = 2), and Tic/Tourette disorder (0.6%, *n* = 1) were relatively rarely reported, while 3.9% (*n* = 6) selected the “other” category. To ensure comparability between the ASD and NTP groups, we employed a propensity score matching method using the R package *MatchIt* (version 4.7.2.) (Ho et al. 2011). Given the larger NTP sample, nearest neighbor matching was implemented with a 1:3 ratio, whereby each ASD individual was matched to three NTP participants. Propensity scores were estimated based on age, years of education, and educational categories. Additionally, exact matching was enforced on sex (male vs. female) to achieve gender balance. These procedures yielded a matched sample of 156 individuals with ASD and 454 matched NTP participants, which formed the basis for the primary network comparison analyses. Additionally, we conducted a sensitivity analysis in which STAI‐T scores were included in the matching procedure used to create an NTP subsample with comparable trait anxiety level (see Supporting Information: Methods S4). The small size of the SCH group resulted in low stability of its network model, which precluded their inclusion in the main comparative analyses (see Section 3 and Supporting Information for details). Demographics of the full and matched samples are presented in Table 1.

**TABLE 1:** Basic demographic characteristics of the sample.

	Group	Male *n* (%)	Female *n* (%)	Other or NA *n* (%)	Statistics	
					𝟀^2^	*p*
Sex	mNTP (*n* = 454)	216 (47.6%)	216 (47.6%)	22 (4.8%)	1.787	0.409
	ASD (*n* = 156)	72 (46.15%)	72 (46.15%)	12 (7.7%)		

	Group	Min–Max	Mean	SD	Mann–Whitney *U*	*p*	*r* (Effect size)
Age (year)	mNTP	18–56	33.03	8.75	33,926	0.434	0.032
	ASD	18–57	32.42	9.82			
Education (year)	mNTP	8–27	16.39	3.3	33,388	0.284	0.043
	ASD	9–33	16.61	4.0			

Abbreviations: ASD = autism spectrum disorder; mNTP = matched neurotypical control participants, without diagnosis; SD = standard deviation.

### 2.2 Procedure

Our cross‐sectional study was conducted between November 2021 and April 2022. Participants with schizophrenia spectrum diagnosis were included subsequently from January 2022 to April 2024. Recruitment for participation was carried out through media outlets, social media platforms, and direct outreach to outpatient units. Patients with a diagnosis of ASD were recruited from the Outpatient Unit of the Department of Psychiatry and Psychotherapy, Semmelweis University, and from organizations that support autistic individuals. The detailed recruitment procedure for the schizophrenia group is provided in the Supporting Information: Method S1. Overall, participants were recruited using convenience sampling. Individuals were informed about the purpose of the study, and their anonymity was assured, while voluntary consent was obtained to participate. Participants filled out cross‐sectional online self‐report questionnaires. The data collection took place through the formr.org website (Arslan et al. 2020).

The authors assert that all procedures contributing to this work comply with the ethical standards of the relevant national and institutional committees on human experimentation and with the Helsinki Declaration. The study was approved by the Semmelweis University Regional and Institutional Committee of Science and Research Ethics, SE RKEB: 159/2021.

#### 2.2.1 Data Collection: Demographics and Psychological Questionnaires

**FIGURE 1:**
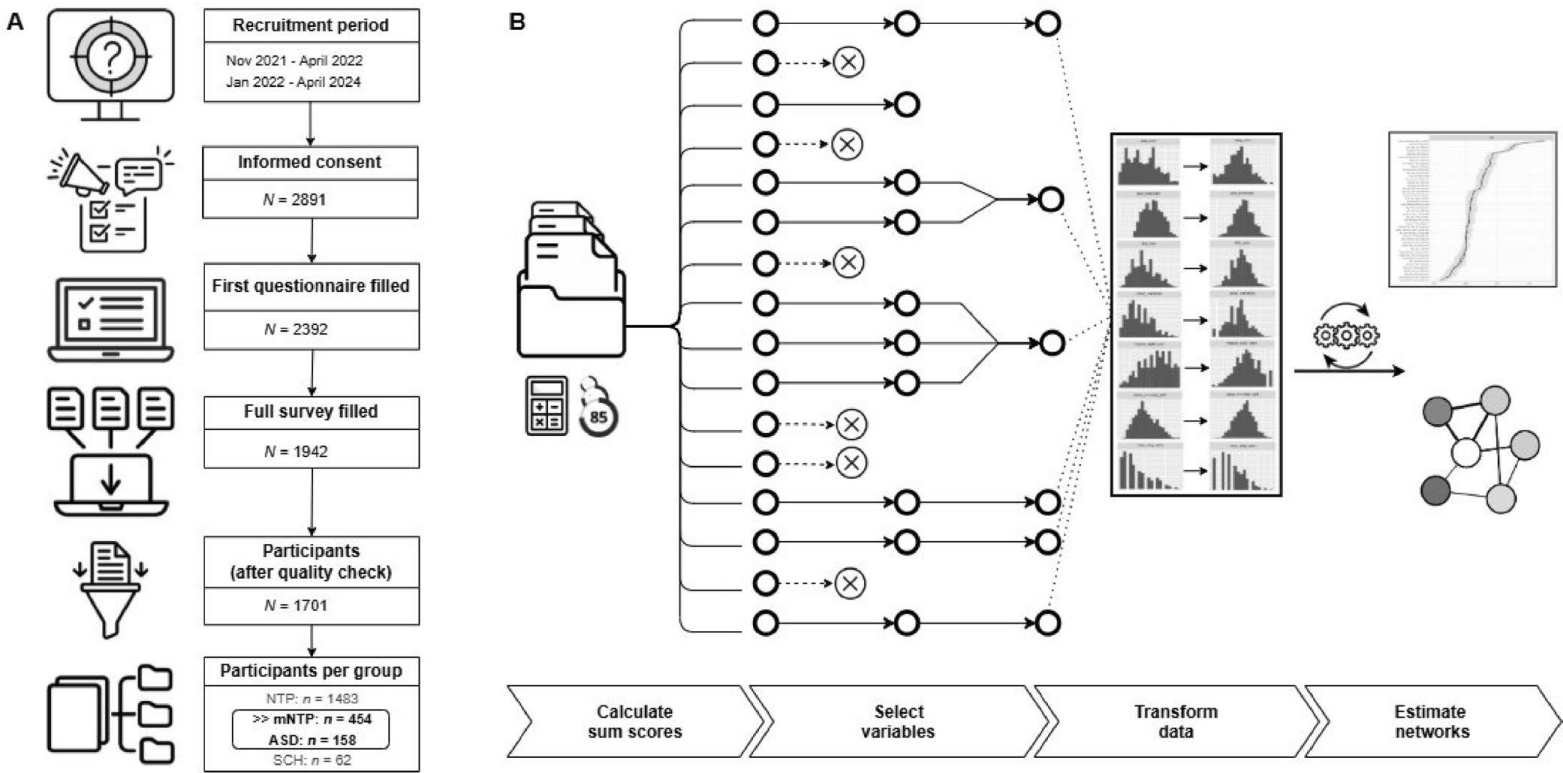
Overview of participant recruitment and data processing pipeline. (A) Final study sample flow chart. (B) Steps of data analysis: Preprocessing, variable selection, transformation, and network estimation.

After providing informed consent, participants provided information on their demographic (see Table 1.) and socioeconomic background (see Tables S1 and S2). Subsequently, participants completed a series of standardized psychological questionnaires assessing the key factors of interest. Autistic traits were assessed using the Autism Spectrum Quotient, AQ‐50 (Baron‐Cohen et al. 2001), while schizotypal traits were measured using the Multidimensional Schizotypy Scale—Brief, MSS‐B (Gross et al. 2018). Mentalization capacities were measured through the Mentalization Questionnaire, MZQ, (Hausberg et al. 2012) and the Reflective Functioning Questionnaire, RFQ (Fonagy et al. 2016), attachment patterns were explored using the Adult Attachment Scale, AAS (Collins 1996), and perceived social support via the Multidimensional Scale of Perceived Social Support, MSPSS (Zimet et al. 1988). Assessing the subjective aspects of self‐perception, we used the Embodied Sense of Self Scale, ESSS (Asai et al. 2016), and self‐concept and cognitive bias were evaluated with the Beck Cognitive Insight Scale, BCIS (Beck et al. 2004). Emotion regulation and psychological flexibility were captured by the Acceptance and Action Questionnaire‐II, AAQ‐II (Bond et al. 2011). Finally, the Beck Depression Inventory (BDI, Beck et al. 1961) and the Trait Anxiety subscale of the State–Trait Anxiety Inventory, STAI‐T (Spielberger et al. 1983) complemented the comprehensive assessment. A detailed variable selection and data preparation process and detailed description of the questionnaires can be found in the Supporting Information: Method S2–S3, and Figures S1–S4. Figure 1 illustrates the recruitment procedure and data preprocessing and processing pipeline.

#### 2.2.2 Statistical Analysis, Network Visualization

All analyses were conducted in R version 4.3.0 (R Core Team 2023) using RStudio (v2023.03.0; RStudio Team, 2023). Gaussian graphical models (GGMs) were estimated separately for each group using regularized methods. This approach models conditionally independent relationships between variables, yielding sparse and interpretable network structures. Networks were estimated using the graphical LASSO regularization (Friedman et al. 2008) combined with Extended Bayesian Information Criterion (EBIC) model selection (Foygel and Drton 2010), addressing the challenge of limited sample sizes. Following methodological recommendations, a standard γ = 0.5 penalization parameter was applied.

#### 2.2.3 Network Centrality Indices

Node characteristics were evaluated using four centrality indices: strength (the sum of absolute edge weights connected to a node), closeness (the inverse of the average shortest path to all other nodes), betweenness (how often a node lies on the shortest path between others), and expected influence (similar to strength, but accounting for the sign of edges) (Bringmann et al. 2019; Epskamp et al. 2018). Centrality metrics were computed and visualized using the *qgraph* and *igraph* packages. Node predictability values, representing the proportion of explained variance (*R* ^2^) for each node, were estimated with the *mgm* package (Haslbeck and Waldorp 2015). Node predictability values and centrality metrics are reported in the Tables S6 and S7.

#### 2.2.4 Network Accuracy and Stability

The accuracy and stability of the estimated networks were evaluated using nonparametric and case‐dropping bootstrap methods implemented in the *bootnet* package (Epskamp et al. 2018). Nonparametric bootstrapping with 10,000 iterations was used to compute 95% confidence intervals (CIs) for edge weights. Case‐dropping bootstrapping assessed the robustness of centrality estimates by calculating the correlation stability (CS) coefficient, which reflects the maximum proportion of cases that can be removed while maintaining a correlation of ≥ 0.7 between subsample and full‐sample centrality values. Following methodological recommendations, CS coefficients ≥ 0.5 were considered preferable for reliable interpretation (Epskamp et al. 2018).

#### 2.2.5 Network Comparison Test (NCT)

Group differences in network structure and connectivity were examined using the *NetworkComparisonTest* package (van Borkulo et al. 2023). The NCT is a permutation‐based test that evaluates network structure invariance (distribution of edge weights), global strength invariance (overall connectivity), and local edge‐weight differences. Centrality invariance tests were additionally performed to compare node‐level strength, expected influence, and closeness across groups. The NCT was conducted with 10,000 permutations, and *p*‐values were adjusted for multiple comparisons using the Benjamini–Hochberg false discovery rate (FDR) procedure.

## 3 Results

### 3.1 Questionnaire Reliability and Group Differences

Internal consistencies of the questionnaires included in the network analysis were acceptable to excellent across groups (Cronbach's *α* = 0.73–0.94; McDonald's *ω* = 0.74–0.95). Full reliability and descriptive results for all questionnaires and subscales are provided in the Tables S3 and S4.

**FIGURE 2:**
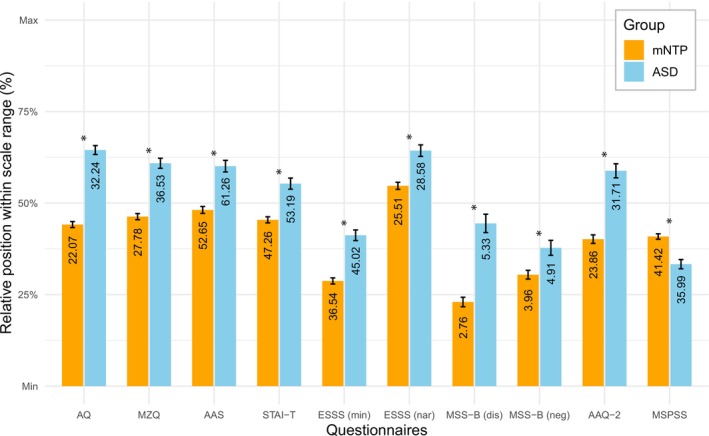
Questionnaire mean scores by group. *Note:* ASD = autism spectrum disorder, mNTP = matched neurotypical controls without psychiatric diagnosis, AQ = Autism Spectrum Quotient, MZQ = Mentalization Questionnaire, AAS = Adult Attachment Scale, STAI‐T = State–Trait Anxiety Inventory, trait anxiety, ESSS (min) and (nar) = Embodied Sense of Self Scale, MSS‐B (dis) and (neg) = Multidimensional Schizotypy Scale—Brief disorganized and negative schizotypy subscales, AAQ‐2 = Acceptance and Action Questionnaire‐II, MSPSS = Multidimensional Scale of Perceived Social Support. Error bars = standard error. Statistical significance for group differences is indicated by *(*p* < 0.05).

Table 2 and Figure 2 summarizes the descriptive statistics and group comparisons across self‐report questionnaires. The ASD group scored significantly higher than the mNTP group on autistic traits, insecure attachment, trait anxiety, psychological inflexibility, schizotypy, and self‐related measures, with medium to large effect sizes (Cohen's *d* = 0.47–1.24), except for negative schizotypy, which showed a small effect (*d* = 0.30). Importantly, mentalization difficulties (MZQ) were markedly greater in the ASD group, with a large effect size (*d* = 0.83). In contrast, they reported lower perceived social support, with a medium effect (*d* = −0.48). The same significant differences were found after STAI‐T matching, except for STAI‐T (see Figure S19 and Table S10). Overall, the results indicate a broad pattern of socio‐emotional and cognitive differences in the ASD group compared to neurotypical controls. (For detailed description of all questionnaires, subscales, and data from the SCH group, see Table S4.)

**TABLE 2:** Descriptive statistics and group comparisons for psychological variables included in the final analysis.

Variable	Group	Min—Max	Mean	SD	Mann–Whitney *U*	*p*	*r (Effect size)*
Autistic traits (AQ‐50)	mNTP	4–48	22.07	8.74	*W* = 57,016	< 0.001	0.46
	ASD	12–49	32.24	7.63			
Mentalization (MZQ)	mNTP	2–54	27.78	10.82	*W* = 50,524	< 0.001	0.32
	ASD	13–60	36.53	10.33			
Insecure attachment (AAS)	mNTP	22–90	52.65	14.37	*W* = 46,862	< 0.001	0.24
	ASD	30–90	61.26	14.24			
Trait anxiety (STAI‐T)	mNTP	25–77	47.26	10.64	*W* = 46,168	< 0.001	0.23
	ASD	25–77	53.19	11.31			
Minimal self (ESSS)	mNTP	17–73	36.54	12.21	*W* = 48,980	< 0.001	0.29
	ASD	21–77	45.02	12.34			
Narrative self (ESSS)	mNTP	9–40	25.51	6.59	*W* = 45,056	< 0.001	0.21
	ASD	10–40	28.58	6.33			
Negative schizotypy (MSS‐B)	mNTP	0–13	3.96	3.31	*W* = 41,606	0.001	0.13
	ASD	0–12	4.91	3.32			
Disorganized schizotypy (MSS‐B)	mNTP	0–12	2.76	3.31	*W* = 50,164	< 0.001	0.32
	ASD	0–12	5.33	3.77			
Perceived social support (MSPSS)	mNTP	12–60	41.42	11.37	*W* = 25,653	< 0.001	0.21
	ASD	12–60	35.99	11.39			
Psychological inflexibility (AAQ‐2)	mNTP	7–49	23.86	10.46	*W* = 50,182	< 0.001	0.32
	ASD	7–49	31.71	10.09			

*Note:* Higher scores reflect greater trait expression or symptom severity on all measures except perceived social support, where higher scores indicate greater support.

Abbreviations: AAQ‐2 = acceptance and action questionnaire‐II; AAS = Adult Attachment Scale; AQ = autism spectrum quotient; ASD = autism spectrum disorder; ESSS = Embodied Sense of Self Scale; mNTP = matched neurotypical controls without psychiatric diagnosis; MSPSS = Multidimensional Scale of Perceived Social Support; MSS‐B = Multidimensional Schizotypy Scale‐Brief; MZQ = Mentalization Questionnaire; SD = standard deviation; STAI‐T = state–trait anxiety inventory, trait anxiety.

### 3.2 Network Structure

**FIGURE 3:**
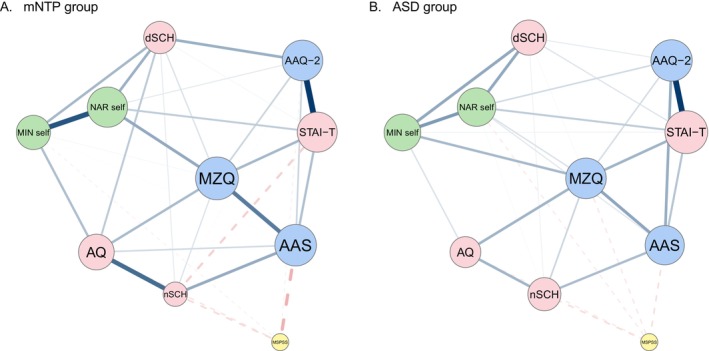
Visual representation of the partial correlation network structures of the two groups. A. Network structure of mNTP group, B. Network structure of ASD group. *Note:* Each node represents a psychological construct, and edges represent regularized partial correlations between them. Blue edges indicate positive associations, while red edges indicate negative associations. The thickness of the edge corresponds to the magnitude of the partial correlation. Node size reflects the predictability of each variable. Node colors were assigned to visually distinguish conceptual domains within the network: Psychopathology‐related traits (pink), dimensional psychological factors (blue), self‐related constructs (green), and perceived social support (yellow). The colors are intended for interpretive clarity and do not reflect statistical clustering. AAQ‐2 = psychological inflexibility, AAS = insecure attachment, AQ = Autism Spectrum Quotient, ASD = autism spectrum disorder, dSCH = disorganized schizotypy, MIN self = minimal self, mNTP = matched neurotypical controls without psychiatric diagnosis, MSPSS = perceived social support, MZQ = mentalization, NAR self = narrative self, nSCH = negative schizotypy, STAI‐T = trait anxiety.

In our regularized partial correlation networks, nodes represent the variables for the questionnaire scale and subscale sum scores. Node size represents the predictability of the variable. Blue edges represent positive partial correlation coefficients, while red dashed ones indicate negative correlations. The strength of the correlation between two variables is indicated by the thickness of the edge. For visualization, we first applied the Fruchterman–Reingold algorithm (Fruchterman and Reingold 1991) to the mNTP group. In this layout, nodes with the highest number of connections in the mNTP network are placed centrally, while less connected nodes are located on the periphery. This arrangement was then fixed and applied to the ASD (and SCH, see in Figure S18.) network to allow direct visual comparison. The partial correlation networks for the two groups are shown in Figure 3.

The partial correlation networks of the mNTP and ASD groups showed broadly similar overall configurations, with most edges and node arrangements appearing comparable across the two groups (Figure 3A,B, edge weights reported in Table S5). Nevertheless, several noteworthy descriptive differences can be observed. In the mNTP network, relatively strong positive associations were visible between disorganized schizotypy (dSCH) and psychological inflexibility (AAQ‐2), as well as between dSCH and autistic traits (AQ), whereas these connections were weak or absent in the ASD network. Moreover, the direct edge between trait anxiety (STAI‐T) and negative schizotypy (nSCH) was missing in the ASD group. Insecure attachment (AAS) also showed fewer and weaker connections in the ASD network compared to the mNTP group. In the NTP group, impaired mentalization (MZQ) was connected to narrative self disturbances (NAR self), while in ASD its link with minimal self alterations (MIN self) was stronger. These descriptive patterns suggest subtle differences in how dimensional and psychological vulnerability related constructs interrelate across groups. Given the exploratory nature of the analysis, these visual observations should be interpreted cautiously; formal statistical testing of node level network invariance is reported in the Section 2.2.5.

#### 3.2.1 Accuracy and Stability of the Network

To ensure the reliability of the estimated network structures, we assessed the accuracy and stability of edge weights and centrality indices using bootstrapping methods. The nonparametric bootstrap indicated that edge‐weight estimates were generally stable in the mNTP and ASD networks. Edge‐weight accuracy was highest in the mNTP group, acceptable in the ASD group, and limited in the SCH group.

According to the recommended criterion of a CS coefficient ≥ 0.50 (Epskamp et al. 2018), both the mNTP and ASD networks reached adequate levels of stability for most indices, whereas the SCH network was largely unstable. The mNTP network showed high stability for expected influence (CS = 0.9), strength (CS = 0.71), and closeness (CS = 0.52), with only betweenness falling below the threshold (CS = 0.24). In the ASD network, CS‐coefficients indicated moderate stability for expected influence (CS = 0.52) and strength (CS = 0.52), while closeness was above the minimum requirement (> 0.25) for interpretation yet still below the desirable level (CS = 0.33), and betweenness exhibited notably low stability (CS = 0.15). For the SCH network, none of the centrality metrics achieved sufficient stability; figures and tables illustrating these results are provided in the Figures S9–S17, Table S8; which led to the exclusion of the SCH network from the final analyses.

**FIGURE 4:**
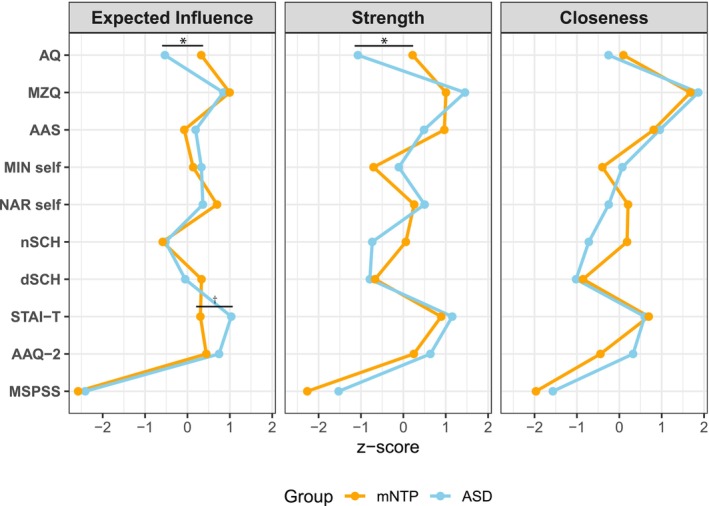
Node centralities of the estimated network models. Centrality indices are reported as standardized z‐values. *Note:* ASD = autism spectrum disorder, mNTP = matched neurotypicals, AQ = Autism Spectrum Quotient, dSCH = disorganized schizotypy, nSCH = negative schizotypy, STAI‐T = trait anxiety, AAQ‐2 = psychological inflexibility, AAS = insecure attachment, MZQ = mentalization, MIN self = minimal self, NAR self = narrative self. Statistical significance is indicated by *(*p* ≤ 0.05), and trend‐level effects by ^†^(*p* ≤ 0.1).

Overall, in both the mNTP and ASD networks, expected influence and strength represent the most robust and interpretable centrality indices; closeness should be interpreted with caution (Figure 4 and Table S8), while betweenness for the two groups and any metrics of the SCH network are not suitable for interpretation due to instability.

#### 3.2.2 Centrality Metrics

Given the exploratory nature of the present study, centrality results should be interpreted descriptively rather than inferentially. Z‐scores are presented to illustrate the pattern of node importance within each group, rather than to support formal statistical comparisons. Nonetheless, these relative configurations may offer meaningful insights into how psychological constructs play different roles across groups, highlighting distinct organizing factors.

In the mNTP network, MZQ had the highest strength and expected influence z‐scores, followed by AAS. Notably, AQ also showed relatively high z‐scores compared to the ASD group. Disorganized schizotypy showed slightly higher expected influence, but lower strength, closeness in the mNTP network, and lower overall centrality in the ASD network. Within the ASD network, the nodes with the highest strength z‐scores were MZQ, followed by STAI‐T and AAQ‐II, while regarding expected influence STAI‐T reached highest z‐scores, followed by MZQ and AAQ‐II. While self‐referential constructs (MIN self, NAR self) and attachment anxiety (AAS) contributed moderately, with slightly different weights in the two groups. In line with the centrality metrics, in the mNTP network MZQ showed the highest node predictability values, followed by AAS and AAQ‐2, whereas in the ASD network STAI‐T node has the highest predictability, followed by AAQ‐2 and MZQ. Interestingly, in the ASD network perceived social support (MSPSS) indicated negligible predictability value. Complete centrality metrics and node predictability values for all nodes across groups are reported in Tables S5 and S6.

#### 3.2.3 Network Comparison Tests

We conducted Network Comparison Tests (NCT) to statistically evaluate differences between the mNTP and ASD networks, focusing on global strength, network structure, individual edge weight differences, and centrality invariance. The mNTP network showed significantly greater global strength than the ASD network (Δ = 0.773, *p* = 0.003), indicating globally weaker associations among variables in ASD. In contrast, the overall network structure did not differ significantly between groups (network structure invariance: *M* = 0.172, *p* = 0.469). Edge invariance tests did not reveal significant between‐group differences after correction; for descriptive purposes, the group‐specific correlation matrices are provided in the Figures S5–S7, illustrating the overall pattern of associations in each group. Centrality invariance tests suggested that AQ were more central in the mNTP compared to the ASD network (strength: Δ = −0.459, *p* = 0.027; EI: Δ = −0.431, *p* = 0.047; and trend in closeness: Δ = −0.004, *p* = 0.089) due to the lower stability of closeness, results can be interpreted cautiously (see Table S9). Moreover, STAI‐T showed a trend to greater EI in ASD (Δ = 0.265, *p* = 0.096), and disorganized schizotypy showed a trend to greater strength in the mNTP group (Δ = −0.224, *p* = 0.096). No other centrality differences reached significance after Benjamini–Hochberg correction.

Overall, these findings indicate that although the two groups share a broadly similar connectivity pattern, the ASD network is characterized by weaker global connectivity and possible shifts in the centrality of autistic traits (reduced) and anxiety (increased). IIt is important to note that these results hold even after controlling for anxiety levels, suggesting that the differences between the NTP and autistic samples are not modulated by anxiety (see Figures S20 and S21 for visualization of the network model and corresponding centrality results, Table S11. for node‐level network invariance). Global strength differences remained significant in the anxiety‐matched analysis (Δ = 0.586, *p* = 0.003). Significant centrality differences (and tendencies) were also observed (Table S11). The clearest finding was that AQ strength remained significantly different between groups in both the original and the STAI‐matched analyses (original: *p* = 0.027; STAI‐matched: *p* = 0.021). Furthermore, the STAI‐matched comparison remained at a tendency level for expected influence (*p* = 0.074) and additionally for closeness (*p* = 0.069). Taken together, these findings suggest that the between‐group differences in network structure were not primarily driven by elevated trait anxiety. These results highlight the robustness of our results regarding the ASD‐NTP comparison.

## 4 Discussion

In this study, we applied network analysis to examine social‐cognitive, self‐conceptual, and resilience‐related constructs in diagnosed autistic individuals and a demographically matched neurotypical comparison group. Results from a small schizophrenia subsample were finally excluded due to insufficient model stability. Across all variables, the autistic group consistently exhibited values indicative of higher psychological vulnerability. The network analysis revealed notable differences in centrality and network connectivity of the measured constructs. Consistent with our hypothesis, mentalization emerged as the most central node in the mNTP network across all centrality indices and also showed the highest strength centrality within the ASD network. In contrast, disorganized schizotypy did not assume a particularly central position in either group. Interestingly, autistic traits occupied a significantly higher centrality in the mNTP network, whereas trait anxiety tended to be more central in the ASD network. The mNTP network exhibited significantly higher global strength, with weaker global connectivity in the ASD network indicating a more fragmented interplay between constructs.

Autistic traits (AQ) showed a significantly higher centrality in the neurotypical (NTP) group than in the ASD group, suggesting that in the general population individual differences in autistic traits are more deeply embedded within a broader constellation of psychosocial processes. This pattern aligns with dimensional models positing that autism‐related characteristics are continuously distributed and meaningfully shape social‐cognitive variability well below clinical thresholds (Baron‐Cohen et al. 2001; Ruzich et al. 2015). Prior network studies in non‐clinical samples similarly indicate that autistic traits form tightly connected clusters with social‐withdrawal and interaction‐difficulty features and often function as bridges between psychosocial domains (Hajdúk et al. 2023; Vagnetti et al. 2020). Importantly, this dimensional embedding of autistic traits is consistent with findings from psychosis research showing that autism‐like characteristics in schizophrenia are closely linked to social functioning outcomes and may reflect shared mechanisms of interpersonal vulnerability (Isvoranu et al. 2022). In this sense, our mNTP network represents a convergence of theoretical expectation and empirical structure: autistic traits emerged as a central organizing variable of the social‐cognitive phenotype. These findings might support a quasi‐dimensional model in which autistic traits strongly co‐vary with socio‐cognitive functioning in the general population, but this coupling reorganizes once ASD becomes clinically manifest: introducing a potential conceptual threshold between dimensional trait expression and the clinical ASD phenotype.

Our findings are compatible with recent conceptual critiques of a strictly linear spectrum view of autism, which argue that while autism‐related characteristics may vary dimensionally in the population, clinical autism represents a qualitatively distinct condition rather than a simple extreme of trait distribution (Mottron et al. 2025). Clinically, such a distinction is highly relevant: similar social‐cognitive differences may arise for different functional reasons in NTP versus ASD groups. In NTP individuals, ineffective mentalization may reflect the dimensional expression of autistic traits (Schuwerk et al. 2019; Wainer et al. 2011), and might be associated with insecure attachment (Gallitto and Leth‐Steensen 2015; Luyten et al. 2020) and self disturbances (Osterhaus et al. 2025), whereas in autistic individuals social‐cognitive difficulties may be related more strongly to anxiety (Cai et al. 2018; Hollocks et al. 2019; Sharma et al. 2014) and linked more to minimal self alterations (Gillespie‐Smith et al. 2018). Accordingly, in ASD, anxiety often exerts a greater influence on general functioning and subjective distress than core autistic features themselves (Bishop‐Fitzpatrick et al. 2015; Lai et al. 2019).

In conclusion, our findings suggest that considering the severity and role of anxiety may be highly relevant in determining the clinical threshold regarding ASD. Sensitivity analysis revealed that including STAI‐T scores in the group‐matching procedure did not eliminate the difference in centrality (expected influence), suggesting that high levels of trait anxiety occupy different positions within the psychopathological network in autistic and non‐autistic individuals. From a clinical perspective, this raises the possibility that anxiety in autistic individuals may not be fully equivalent in form or phenomenology to anxiety as typically observed in neurotypical individuals. Nevertheless, the additional matching analysis provides further support for the robustness of our main findings. Still, our models show cross‐sectional associations, so we cannot directly infer causal relationships from them. Results are rather mixed regarding the causal relationship between sociocognitive abilities and anxiety; some studies support a bidirectional or multifactorial account, with autistic traits and social competence linked to anxiety, alongside sensory intolerance, rigidity, and arousal dysregulation as key contributors, in line with meta‐analytic evidence showing anxiety disorder rates of 40%–50% among autistic youth (Hollocks et al. 2019; Lai et al. 2019; Liew et al. 2015; Postorino et al. 2017). In contrast, other studies point to a more unidirectional role of anxiety, predicting later social‐communication impairment and reduced emotion recognition and affective empathy independent of diagnosis (Duvekot et al. 2018; Lassalle et al. 2019), with network, transdiagnostic, and neurobiological evidence consistently highlighting the centrality of arousal‐related processes and anxiety‐linked network dysfunction in shaping social‐functional outcomes (Chen et al. 2021; Cheng et al. 2020; Isvoranu et al. 2022; Montazeri et al. 2020; Wang and Li 2023). Overall, in light of these results, our findings suggest that greater fragmentation of psychosocial processes in ASD may allow anxiety to exert a stronger organizational influence, highlighting affective dysregulation as a potentially relevant clinical target (Loftus et al. 2023), while acknowledging that evidence for downstream social‐cognitive effects remains preliminary. Importantly, these analyses were exploratory in nature, and anxiety was not a priori hypothesized; accordingly, the present conclusions should be considered hypothesis‐generating and warrant replication using confirmatory network approaches (Du et al. 2025).

The ASD network showed significantly lower global strength than the mNTP network, indicating that although social‐cognitive differences were more pronounced at the level of individual domains, these impairments were less tightly interconnected. This pattern suggests a relatively less integrated social‐cognitive organization in ASD at the level of estimated network structure, characterized by weaker statistical coupling among alterations across individual differences rather than a highly cohesive system. Developmental models of mentalization emphasize its role in supporting coherent self‐ and other‐related representations (Fonagy et al. 2004); accordingly, reduced global integration may reflect disruption in such higher‐order organizing mechanisms. Notably, despite the fragmented structure, mentalization emerged as one of the most central nodes in the ASD network, suggesting that even under conditions of atypical global integration, mentalizing capacity may retain a key bridging or compensatory role. This pattern aligns with Mentalization‐Based Therapy frameworks and supports the hypothesis that mentalization may function as a resilience‐promoting factor and a particularly relevant therapeutic target in ASD (Costa‐Cordella et al. 2023). Importantly, this finding should be interpreted in light of network psychopathology research showing that the meaning of connectivity depends on the nature of the modeled variables (Bringmann et al. 2022; Robinaugh et al. 2020). While prior ASD network studies focusing on social‐cognitive abilities and performance‐based measures have reported weaker connectivity, potentially reflecting coordinated or compensatory engagement (Vagnetti et al. 2020), the present study modeled psychological vulnerability and symptom dimensions, for which stronger connectivity may instead reflect pathological coupling or mutual reinforcement. From this perspective, weaker connectivity among domains in ASD might point to a distinct organization of psychological vulnerability, reflecting a difference in integrative mechanisms consistent with theories proposing that social cognition in ASD is less hierarchically organized and more domain‐specific (Bernhardt et al. 2025; Leekam 2016). These results are accompanied by the observed comparable pattern in the perceived social support that may be shaped by social desirability and retrospective memory biases, particularly in clinical groups (Gernsbacher et al. 2020; Schneid and Raz 2020), especially when assessed with reference to adolescence. Furthermore, perceived social support reflects subjective experience and may not fully align with the support others intend to provide, underscoring that support is co‐constructed and may be differently expressed and interpreted across individuals. However, as reduced global strength could partly reflect lower model stability, the theoretical coherence of the results highlights the need for longitudinal and intervention‐based studies to test whether enhancing mentalization can increase network integration and adaptive functioning in ASD.

Although edges between disorganization and self‐related variables can be seen in both networks, neither showed higher centrality within the two networks or in comparisons between them. Other studies investigating etiology and structure of symptoms of psychosis spectrum disorders and vulnerability showed disorganization's high centrality (Brasso et al. 2023; Christensen et al. 2018, 2019; Li et al. 2025; Peralta et al. 2020; Polner et al. 2021; Polner, Faiola, et al. 2021). It is therefore plausible that the central importance of disorganization is more relevant in the development and core symptoms in the psychosis‐spectrum, but not in social cognition in the general population and ASD.

### 4.1 Limitations and Further Directions

Despite several strengths, a number of limitations should be acknowledged. First, the cross‐sectional design precludes conclusions regarding temporal ordering or causal relationships among the studied variables. Second, the exclusive reliance on self‐report measures introduces inherent constraints, particularly in conditions where insight and self‐awareness may be compromised. This limitation is especially relevant for mentalization, as recent evidence suggests that individuals with greater mentalizing difficulties may overestimate their own capacities (Wendt et al. 2024). In addition, sample bias cannot be ruled out, as individuals with higher psychological vulnerability may have been less likely to complete the questionnaires, potentially limiting the representation of symptom severity and contributing to restricted variability.

Methodological considerations specific to network analysis should also be noted. Network estimates were necessarily constrained by sample size and stability requirements, which limited the number of variables that could be reliably included. Moreover, as the present networks were estimated at the between‐person level, they reflect patterns of covariation across individuals rather than dynamic interactions within individuals over time, and centrality metrics should therefore not be interpreted as direct indicators of causal influence.

Future research would benefit from integrating multimethod assessment strategies, including clinician‐rated measures, informant reports, and behavioral or neuropsychological tasks, to complement self‐report data and mitigate insight‐related biases. Longitudinal (e.g., experience sampling methods) and intervention‐based designs are particularly warranted to examine whether changes in central processes such as mentalization lead to shifts in overall network organization and adaptive functioning. Despite their exploratory nature, these findings may serve as a foundation for future dimensional investigations of the structural organization of social‐cognitive dysfunction across diagnostic boundaries.

### 4.2 Conclusion

Using a network approach, this study demonstrates that social‐cognitive and psychosocial processes in autism spectrum disorder show a distinct organizational pattern characterized by lower global integration and differential centrality of key constructs compared to neurotypical individuals: mentalization emerged as a central organizing process across groups, while autistic traits exhibited higher centrality in the neurotypical network, and trait anxiety showed a tendency toward higher centrality in the ASD network. Taken together, these findings support dimensional but non‐linear models of autism and underscore mentalization and, to a more tentative extent, affective regulation, as promising targets for transdiagnostic and mechanism‐focused intervention research.

## Author Contributions

S.H.: methodology, software, investigation, formal analysis, data curation, writing – original draft, visualization. D.S.: investigation, writing – review and editing. Á.V.: formal analysis, writing – review and editing. L.R.: validation, data curation, writing – review and editing, supervision. K.F. conceptualization, methodology, software, investigation, resources, writing – review and editing, visualization, supervision, funding acquisition and project administration.

## Funding

This research was supported by the Hungarian National Research, Development and Innovation Office (NKFI/OTKA PD 146424) (to K.F. and S.H.) and by the University Excellence Fund of Eötvös Loránd University (to L.R.) and the Hungarian National Research, Development and Innovation Office (NKFI/OTKA FK 142765) (to L.R.).

## Ethics Statement

This study was approved by the Semmelweis University Regional and Institutional Committee of Science and Research Ethics, approval number SE RKEB: 159/2021.

## Consent

Informed consent was obtained from all participants for this study. Participants indicated their consent to participate by clicking the provided button after reading the informed consent form.

## Conflicts of Interest

The authors declare no conflicts of interest.

## Supporting information


**Table S1:** Detailed socioeconomic characteristics of the total sample.
**Table S2:** Receipt of psychological support by group.
**Table S3:** Questionnaire reliability: Internal consistency.
**Table S4:** Descriptive and reliability statistics of all study variables by group.
**Table S5:** Edge weights across groups.
**Table S6:** Node predictability values.
**Table S7:** Node centrality metrics (strength, expected influence, closeness, and betweenness) across diagnostic groups.
**Table S8:** Correlation Stability (CS) coefficients.
**Table S9:** Node‐level network invariance.
**Table S10:** Descriptive statistics and group comparisons for psychological variables included in the analysis.
**Table S11:** Node‐level network invariance.
**Figure S1:** Distribution of questionnaire scores in the mNTP sample—pre‐ and post‐transformation.
**Figure S2:** Distribution of questionnaire scores in the ASD sample—pre‐ and post‐transformation.
**Figure S3:** Distribution of questionnaire scores in the SCH sample—pre‐ and post‐transformation.
**Figure S4:** Distribution of questionnaire scores in the NTP sample—pre‐ and post‐transformation.
**Figure S5:** Correlation matrix of variables in the matched NTP group.
**Figure S6:** Correlation matrix of variables in the ASD group.
**Figure S7:** Correlation matrix of variables in the SCH group.
**Figure S8:** Node centrality metrics (strength, expected influence, closeness, and betweenness) across diagnostic groups.
**Figure S9:** Case dropping bootstrap in matched NTP group.
**Figure S10:** Case dropping bootstrap in ASD group.
**Figure S11:** Case dropping bootstrap in SCH group.
**Figure S12:** Bootstrapped confidence intervals of all edges and stability test for edge‐weight differences in mNTP group.
**Figure S13:** Bootstrapped difference tests between node centralities in mNTP group.
**Figure S14:** Bootstrapped confidence intervals of all edges and stability test for edge‐weight differences in the ASD group.
**Figure S15:** Bootstrapped difference tests between node centralities in the ASD group.
**Figure S16:** Bootstrapped confidence intervals of all edges and stability test for edge‐weight differences in SCH group.
**Figure S17:** Bootstrapped difference tests between node centralities in the SCH group.
**Figure S18:** The partial correlation network structures of the three groups.
**Figure S19:** Questionnaire mean scores by group (ASD_STAI vs. mNTP_STAI).
**Figure S20:** Visual representation of the partial correlation network structure of the mNTP_STAI group.
**Figure S21:** Node centralities of the estimated STAI matched network models.

## Data Availability

The data and analysis pipeline that support the findings of this study are available on the Open Science Framework (OSF) at: https://osf.io/jsknp/overview?view_only=2f1c8ade959d47ed8c59b649762f1f69.

## Endnotes

^1^
Due to stability issues, the network model of the SCH group was not interpreted. Hypotheses and results for the SCH group are provided in the Supporting Information.
