# Supplementary material for: The Network Model of Mentalization, Social Vulnerability, and the Self in Autism: A Comparison With Neurotypical Adults
Source: Autism Res. 2026 Jun 16;19(7):e70263. doi: 10.1002/aur.70263 (PMC13377338; doi:10.1002/aur.70263)
Supplement: Supplementary file 1 — Table S1: Detailed socioeconomic characteristics of the total sample. Table S2: Receipt of psychological support by group. Table S3: Questionnaire reliability: Internal consistency. Table S4: Descriptive and reliability statistics of all study variables by group. Table S5: Edge weights across groups. Table S6: Node predictability values. Table S7: Node centrality metrics (strength, expected influence, closeness, and betweenness) across diagnostic groups. Table S8: Correlation Stability (CS) coefficients. Table S9: Node‐level network invariance. Table S10: Descriptive statistics and group comparisons for psychological variables included in the analysis. Table S11: Node‐level network invariance. Figure S1: Distribution of questionnaire scores in the mNTP sample—pre‐ and post‐transformation. Figure S2: Distribution of questionnaire scores in the ASD sample—pre‐ and post‐transformation. Figure S3: Distribution of questionnaire scores in the SCH sample—pre‐ and post‐transformation. Figure S4: Distribution of questionnaire scores in the NTP sample—pre‐ and post‐transformation. Figure S5: Correlation matrix of variables in the matched NTP group. Figure S6: Correlation matrix of variables in the ASD group. Figure S7: Correlation matrix of variables in the SCH group. Figure S8: Node centrality metrics (strength, expected influence, closeness, and betweenness) across diagnostic groups. Figure S9: Case dropping bootstrap in matched NTP group. Figure S10: Case dropping bootstrap in ASD group. Figure S11: Case dropping bootstrap in SCH group. Figure S12: Bootstrapped confidence intervals of all edges and stability test for edge‐weight differences in mNTP group. Figure S13: Bootstrapped difference tests between node centralities in mNTP group. Figure S14: Bootstrapped confidence intervals of all edges and stability test for edge‐weight differences in the ASD group. Figure S15: Bootstrapped difference tests between node centralities in the ASD group. Figure S16: Bootstrapped confi [file AUR-19-0-s001.docx]

**Supplementary materials**

The network model of mentalization, social vulnerability and the self in autism: a comparison with neurotypical adults

Szilárd Holka^1, 2^, Dániel Sörnyei^1, 3^, Ágota Vass^1,4^, Levente Rónai^5, 6,*^, Kinga Farkas^1^

^1^ Department of Psychiatry and Psychotherapy, Semmelweis University, Budapest, Hungary

^2^ HUN-REN Institute of Cognitive Neuroscience and Psychology, Research Centre of Natural Sciences, Budapest, Hungary

^3^ Department of Clinical Psychology, Semmelweis University, Budapest, Hungary

^4^ University Research and Innovation Center, Physiological Controls Research Center; and John von Neumann Faculty of Informatics, Óbuda University, Budapest, Hungary

^5^ Institute of Psychology, ELTE Eötvös Loránd University, Budapest, Hungary

^6^ Institute of Psychology, University of Szeged, Szeged, Hungary

*** corresponding author: Levente Rónai
e-mail: ronai.levente@elte.ppk.hu
address: Institute of Psychology, ELTE Eötvös Loránd University, Budapest, Hungary

[**Methods 2**](#_heading=h.4okxbu5unfen)

[Method S1. Recruitment Procedure for the Schizophrenia Group 2](#_heading=h.mh217z4gau5y)

[Method S2. Variable selection and data preparation 2](#_heading=h.kyiahx7r645h)

[**Sample characteristics 4**](#_heading=h.7l79mw4ri5ed)

[Table S1. Detailed socioeconomic characteristics of the total sample 4](#_heading=h.rhioalte4ed1)

[Table S2. Receipt of psychological support by group 5](#_heading=h.3apnofw9umk9)

[**Questionnaires 5**](#_heading=h.deop3xke46w7)

[Method S3. Description of the instruments included in the final network analysis 5](#_heading=h.tkr8toykt2uk)

[Psychometric properties 7](#_heading=)

[Table S3. Questionnaire reliability: Internal consistency 7](#_heading=h.oui8hqpflj1l)

[Table S4. Descriptive and reliability statistics of all study variables by group 8](#_heading=h.emr2jtw7ipp2)

[Non-paranormal transformation 12](#_heading=h.8srhaho7bjw5)

[Figure S1. Distribution of questionnaire scores in the mNTP sample - pre- and post-transformation 12](#_heading=h.pb9vpzfgrj0o)

[Figure S2. Distribution of questionnaire scores in the ASD sample - pre- and post-transformation 13](#_heading=h.djnwqm9ar292)

[Figure S3. Distribution of questionnaire scores in the SCH sample - pre- and post-transformation 14](#_heading=h.ho37jhhne804)

[Figure S4. Distribution of questionnaire scores in the NTP sample - pre- and post-transformation 15](#_heading=h.eosj6u100s5c)

[**Network analysis 16**](#_heading=h.8ktkdyz0t7u)

[Table S5. Edge weights across groups 16](#_heading=h.x03b349rpzjd)

[Figure S5. Correlation matrix of variables in the matched NTP group 17](#_heading=h.3q49qqiyyvwr)

[Figure S6. Correlation matrix of variables in the ASD group 17](#_heading=h.h94uk4kds8e3)

[Figure S7. Correlation matrix of variables in the SCH group 18](#_heading=h.1la6fi56gbe4)

[Node predictability 18](#_heading=h.cv8uhrq8idzp)

[Table S6. Node predictability values 18](#_heading=h.3cjri5nyvj2h)

[Centrality metrics 19](#_heading=h.8loi1t9glz6h)

[Table S7. Node centrality metrics (strength, expected influence, closeness, and betweenness) across diagnostic groups 19](#_heading=h.6gmmnk1wd8gh)

[Figure S8. Node centrality metrics (strength, expected influence, closeness, and betweenness) across diagnostic groups 21](#_heading=h.yfiv14ksdokf)

[Stability 21](#_heading=h.19gio5exainh)

[Figure S9. Case dropping bootstrap in matched NTP group 21](#_heading=h.fawpswach557)

[Figure S10. Case dropping bootstrap in ASD group 22](#_heading=h.7r9iecfv7y6o)

[Figure S11. Case dropping bootstrap in SCH group 22](#_heading=h.um63pmvfbba)

[Figure S12. Bootstrapped confidence intervals of all edges and stability test for edge-weight differences in mNTP group 23](#_heading=h.j24jg3aeacyv)

[Figure S13. Bootstrapped difference tests between node centralities in mNTP group 24](#_heading=h.bzv1i1dh4m4y)

[Figure S14. Bootstrapped confidence intervals of all edges and stability test for edge-weight differences in the ASD group 25](#_heading=h.vxcfqa9pmznp)

[Figure S15. Bootstrapped difference tests between node centralities in the ASD group 26](#_heading=h.vb1r0xuh96ei)

[Figure S16. Bootstrapped confidence intervals of all edges and stability test for edge-weight differences in SCH group 27](#_heading=h.48olqik4lh8u)

[Figure S17. Bootstrapped difference tests between node centralities in the SCH group 28](#_heading=h.vdvzixu6zcen)

[Table S8. Correlation Stability (CS) coefficients 28](#_heading=h.bkt3bbmrzafj)

[**Network comparison 29**](#_heading=h.i8l4egvstl5i)

[Table S9. Node-level network invariance 29](#_heading=h.5cv3x86f7581)

[Figure S18. The partial correlation network structures of the three groups 30](#_heading=h.bs9yinpcdl78)

[**Sensitivity analysis 31**](#_heading=h.sch9xdy02za8)

[Methods S4. 31](#_heading=h.3w46gq93c4z9)

[Figure S19. Questionnaire mean scores by group (ASD_STAI vs. mNTP_STAI) 32](#_heading=h.wltw318nsd3h)

[Table S10. Descriptive statistics and group comparisons for psychological variables included in the analysis 32](#_heading=h.co2kdgpc5w5v)

[Figure S20. Visual representation of the partial correlation network structure of the mNTP_STAI group 34](#_heading=h.ctfsuthq1n7s)

[Figure S21. Node centralities of the estimated STAI matched network models 35](#_heading=h.pzjm0iycdzwk)

[Table S11. Node-level network invariance 36](#_heading=h.uxnthud3o6kg)

# Methods

### Method S1. Recruitment Procedure for the Schizophrenia Group

Participants with a diagnosis of schizophrenia (SCH) were recruited from among patients under psychiatric care at the Department of Psychiatry and Psychotherapy, Semmelweis University, later due to the low participation tendency, additional participants were recruited from external daycares and other rehabilitation institutions, with 14 of them completing a paper version of the questionnaire package. In the case of participants under legal guardianship, 3 of whom were included in the sample, the consent of the guardian was also obtained. Our hypotheses regarding the schizophrenic group, which we were unable to test in the final analysis: By focusing on clinically relevant constructs—social cognition, self-organization, and comorbid symptoms—our explorative study aims to identify key factors and explore how ASD and SCH symptom networks differ from neurotypical controls (Borsboom & Cramer, 2013; Robinaugh et al., 2020). Based on prior research (Barneveld et al., 2011; Bora, 2020; Chung et al., 2014; Polner, Faiola, et al., 2021; van Neerven et al., 2021; Velikonja et al., 2019), we hypothesized that mentalization deficits and the disorganized dimension of schizotypy will emerge as the most central nodes within the symptom networks of the diagnostic groups. Difficulties in maintaining a coherent sense of self and distinguishing personal experiences from external reality (Meltzoff, 2007; Brent et al., 2013; Tordjman et al., 2019) would be critical areas for future transdiagnostic research and intervention, we expected more pronounced minimal self perceptual disturbances in ASD and marked significance of schizotypal traits in the schizophrenia group.

### Method S2. Variable selection and data preparation

#### Variable selection

In our analysis, from a spectrum perspective, we have chosen to work with the sum scores of the broad questionnaire material. Given the small number of subjects in clinical samples to be included, variable selection and data preparation was necessary. As there is not yet an explicit consensus on the relationship between the number of variables and sample size in network analysis methodology, guidelines, tutorials, as well as literature applying the methodology were considered (Epskamp & Fried, 2018; Borsboom & Cramer, 2013; Fried & Cramer, 2017; Burger et al.,, 2023). To achieve the highest possible quality of the results, our goal was to ensure that the size of the smallest group was 5 to 10 times the number of the variables to achieve satisfactory and meaningful network stability and accuracy (Epskamp et al., 2018). Ultimately, the SCH group consisted of 62 participants at the end of the multi-round data collection and we aimed to retain between 6 to 12 variables.

First, as the Network Comparison Test cannot handle skip-structure questionnaires, we excluded them. For further qualitative screening, we checked both Cronbach’s alpha and McDonald’s omega reliability indicators. Based on the results of these reliability analyses, the number of variables was further reduced regarding the scales that didn’t reach the satisfactory level, and we also decided to use the total score of the AQ-50 and MZQ as several of their subscales failed to meet the sufficient reliability levels (Table S3 and S4.). Additionally, we decided to merge subscales based on practical and theoretical reasons with the following considerations: we combined the *avoidant*- and *anxious* attachment style subscales of the AAS and labeled it as *insecure attachment style* following related literature (Kara et al., 2023; Schindler, 2019). Following a similar practice, we combined the sum of the *ownership* and *agency* dimensions of self-representation measured by ESSS, referring to it as *minimal self* (Kawano et al., 2021). Minimal self and narrative self are closely related constructs, with the latter building upon the former (Damasio, 1999; Gallagher, 2000), however, we argue that they are distinct (Bortolan, 2020; Newen, 2018; Zahavi, 2010) and should not be collapsed into a single variable.

#### Nonparanormal transformation

Nonparanormal transformation was applied considering the continuous nature of the sum scores (via R package: *huge*; Jiang et al., 2019). Performing this transformation ensures the assumption of multivariate normality which helps to reach a higher quality in the results of the network analysis as it relies on this assumption (Epskamp & Fried, 2018). Beyond this, it also ensures that each node has the same distribution interval to eliminate scaling differences.

Finally, after nonparanormal transformation, in order to complete the variable selection, we also excluded variables one by one based on theoretical and therapeutic considerations. At the end of this procedure, in the light of network stability and accuracy metrics, we decided to keep 10 variables to conduct the network analysis: AQ-50, MSS-B (negative + disorganization subscales), STAI-T (node colour rosa/pink); MZQ, AAS, AAQ-II (node colour light blue); ESSS (minimal + narrative self) node colour green); MSPSS (node colour yellow). Descriptive statistics for all the questionnaires of the original survey that were, and were not included in the final analysis are provided in Table S4. Variance of the variables included in the final analysis are visualized pre- and post-transformation in Figure S1-S4 (pp 12).

# Sample characteristics

### Table S1. Detailed socioeconomic characteristics of the total sample

| **Socioeconomics [*n (%)*]** | | | | | | | | | | | | |
| --- | --- | --- | --- | --- | --- | --- | --- | --- | --- | --- | --- | --- |
| **Education** | elementary school | | vocational school | | high school | | technical school | | undergraduate | | graduate | postgraduate / PhD |
| ASD | 8 (5.13%) | | 6 (3.85%) | | 42 (26.92%) | | 13 (8.33%) | | 53 (33.97%) | | 32 (20.51%) | 2 (1.28%) |
| SCH | 4 (6.45%) | | 2 (3.23%) | | 15 (24.19%) | | 16 (25.81%) | | 11 (17.74%) | | 13 (20.97%) | 1 (1.61%) |
| mNTP | 10 (2.2%) | | 18 (3.96%) | | 127 (27.97%) | | 55 (12.11%) | | 116 (25.55%) | | 111 (24.45%) | 17 (3.74%) |
| NTP | 12 (0.81%) | | 27 (1.82%) | | 191 (12.88%) | | 157 (10.59%) | | 404 (27.24%) | | 567 (38.23%) | 125 (8.43%) |
| **Residence** | capital city | | big city  (100000 – 1M) | | medium size city  (20 000 – 100 000) | | small town  (5000 – 20 000) | | village  (999 – 5000) | | village  (<999) | Farm |
| ASD | 80 (51.28%) | | 23 (14.74%) | | 21 (13.46%) | | 15 (9.62%) | | 12 (7.69%) | | 5 (3.21%) | 0 (0%) |
| SCH | 46 (74.19%) | | 1 (1.61%) | | 4 (6.45%) | | 8 (12.9%) | | 3 (4.84%) | | 0 (0%) | 0 (0%) |
| mNTP | 259 (57.05%) | | 54 (11.89%) | | 61 (13.44%) | | 41 (9.03%) | | 30 (6.61%) | | 9 (1.98%) | 0 (0%) |
| NTP | 852 (57.45%) | | 175 (11.80%) | | 180 (12.14%) | | 145 (9.78%) | | 101 (6.81%) | | 29 (1.96%) | 1 (0.07%) |
| **Occupation** | student | office-based  (full-time) | | office-based  (part-time) | | physical  (full-time) | | physical  (part-time) | | parental leave; caregiving | retired; disability pensioner | unemployed |
| ASD | 42 (26.92%) | 44 (28.21%) | | 22 (14.10%) | | 8 (5.13%) | | 5 (3.21%) | | 8 (5.12%) | 5 (3.21%) | 22 (14.10%) |
| SCH | 8 (12.90%) | 12 (19.35%) | | 5 (8.06%) | | 5 (8.06%) | | 9 (14.52%) | | 0 (0%) | 8 (12.9%) | 15 (24.19%) |
| mNTP | 101 (22.3%) | 237 (52.32%) | | 25 (5.52%) | | 39 (8.61%) | | 7 (1.55%) | | 33 (7.28%) | 1 (0.22%) | 10 (2.21%) |
| NTP | 137 (9.25%) | 965 (65.16%) | | 119 (8.04%) | | 84 (5.67%) | | 15 (1.01%) | | 112 (7.56%) | 14 (0.95%) | 35 (2.36%) |
| **Accommodation** | own property | | family property | | rental flat | | dormitory | | other | | undisclosed |  |
| ASD | 40 (25.64%) | | 84 (53.85%) | | 24 (15.38%) | | 4 (2.56%) | | 0 (0%) | | 4 (2.56%) |  |
| SCH | 17 (27.42%) | | 38 (61.29%) | | 4 (6.45%) | | 0 (0%) | | 2 (3.23%) | | 1 (1.61%) |  |
| mNTP | 167 (36.78%) | | 129 (28.41%) | | 139 (30.62%) | | 13 (2.86%) | | 3 (0.66%) | | 3 (0.66%) |  |
| NTP | 746 (50.30%) | | 357 (24.07%) | | 340 (22.93%) | | 15 (1.01%) | | 17 (1.15%) | | 8 (0.54%) |  |
| **Socioeconomic status** | very good | | above average | | average | | below average | | financial hardship | |  |  |
| ASD | 19 (12.18%) | | 37 (23.72%) | | 68 (43.59%) | | 26 (16.67%) | | 6 (3.85%) | |  |  |
| SCH | 9 (14.52%) | | 18 (29.03%) | | 23 (37.1%) | | 11 (17.74%) | | 1 (1.61%) | |  |  |
| mNTP | 66 (14.57%) | | 200 (44.15%) | | 145 (32.01%) | | 35 (7.73%) | | 7 (1.55%) | |  |  |
| NTP | 244 (16.48%) | | 662 (44.70%) | | 454 (30.65%) | | 105 (7.09%) | | 16 (1.08%) | |  |  |

*Note.* ASD: autism spectrum disorder, SCH: schizophrenia spectrum, NTP: neurotypical participants without psychiatric diagnosis, mNTP: matched neurotypical controls without psychiatric diagnosis

### Table S2. Receipt of psychological support by group

| **Psychological support** [*n (%)*] | | |
| --- | --- | --- |
| **Group** | **Yes** | **No** |
| ASD | 98 (63.64%) | 56 (36.36%) |
| SCH | 59 (96.77%) | 1 (3.23%) |
| mNTP | 103 (23.25%) | 340 (76.75%) |
| NTP | 368 (25.31%) | 1086 (74.69%) |

*Note.* ASD: autism spectrum disorder, SCH: schizophrenia spectrum, NTP: neurotypical participants without psychiatric diagnosis, mNTP: matched neurotypical controls without psychiatric diagnosis. The item on psychological help was optional; consequently, the number of valid responses differs from the total sample size.

# Questionnaires

### Method S3. Description of the instruments included in the final network analysis

#### AQ-50

The Autism-Spectrum Quotient (AQ-50; Baron-Cohen et al., 2001) is a 50-item self-report questionnaire to measure autistic traits. The measurement has screening purposes for adults in the average intelligence range. The items can be grouped into five subscales: Social skill, Attention switching, Attention to details, Communication and Imagination, each of them containing 10 items. The AQ-50 has not yet been validated in Hungarian, but according to the results of foreign studies, the questionnaire proved to have excellent reliability and validity. Similar to these studies, the original version also showed Cronbach's α values ranging between 0.63 and 0.77 (Baron-Cohen et al., 2001). In our study, the five subscales are acceptable (Cronbach’s α=0.49-0.81; McDonald’s ω = 0.53-0.81), while the total scale has excellent internal consistency (Cronbach’s α=88; McDonald’s ω = 0.89).

#### MSS-B

Schizotypal traits were assessed by the Multidimensional Schizotypy Scale – Brief (MSS-B; Gross et al., 2018), which includes three subscales corresponding to the primary symptom domains of schizophrenia: positive, negative, and disorganized. The total score of the questionnaire is not reliably applicable, so separate scores for each subscale are used (Cronbach’s α = 0.8-0.9). The Hungarian adaptation demonstrated satisfactory reliability in a sample of healthy adults (Cronbach’s α = 0.76-0.87) (Polner et al., 2021b). In our study, the subscales of the MSS-B demonstrated good internal consistency in the *positive* (Cronbach’s α=0.78; McDonald’s ω = 0.79), *negative* (Cronbach’s α=0.81; McDonald’s ω = 0.82) and *disorganized* (Cronbach’s α=0.89; McDonald’s ω = 0.89) subscales.

#### STAI-T

Trait anxiety was measured with the 20 items of the State-Trait Anxiety Inventory – Trait Version (STAI-T; Spielberger, 1983). Participants have to rate statements about the physical and psychological characteristics of anxiety on a 4-point Likert-scale. The inventory proved to be a reliable and valid instrument across several studies with excellent reliability values (Cronbach’s α in 0.86–0.95 range) (Stauder és Konkoly-Thege, 2006, Elliot et al., 2001). In our study we found similar internal consistency (Cronbach’s α=0.90; McDonald’s ω = 0.93).

#### ESSS

To examine the subjective aspects for the sense of self, we used the Embodied Sense of Self Scale (ESSS; Asasi et al., 2016). The self-reported scale is built up from 25 items and includes 3 subscales: Ownership with 9 items, Agency with 8 items, and Narrative Self Identity with 8 items. Participants have to rate the statements about experiences corresponding to self-representation on a 5-point Likert scale where 1 means strongly disagree and 5 means strongly agree. Although a Hungarian version has not yet been validated, the subscales of *ownership* (Cronbach’s α=0.81; McDonald’s ω = 0.82), *agency* (Cronbach’s α=0.77; McDonald’s ω = 0.79), and *narrative* (Cronbach’s α=0.78; McDonald’s ω = 0.79) demonstrated satisfactory reliability in our study.

#### MZQ

The Mentalization Questionnaire (MZQ, Hausberg et al., 2012) is a 15-item self-report questionnaire, which was developed to measure mentalization difficulties regarding ’Refusing self-reflection’, ’Emotional awareness’, ’Psychic equivalence mode’, and ’Regulation of affect’. The Hungarian adaptation of the questionnaire was conducted among patients with psychotic disorders with satisfactory reliability values (Cronbach’s α= 0.7-0.9) (Fekete et al., 2019). In our study, the scale demonstrated good internal consistency (Cronbach’s α= 0.86; McDonald’s ω = 0.86).

#### AAS

To measure adult attachment style, the Adult Attachment Scale (AAS, Collins, 1996) has been used. We applied two subscales from the original questionnaire to investigate anxious and avoidant attachment styles as these two proved to be reliable in a Hungarian sample (Cronbach’s α= 0.77-0.87) (Őri et al., 2021). In our study, both subscales demonstrated good reliability (Cronbach’s α= 0.88-0.86; McDonald’s ω = 0.88-0.86).

#### AAQ-II

The AAQ-II (Bond et al., 2011) is a 7-item self-report questionnaire that measures psychological inflexibility, which refers to the tendency to rigidly control reactions to uncomfortable thoughts and emotions, often undermining values-guided actions. The items have to be rated on a 5 point-scale in accordance with how much the participant agrees with the statement. The reliability of the test scores demonstrated strong internal consistency (Cronbach’s α= 0.84) (Bond et al., 2011). Our results showed excellent reliability (Cronbach’s α= 0.93; McDonald’s ω = 0.93).

#### MSPSS

In order to measure the perceived social support during adolescence, we included the Multidimensional Scale of Perceived Social Support (MSPSS, Zimet et al., 1988) with 12 Likert-items in the study. We applied a small modification in the instructions compared to the original questionnaire as we explicitly asked about the perceived adequacy of social support between the ages of 14 and 18. In our study, similarly to the original (Cronbach’s α= 0.88) and the Hungarian validation (Cronbach’s α= 0.91), an excellent reliability value was observed (Cronbach’s α=0.95; McDonald’s ω = 0.95).

## Psychometric properties

### Table S3. Questionnaire reliability: Internal consistency

|  | **Cronbach alpha** | | | **McDonald omega** | | |
| --- | --- | --- | --- | --- | --- | --- |
| **Scale** | **ASD** | **NTP** | **SCH** | **ASD** | **NTP** | **SCH** |
| **AQ-50** | **0.852** | **0.875** | **0.818** | **0.865** | **0.887** | **0.849** |
| AQ social | 0.760 | 0.805 | 0.726 | 0.771 | 0.810 | 0.729 |
| AQ attention switch | 0.643 | 0.702 | 0.656 | 0.652 | 0.705 | 0.662 |
| AQ attention to details | 0.537 | 0.586 | 0.502 | 0.576 | 0.600 | 0.567 |
| AQ communication | 0.706 | 0.710 | 0.534 | 0.714 | 0.722 | 0.532 |
| AQ imagination | 0.613 | 0.581 | 0.495 | 0.618 | 0.608 | 0.604 |
| **MZQ** | **0.811** | **0.856** | **0.830** | **0.817** | **0.857** | **0.834** |
| mzq refuse self refl | 0.407 | 0.518 | 0.365 | 0.452 | 0.546 | 0.389 |
| mzq emotional awareness | 0.697 | 0.777 | 0.714 | 0.716 | 0.781 | 0.727 |
| mzq psychic equivalence | 0.705 | 0.761 | 0.796 | 0.750 | 0.785 | 0.819 |
| mzq affect regulation | 0.552 | 0.564 | 0.538 | 0.592 | 0.609 | 0.636 |
| **AAS (insecure)** | **0.882** | **0.903** | **0.889** | **0.884** | **0.903** | **0.891** |
| aas anxious | 0.862 | 0.880 | 0.898 | 0.865 | 0.883 | 0.898 |
| aas avoidant | 0.832 | 0.863 | 0.830 | 0.835 | 0.864 | 0.840 |
| **STAI-T** | **0.892** | **0.898** | **0.899** | **0.920** | **0.927** | **0.918** |
| **ESSS (narrative)** | **0.733** | **0.782** | **0.855** | **0.744** | **0.787** | **0.860** |
| **ESSS (minimal)** | **0.816** | **0.856** | **0.838** | **0.822** | **0.863** | **0.848** |
| esss ownership | 0.794 | 0.812 | 0.752 | 0.803 | 0.822 | 0.789 |
| esss agency | 0.643 | 0.773 | 0.809 | 0.665 | 0.788 | 0.818 |
| MSS-B (positive) | 0.775 | 0.784 | 0.764 | 0.777 | 0.794 | 0.764 |
| **MSS-B (negative)** | **0.809** | **0.814** | **0.728** | **0.810** | **0.817** | **0.743** |
| **MSS-B (disorganized)** | **0.878** | **0.886** | **0.853** | **0.880** | **0.889** | **0.856** |
| **AAQ-II** | **0.911** | **0.928** | **0.914** | **0.912** | **0.929** | **0.916** |
| **MSPSS** | **0.920** | **0.948** | **0.944** | **0.921** | **0.949** | **0.945** |

*Note.* AQ-50: Autism Spectrum Quotient, MZQ: Mentalization Questionnaire, AAS: Adult Attachment Scale, STAI-T: Spielberger Strait Trait Anxiety Inventory, Trait anxiety, ESSS: Embodied Sense of Self Scale, MSS-B: Multidimensional Schizotypy Scale-Brief, AAQ-2: Acceptance and Action Questionnaire-2, MSPSS: Multidimensional Scale of Perceived Social Support. Scales indicated in bold font were included in the final analysis.

### Table S4. Descriptive and reliability statistics of all study variables by group

| **Variable** | **Group** | **Min** | **Max** | **Mean** | **Median** | **SD** | **Skewness** | **Kurtosis** | **Cronbach alpha** | **McDonald omega** |
| --- | --- | --- | --- | --- | --- | --- | --- | --- | --- | --- |
| Autistic traits  Social skills | ASD | 0 | 10 | 6.76 | 7.5 | 2.54 | -0.685 | -0.437 | 0.75 | 0.79 |
|  | SCH | 0 | 10 | 4.05 | 3.5 | 2.58 | 0.321 | -0.953 | 0.73 | 0.76 |
|  | mNTP | 0 | 10 | 4.18 | 4 | 3.03 | 0.262 | -1.145 | 0.82 | 0.85 |
| Autistic traits Attention switching | ASD | 2 | 10 | 7.88 | 8 | 1.95 | -0.942 | 0.403 | 0.62 | 0.67 |
|  | SCH | 0 | 10 | 5.89 | 6 | 2.37 | -0.290 | -0.642 | 0.65 | 0.70 |
|  | mNTP | 0 | 10 | 5.43 | 5 | 2.54 | -0.050 | -0.815 | 0.69 | 0.73 |
| Autistic traits Attention to details | ASD | 2 | 10 | 6.26 | 6 | 1.97 | 0.079 | -0.606 | 0.54 | 0.61 |
|  | SCH | 0 | 9 | 4.6 | 4 | 2.1 | 0.227 | -0.233 | 0.53 | 0.63 |
|  | mNTP | 0 | 10 | 5.41 | 5 | 2.08 | -0.023 | -0.570 | 0.52 | 0.62 |
| Autistic traits Communication | ASD | 0 | 10 | 6.39 | 7 | 2.46 | -0.327 | -0.818 | 0.71 | 0.76 |
|  | SCH | 0 | 9 | 3.66 | 4 | 2.02 | 0.506 | 0.117 | 0.50 | 0.64 |
|  | mNTP | 0 | 10 | 3.58 | 3 | 2.49 | 0.452 | -0.721 | 0.73 | 0.78 |
| Autistic traits Imagination | ASD | 1 | 9 | 4.96 | 5 | 2.19 | 0.123 | -0.837 | 0.60 | 0.65 |
|  | SCH | 0 | 9 | 4.47 | 4.5 | 2.02 | 0.005 | -0.442 | 0.59 | 0.64 |
|  | mNTP | 0 | 10 | 3.41 | 3 | 2.09 | 0.540 | -0.001 | 0.62 | 0.65 |
| **Autistic trait (AQ)**  **Total score** | ASD | 12 | 49 | 32.24 | 33.5 | 7.63 | -0.521 | -0.386 | 0.85 | 0.86 |
|  | SCH | 6 | 44 | 22.66 | 22 | 7.57 | 0.285 | -0.241 | 0.85 | 0.86 |
|  | mNTP | 4 | 48 | 22.01 | 21 | 8.84 | 0.357 | -0.507 | 0.89 | 0.90 |
| Mentalization  Refusing self-reflection | ASD | 0 | 16 | 8.11 | 8 | 3.29 | -0.064 | -0468 | 0.43 | 0.53 |
|  | SCH | 1 | 16 | 5.79 | 6 | 2.81 | 0.711 | 1.208 | 0.37 | 0.45 |
|  | mNTP | 0 | 15 | 6.69 | 7 | 3.22 | 0.099 | -0.529 | 0.51 | 0.59 |
| Mentalization  Emotional awareness | ASD | 0 | 16 | 10.47 | 11 | 3.88 | -0.627 | -0.358 | 0.72 | 0.77 |
|  | SCH | 0 | 16 | 7.21 | 8 | 3.77 | 0.053 | -0.346 | 0.71 | 0.73 |
|  | mNTP | 0 | 16 | 7.84 | 8 | 4.06 | 0.027 | -0.940 | 0.78 | 0.82 |
| Mentalization  Psychic equivalence | ASD | 0 | 16 | 11.13 | 12 | 3.84 | -0.69 | -0.401 | 0.71 | 0.82 |
|  | SCH | 0 | 16 | 8.16 | 9 | 4.3 | -0.432 | -0.738 | 0.79 | 0.84 |
|  | mNTP | 0 | 16 | 8.36 | 8 | 4.04 | -0.113 | -0.746 | 0.73 | 0.81 |
| Mentalization  Regulation of affect | ASD | 0 | 12 | 6.81 | 7 | 2.86 | -0.206 | -0.646 | 0.55 | 0.60 |
|  | SCH | 0 | 12 | 4.97 | 5 | 2.91 | 0.238 | -0.531 | 0.53 | 0.64 |
|  | mNTP | 0 | 12 | 4.89 | 5 | 2.64 | 0.163 | -0.646 | 0.49 | 0.55 |
| **Mentalization (MZQ)**  **Total score** | ASD | 13 | 60 | 36.53 | 38 | 10.33 | -0.169 | -0.876 | 0.81 | 0.83 |
|  | SCH | 6 | 56 | 26.13 | 27 | 10.58 | 0.084 | -0.115 | 0.82 | 0.86 |
|  | mNTP | 2 | 54 | 27.77 | 29 | 10.83 | -0.067 | -0.462 | 0.84 | 0.86 |
| Attachment style  anxious | ASD | 6 | 30 | 20.59 | 21 | 6.4 | -0.346 | -0.827 | 0.87 | 0.90 |
|  | SCH | 6 | 29 | 16.35 | 16 | 6.77 | 0.105 | -1.140 | 0.9 | 0.93 |
|  | mNTP | 6 | 30 | 16.8 | 16.5 | 6.6 | 0.172 | -0.957 | 0.88 | 0.91 |
| Attachment style  avoidant | ASD | 18 | 60 | 40.67 | 41 | 9.75 | -0.216 | -0.699 | 0.84 | 0.87 |
|  | SCH | 17 | 59 | 34.39 | 34 | 8.84 | 0.547 | 0.305 | 0.83 | 0.86 |
|  | mNTP | 15 | 60 | 35.83 | 36 | 9.48 | -0.006 | -0.789 | 0.86 | .088 |
| **Attachment style (AAS)**  **insecure** | ASD | 30 | 90 | 61.26 | 62 | 14.24 | -0.241 | -0.746 | 0.89 | 0.91 |
|  | SCH | 24 | 84 | 50.74 | 50 | 13.71 | 0.173 | -0.594 | 0.89 | 0.91 |
|  | mNTP | 22 | 90 | 52.63 | 53 | 14.38 | -0.039 | -0.810 | 0.9 | 0.92 |
| Depression (BDI) | ASD | 0 | 50 | 16.24 | 14 | 11.46 | 0.739 | 0.098 | 0.91 | 0.92 |
|  | SCH | 0 | 32 | 12.18 | 11 | 8.71 | 0.445 | -0.976 | 0.87 | 0.89 |
|  | mNTP | 0 | 47 | 12.13 | 10 | 9.15 | 0.989 | 0.787 | 0.89 | 0.90 |
| ADHD self-report ASRS, A | ASD | 0 | 6 | 3.75 | 4 | 1.53 | -0.318 | -0.649 | 0.53 | 0.61 |
|  | SCH | 0 | 6 | 3.02 | 3 | 1.65 | -0.495 | -0.813 | 0.6 | 0.71 |
|  | mNTP | 0 | 6 | 2.76 | 3 | 1.7 | -0.009 | -0.974 | 0.62 | 0.68 |
| ADHD self-report ASRS, B | ASD | 0 | 12 | 6.17 | 6 | 2.97 | 0.043 | -1.039 | 0.75 | 0.77 |
|  | SCH | 0 | 12 | 4.06 | 4 | 2.83 | 0.391 | -0.451 | 0.74 | 0.77 |
|  | mNTP | 0 | 12 | 4.32 | 4 | 3 | 0.496 | -0.516 | 0.78 | 0.79 |
| **Trait anxiety (STAI-T)** | ASD | 25 | 77 | 53.19 | 54 | 11.31 | -0.210 | -0.600 | 0.92 | 0.93 |
|  | SCH | 26 | 71 | 46.82 | 46.5 | 10.30 | 0.170 | -0.820 | 0.92 | 0.93 |
|  | mNTP | 25 | 77 | 47.26 | 47 | 10.64 | 0.190 | -0.580 | 0.92 | 0.93 |
| Cognitive insight (BCIS) self-reflection | ASD | 18 | 34 | 25.65 | 26 | 3.13 | 0.004 | 0.176 | 0.51 | 0.52 |
|  | SCH | 15 | 32 | 25 | 25 | 3.45 | -0.391 | 0.104 | 0.59 | 0.68 |
|  | mNTP | 12 | 34 | 23.43 | 23 | 3.61 | 0.043 | -0.091 | 0.64 | 0.69 |
| Cognitive insight self-certainty | ASD | 9 | 22 | 15.29 | 15 | 3.01 | 0.069 | -0.791 | 0.65 | 0.72 |
|  | SCH | 6 | 20 | 14.7 | 15 | 2.61 | -0.393 | 0.492 | 0.64 | 0.75 |
|  | mNTP | 8 | 24 | 15.95 | 16 | 2.57 | -0.017 | 0.341 | 0.62 | 0.63 |
| Reflecting functioning (RFQ) certainty | ASD | 0 | 14 | 3.81 | 3 | 3.79 | 0.841 | -0.446 | 0.69 | 0.78 |
|  | SCH | 0 | 18 | 6.94 | 6 | 5.19 | 0.349 | -1.101 | 0.81 | 0.85 |
|  | mNTP | 0 | 18 | 6.62 | 6 | 5.13 | 0.344 | -1.043 | 0.83 | 0.88 |
| Reflecting functioning (RFQ) uncertainty | ASD | 0 | 18 | 7.14 | 7 | 4.28 | 0.233 | -0.509 | 0.68 | 0.79 |
|  | SCH | 0 | 15 | 3.98 | 3 | 3.82 | 0.953 | -0.085 | 0.73 | 0.80 |
|  | mNTP | 0 | 18 | 7.14 | 7 | 4.28 | 0.233 | -0.509 | 0.78 | 0.82 |
| Self-ownership (ESSS) | ASD | 9 | 45 | 22.92 | 23 | 8.09 | 0.34 | -0.46 | 0.79 | 0.80 |
|  | SCH | 9 | 34 | 17.31 | 17 | 6.52 | 0.55 | -0.63 | 0.75 | 0.79 |
|  | mNTP | 9 | 39 | 18.21 | 16 | 7.34 | 0.66 | -0.57 | 0.81 | 0.82 |
| Self-agency (ESSS) | ASD | 9 | 40 | 22.1 | 22 | 6.07 | 0.14 | -0.56 | 0.64 | 0.66 |
|  | SCH | 8 | 32 | 16.71 | 14.5 | 6.31 | 0.49 | -0.94 | 0.81 | 0.82 |
|  | mNTP | 8 | 36 | 18.43 | 17 | 6.41 | 0.6 | -0.31 | 0.77 | 0.79 |
| **Minimal self (ESSS)** | ASD | 21 | 77 | 45.02 | 44 | 12.34 | 0.33 | -0.44 | 0.82 | 0.82 |
|  | SCH | 17 | 61 | 34.02 | 32 | 11.04 | 0.43 | -0.76 | 0.84 | 0.85 |
|  | mNTP | 17 | 73 | 36.64 | 34 | 12.2 | 0.61 | -0.41 | 0.86 | 0.86 |
| **Narrative-self (ESSS)** | ASD | 10 | 40 | 28.58 | 29 | 6.33 | -0.45 | 0.1 | 0.73 | 0.74 |
|  | SCH | 10 | 38 | 24.21 | 23.5 | 7.81 | 0.04 | -1.16 | 0.86 | 0.86 |
|  | mNTP | 9 | 40 | 25.57 | 26 | 6.57 | -0.01 | -0.68 | 0.78 | 0.79 |
| Schizotypy (MSS-B)  Positive schizotypy | ASD | 0 | 9 | 2.89 | 2 | 2.74 | 0.703 | -0.682 | 0.77 | 0.79 |
|  | SCH | 0 | 10 | 3.03 | 2 | 2.74 | 0.682 | -0.656 | 0.76 | 0.80 |
|  | mNTP | 0 | 13 | 2.2 | 1 | 2.55 | 1.568 | 2.404 | 0.80 | 0.81 |
| **Schizotypy (MSS-B)**  **Negative schizotypy** | ASD | 0 | 12 | 4.91 | 5 | 3.32 | 0.269 | -1.064 | 0.81 | 0.83 |
|  | SCH | 0 | 11 | 3.21 | 2.5 | 2.66 | 0.899 | 0.075 | 0.73 | 0.77 |
|  | mNTP | 0 | 13 | 3.92 | 3 | 3.31 | 0.704 | -0.552 | 0.83 | 0.85 |
| **Schizotypy (MSS-B)**  **Disorganized schizotypy** | ASD | 0 | 12 | 5.33 | 5 | 3.77 | 0.241 | -1.142 | 0.88 | 0.89 |
|  | SCH | 0 | 11 | 3.11 | 2 | 3.2 | 0.863 | -0.578 | 0.85 | 0.87 |
|  | mNTP | 0 | 12 | 2.76 | 1 | 3.31 | 1.157 | 0.290 | 0.89 | 0.90 |
| **Perceived social support (MSPSS)** | ASD | 12 | 60 | 35.99 | 37 | 11.39 | -0.072 | -0.631 | 0.92 | 0.95 |
|  | SCH | 12 | 60 | 42.89 | 44.5 | 12.33 | -0.486 | -0.599 | 0.94 | 0.96 |
|  | mNTP | 12 | 60 | 41.46 | 43 | 11.43 | -0.481 | -0.487 | 0.94 | 0.96 |
| Big Five (BFAS-I) Intellect | ASD | 11 | 50 | 34.21 | 36 | 8.11 | -0.617 | -0.006 | 0.73 | 0.77 |
|  | SCH | 20 | 50 | 32.82 | 33 | 6.88 | 0.152 | -0.624 | 0.73 | 0.79 |
|  | mNTP | 14 | 50 | 37.36 | 37 | 6.56 | -0.441 | 0.097 | 0.81 | 0.84 |
| Big Five (BFAS-O) Openness | ASD | 20 | 50 | 37.47 | 38 | 6.46 | -0.273 | -0.326 | 0.84 | 0.87 |
|  | SCH | 20 | 49 | 36.21 | 36.5 | 6.32 | -0.122 | -0.219 | 0.81 | 0.85 |
|  | mNTP | 14 | 50 | 37.5 | 38 | 6.61 | -0.423 | 0.074 | 0.77 | 0.80 |
| **Psychological inflexibility (AAQ-II)** | ASD | 7 | 49 | 31.71 | 32 | 10.09 | -0.315 | -0.637 | 0.91 | 0.93 |
|  | SCH | 7 | 46 | 23.27 | 22 | 9.99 | 0.263 | -1.066 | 0.91 | 0.93 |
|  | mNTP | 7 | 49 | 23.98 | 24 | 10.48 | 0.312 | -0.697 | 0.93 | 0.94 |

*Note.* ASD: autism spectrum disorder, SCH: schizophrenia spectrum, NTP: neurotypical participants without psychiatric diagnosis, mNTP: matched neurotypical controls without psychiatric diagnosis. Autism Spectrum Quotient, MZQ: Mentalization Questionnaire, AAS: Adult Attachment Scale, BDI: Beck Depression Inventory, STAI-T: Spielberger Strait Trait Anxiety Inventory, Trait anxiety, ASRS: ADHD Self Report Scale, BCIS: Beck Cognitive Insight Scale, RFQ: Reflective Functioning Questionnaire, ESSS: Embodied Sense of Self Scale, MSS-B: Multidimensional Schizotypy Scale-Brief, MSPSS: Multidimensional Scale of Perceived Social Support, BFAS-I,-O: Big Five Aspects Scale, Intellect, Openness, AAQ-2: Acceptance and Action Questionnaire-2. Questionnaires and subscales indicated in bold font were included in the final analysis

## Non-paranormal transformation

### Figure S1. Distribution of questionnaire scores in the mNTP sample - pre- and post-transformation

###
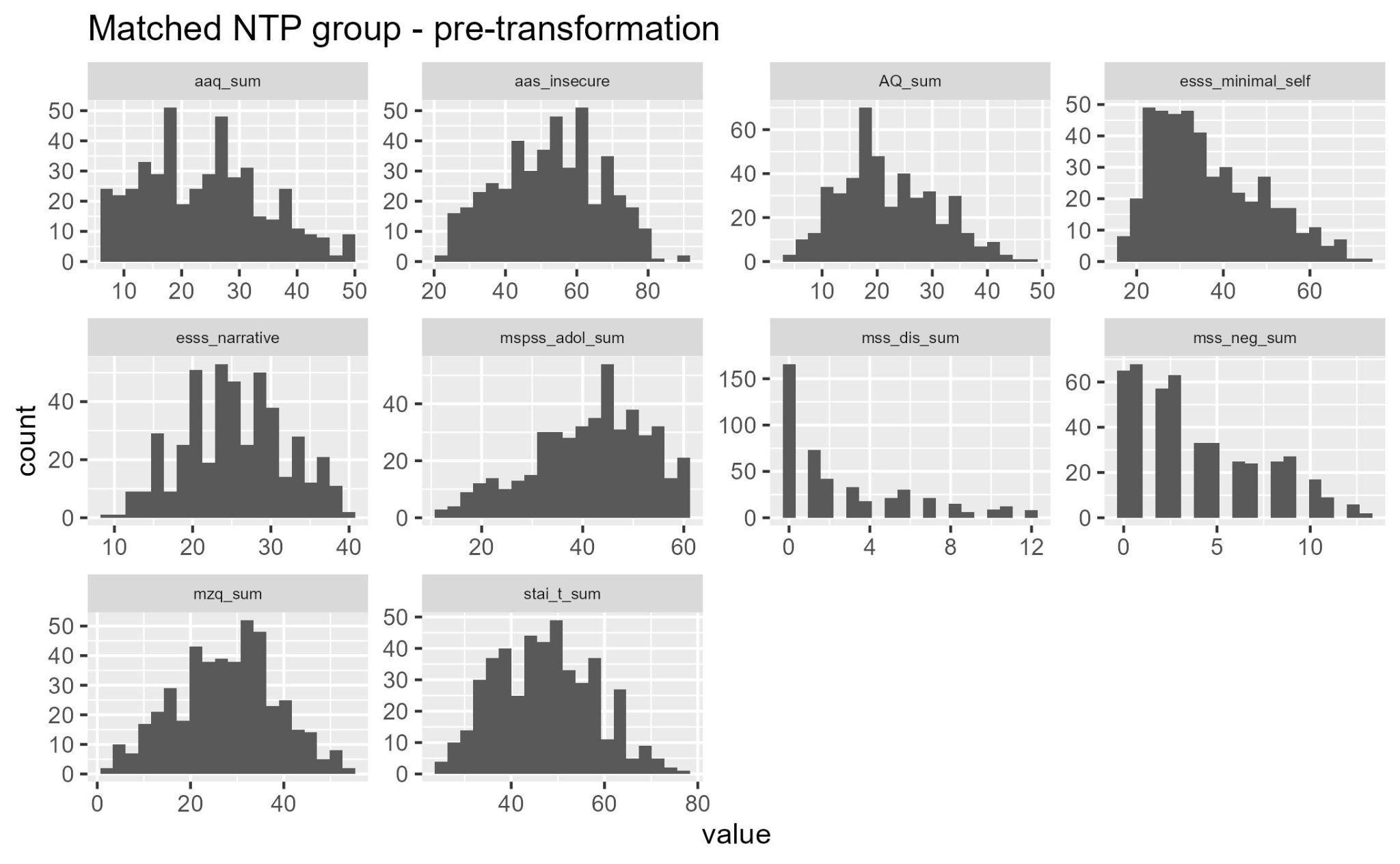


###
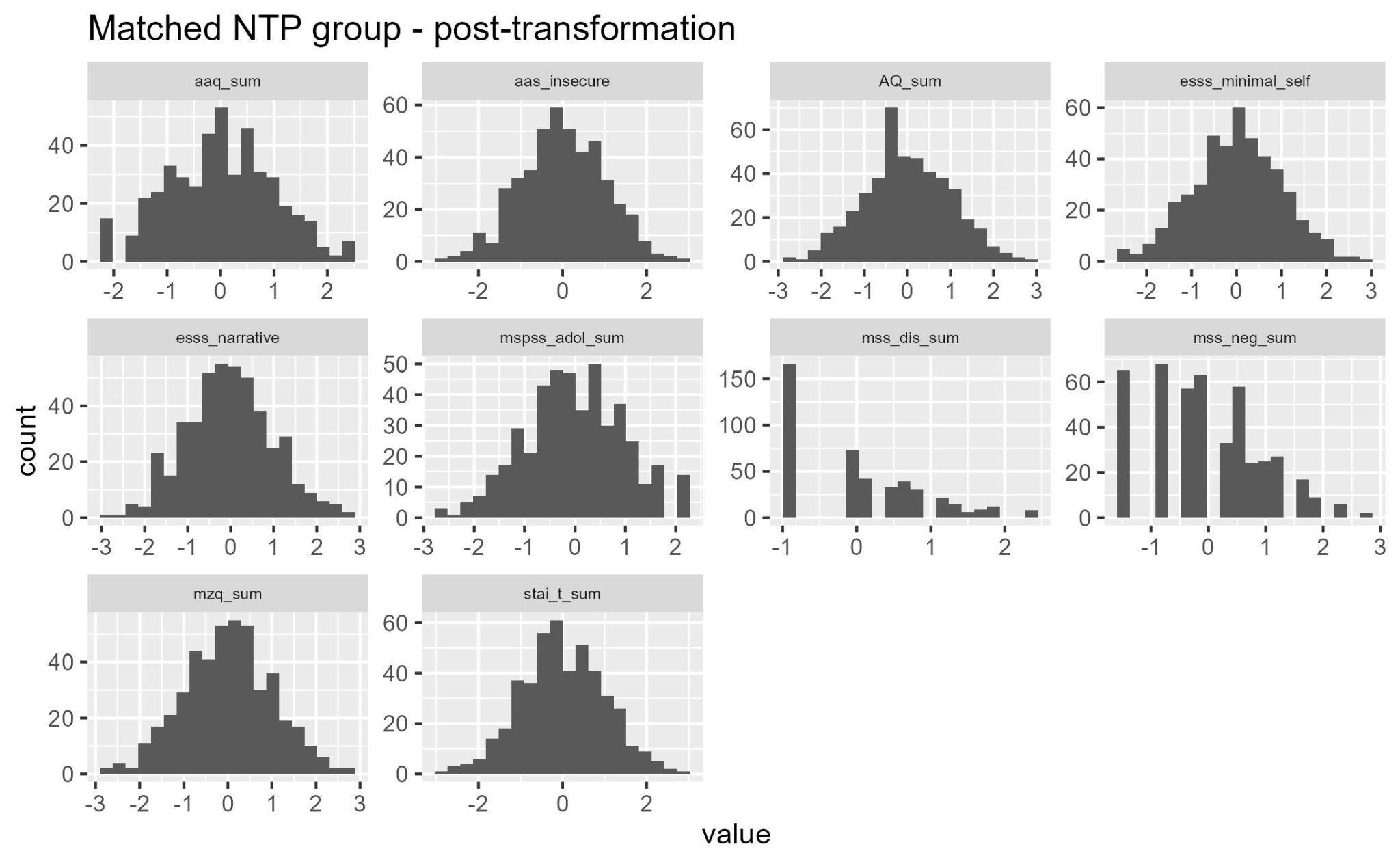


###

### Figure S2. Distribution of questionnaire scores in the ASD sample - pre- and post-transformation


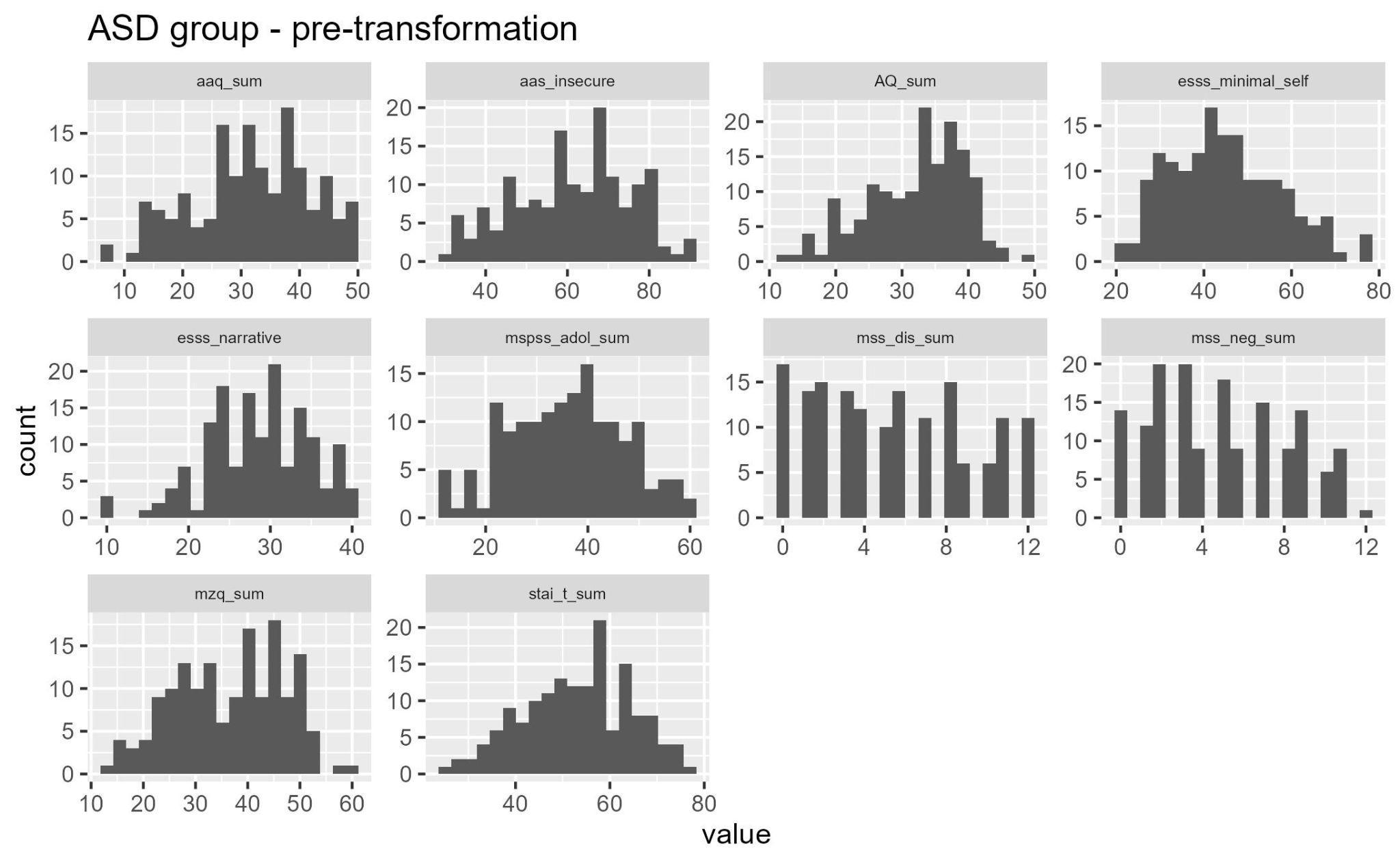

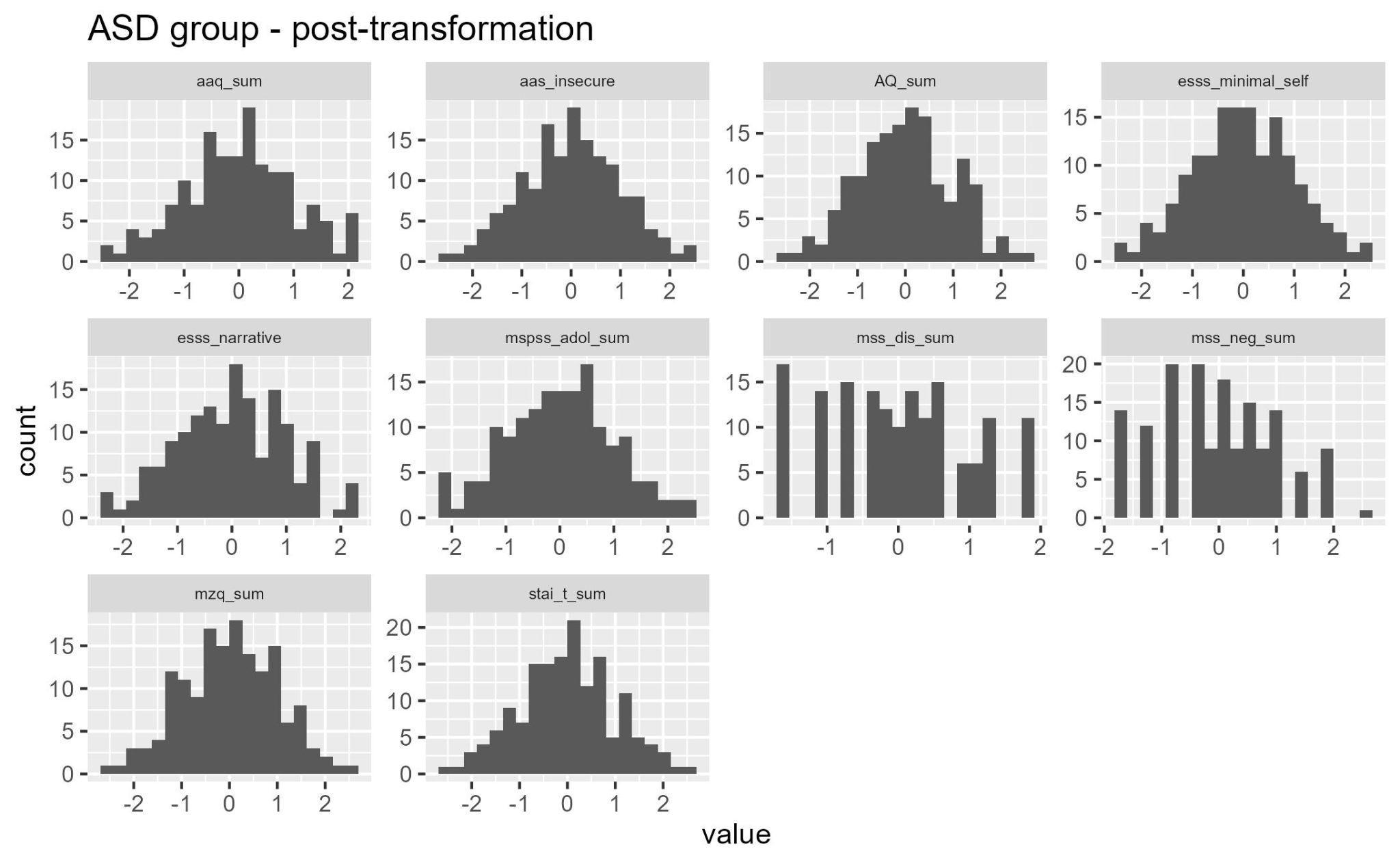


#

#

###

### Figure S3. Distribution of questionnaire scores in the SCH sample - pre- and post-transformation


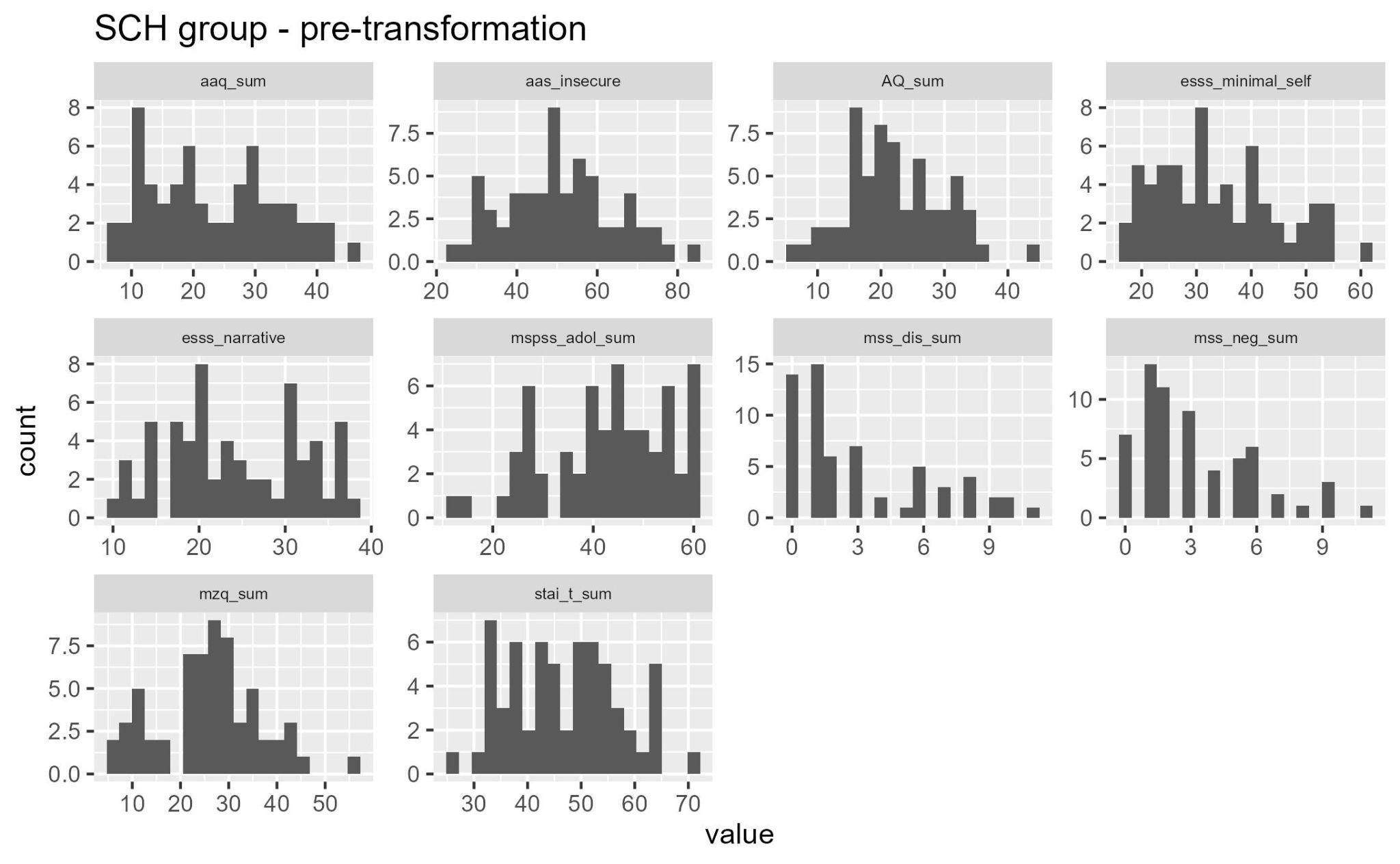

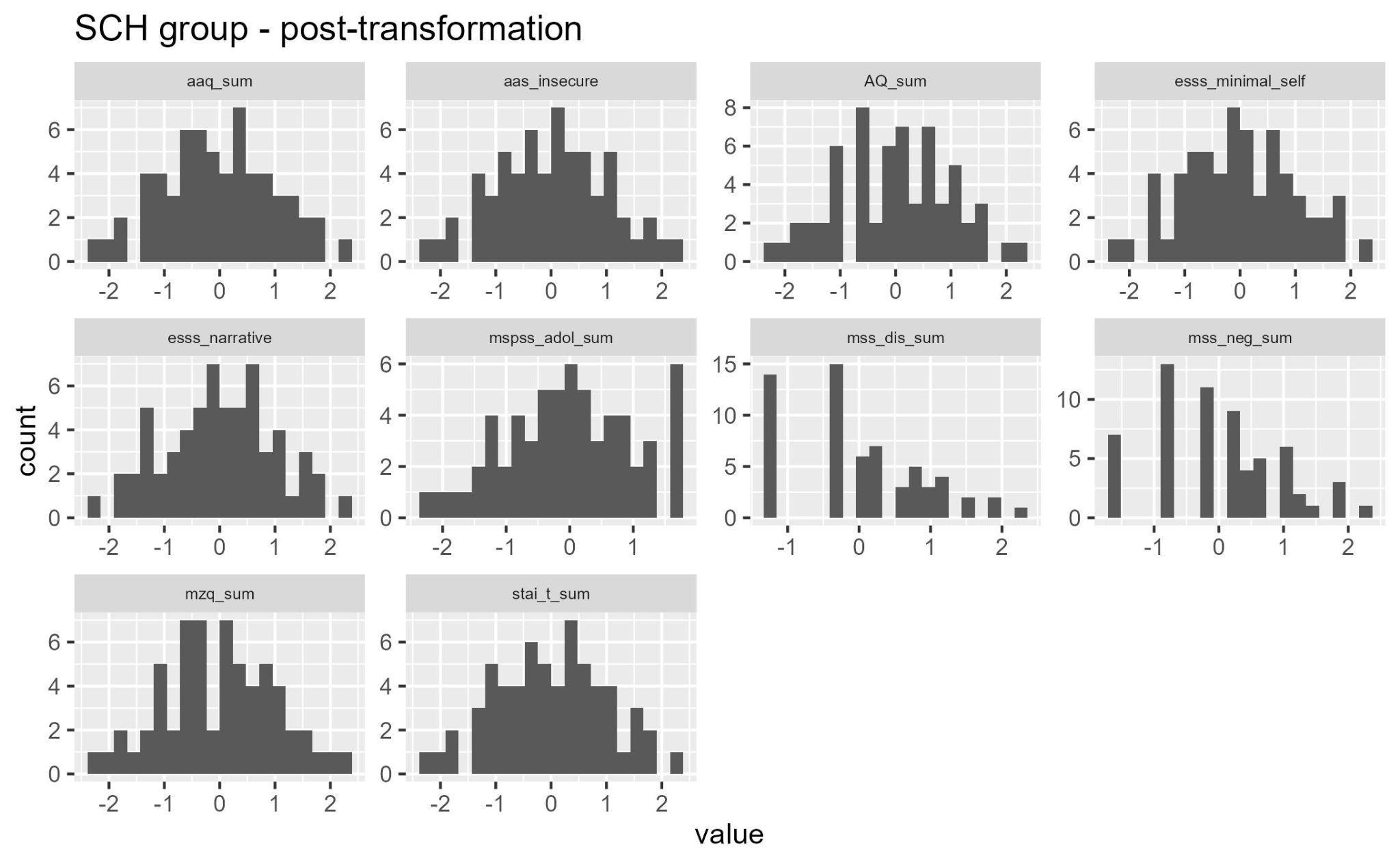


### Figure S4. Distribution of questionnaire scores in the NTP sample - pre- and post-transformation

###
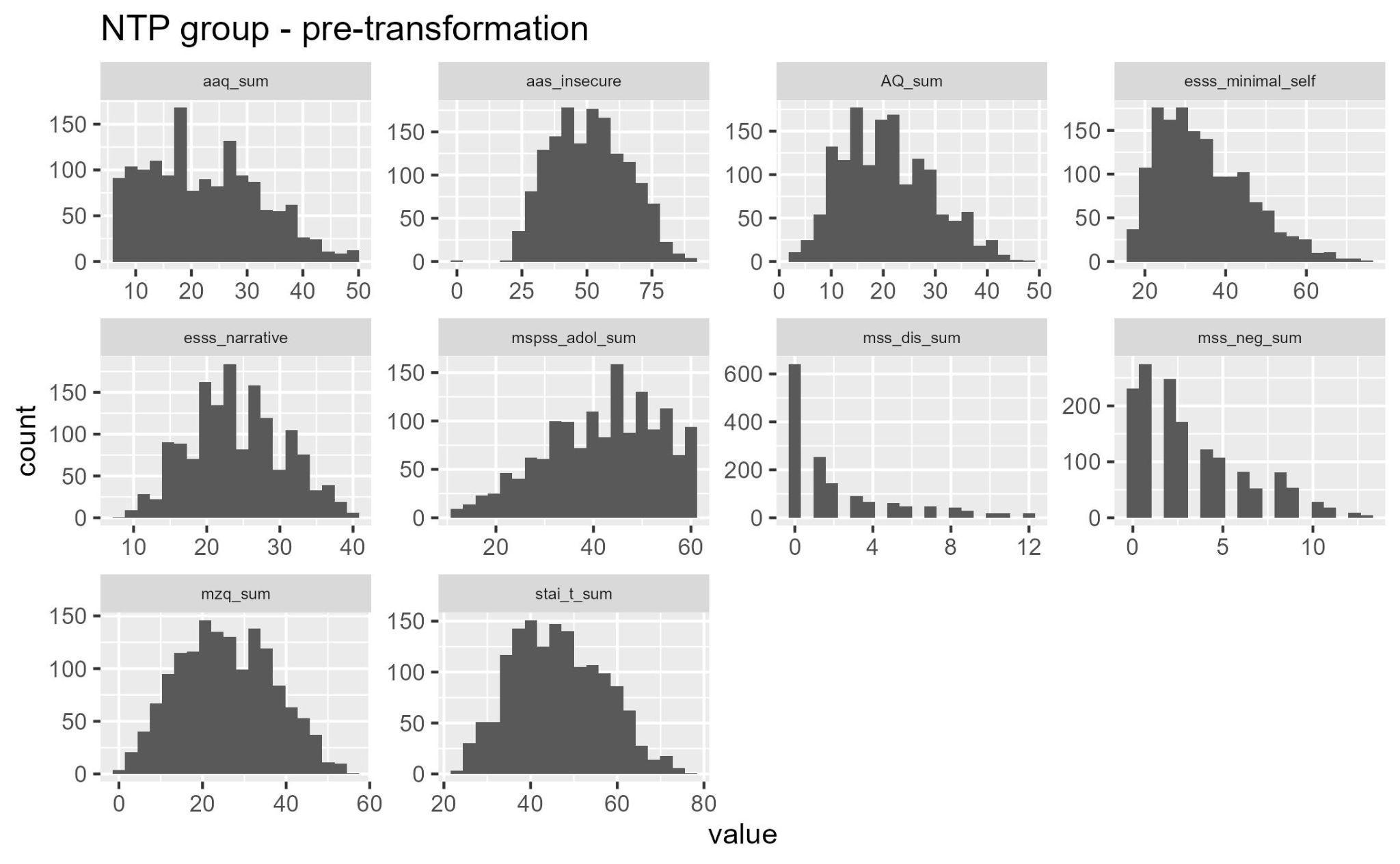

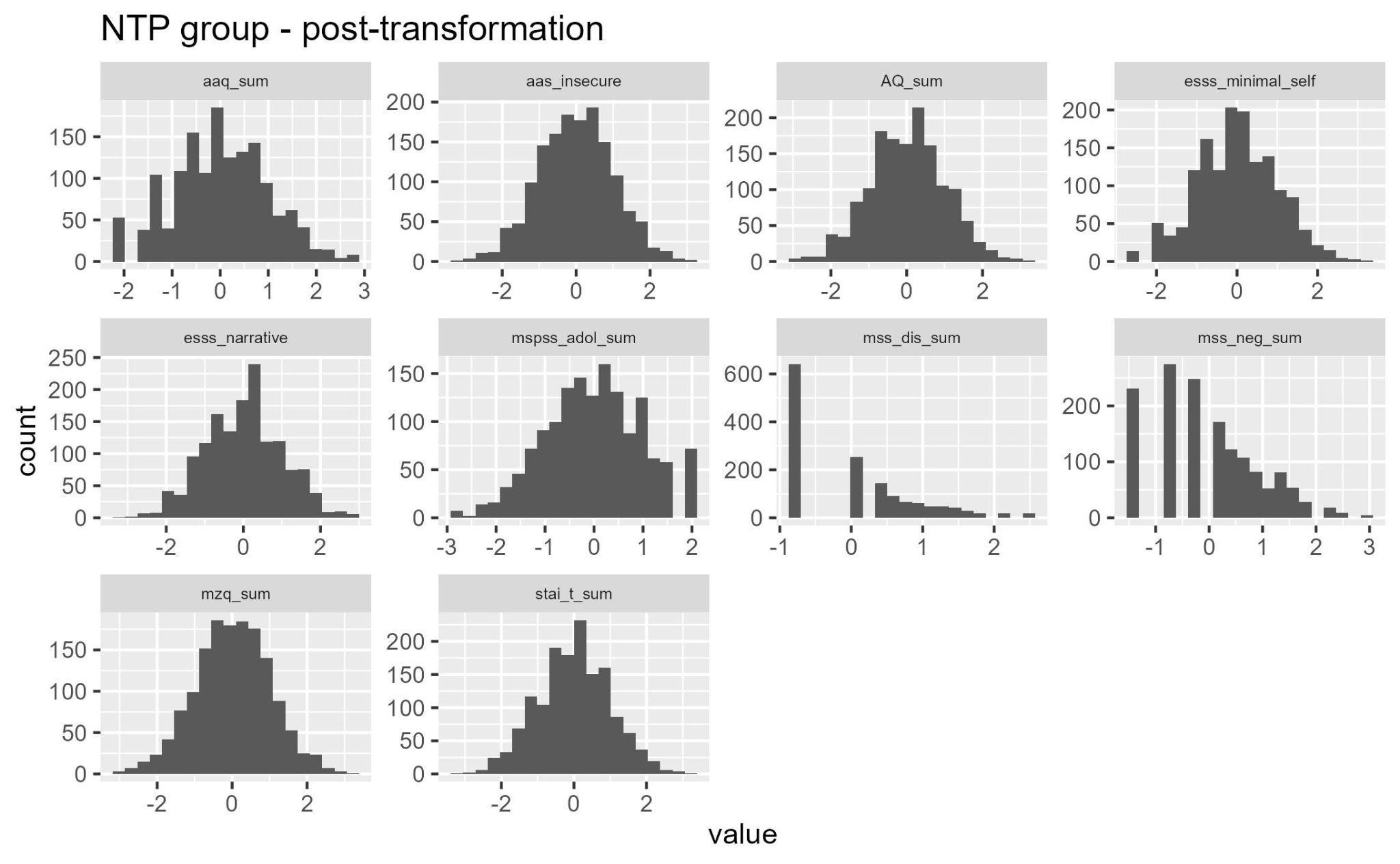


###

# Network analysis

### Table S5. Edge weights across groups

| **Node 1** | **Node 2** | **ASD** | **mNTP** | **SCH** |
| --- | --- | --- | --- | --- |
| AQ_sum | mzq_sum | 0.194 | 0.170 | 0.217 |
| aas_insecure | mzq_sum | 0.230 | 0.325 | 0.180 |
| esss_minimal_self | mzq_sum | 0.180 | 0.018 | 0.000 |
| esss_narrative | mzq_sum | 0.077 | 0.214 | 0.185 |
| mss_neg_sum | mzq_sum | 0.105 | 0.067 | 0.000 |
| mss_dis_sum | mzq_sum | 0.015 | 0.071 | 0.000 |
| aaq_sum | mzq_sum | 0.087 | 0.103 | 0.096 |
| mspss_adol_sum | mzq_sum | -0.078 | 0.000 | 0.000 |
| aaq_sum | aas_insecure | 0.210 | 0.101 | 0.283 |
| AQ_sum | esss_minimal_self | 0.077 | 0.160 | 0.000 |
| aas_insecure | esss_minimal_self | 0.000 | 0.005 | 0.098 |
| aaq_sum | esss_minimal_self | 0.007 | 0.000 | 0.000 |
| AQ_sum | esss_narrative | 0.000 | 0.000 | 0.000 |
| aas_insecure | esss_narrative | 0.059 | 0.000 | 0.050 |
| esss_minimal_self | esss_narrative | 0.261 | 0.424 | 0.363 |
| aaq_sum | esss_narrative | 0.095 | 0.049 | 0.192 |
| AQ_sum | mss_neg_sum | 0.192 | 0.364 | 0.305 |
| aas_insecure | mss_neg_sum | 0.172 | 0.209 | 0.000 |
| esss_minimal_self | mss_neg_sum | 0.000 | 0.000 | 0.000 |
| esss_narrative | mss_neg_sum | 0.014 | 0.000 | 0.000 |
| mss_dis_sum | mss_neg_sum | 0.031 | 0.045 | 0.024 |
| aaq_sum | mss_neg_sum | 0.000 | -0.015 | 0.000 |
| mspss_adol_sum | mss_neg_sum | -0.109 | -0.116 | -0.245 |
| AQ_sum | mss_dis_sum | 0.000 | 0.130 | 0.242 |
| aas_insecure | mss_dis_sum | 0.000 | 0.000 | 0.016 |
| esss_minimal_self | mss_dis_sum | 0.224 | 0.177 | 0.141 |
| esss_narrative | mss_dis_sum | 0.222 | 0.194 | 0.053 |
| aaq_sum | mss_dis_sum | 0.050 | 0.198 | 0.225 |
| mspss_adol_sum | mss_dis_sum | 0.000 | 0.000 | 0.000 |
| AQ_sum | stai_t_sum | 0.005 | 0.000 | 0.000 |
| mzq_sum | stai_t_sum | 0.194 | 0.178 | 0.227 |
| aas_insecure | stai_t_sum | 0.149 | 0.159 | 0.000 |
| esss_minimal_self | stai_t_sum | 0.028 | 0.000 | 0.243 |
| esss_narrative | stai_t_sum | 0.132 | 0.124 | 0.041 |
| mss_neg_sum | stai_t_sum | 0.000 | -0.152 | -0.104 |
| mss_dis_sum | stai_t_sum | 0.067 | 0.019 | 0.112 |
| aaq_sum | stai_t_sum | 0.512 | 0.493 | 0.270 |
| mspss_adol_sum | stai_t_sum | 0.000 | 0.000 | 0.000 |
| AQ_sum | mspss_adol_sum | -0.071 | -0.084 | 0.000 |
| aas_insecure | mspss_adol_sum | -0.103 | -0.248 | -0.281 |
| esss_minimal_self | mspss_adol_sum | 0.000 | -0.043 | -0.021 |
| esss_narrative | mspss_adol_sum | -0.067 | 0.000 | -0.181 |
| aaq_sum | mspss_adol_sum | 0.000 | -0.044 | 0.000 |
| aas_insecure | AQ_sum | 0.000 | 0.091 | 0.093 |
| aaq_sum | AQ_sum | 0.000 | 0.000 | 0.000 |

### Figure S5. Correlation matrix of variables in the matched NTP group


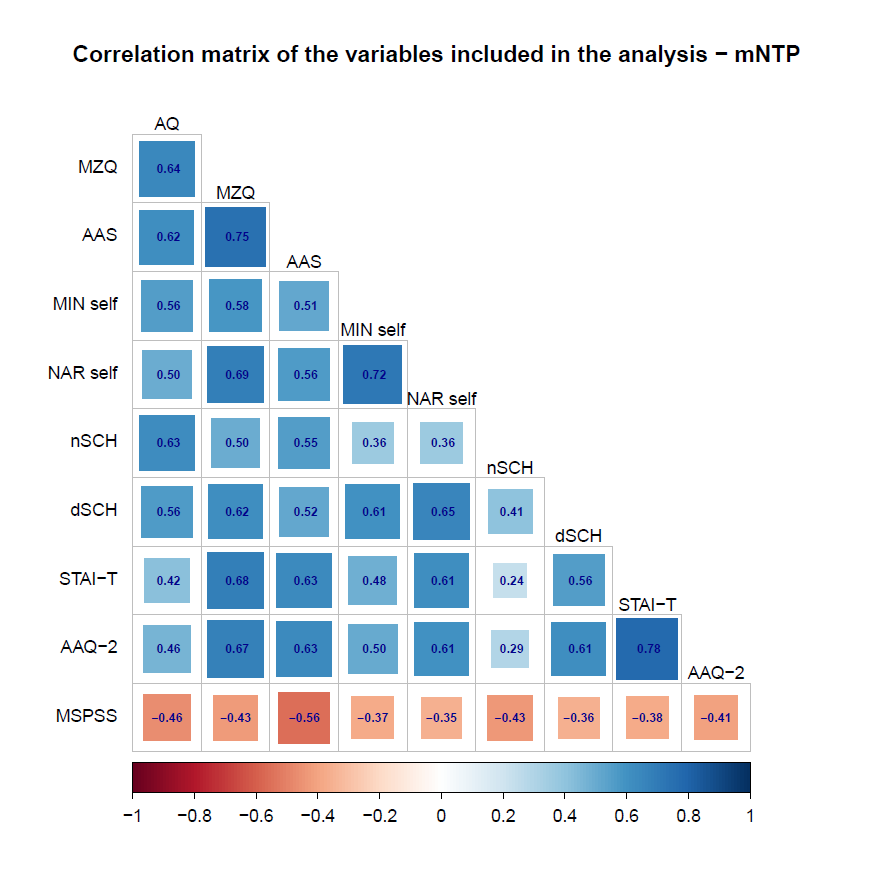


### Figure S6. Correlation matrix of variables in the ASD group


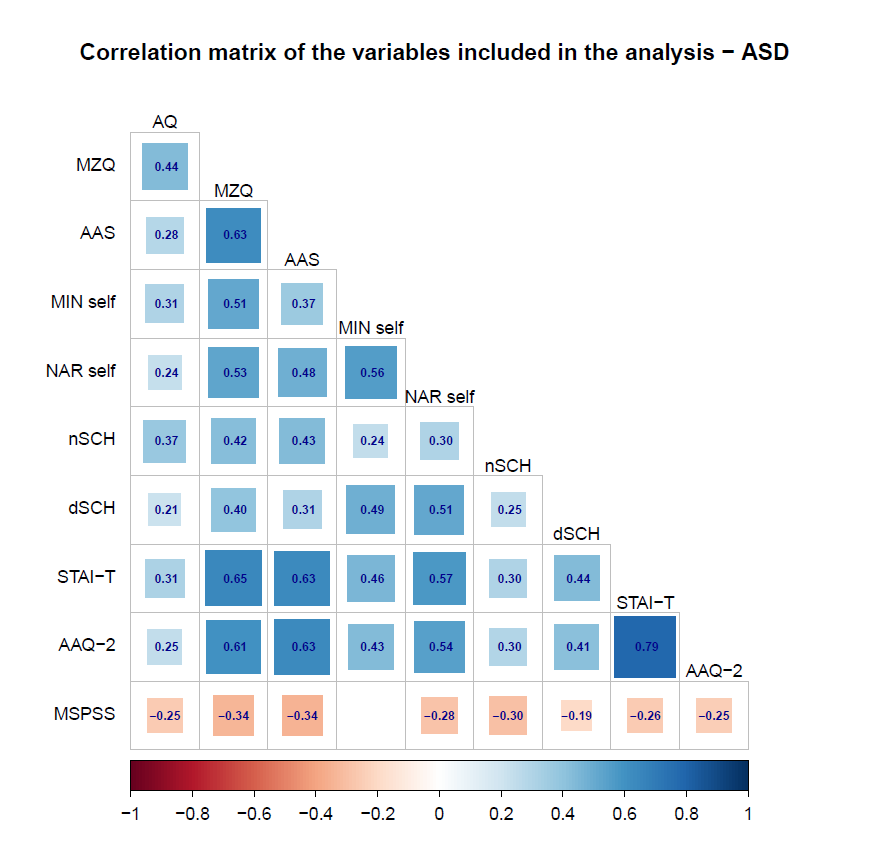


### Figure S7. Correlation matrix of variables in the SCH group


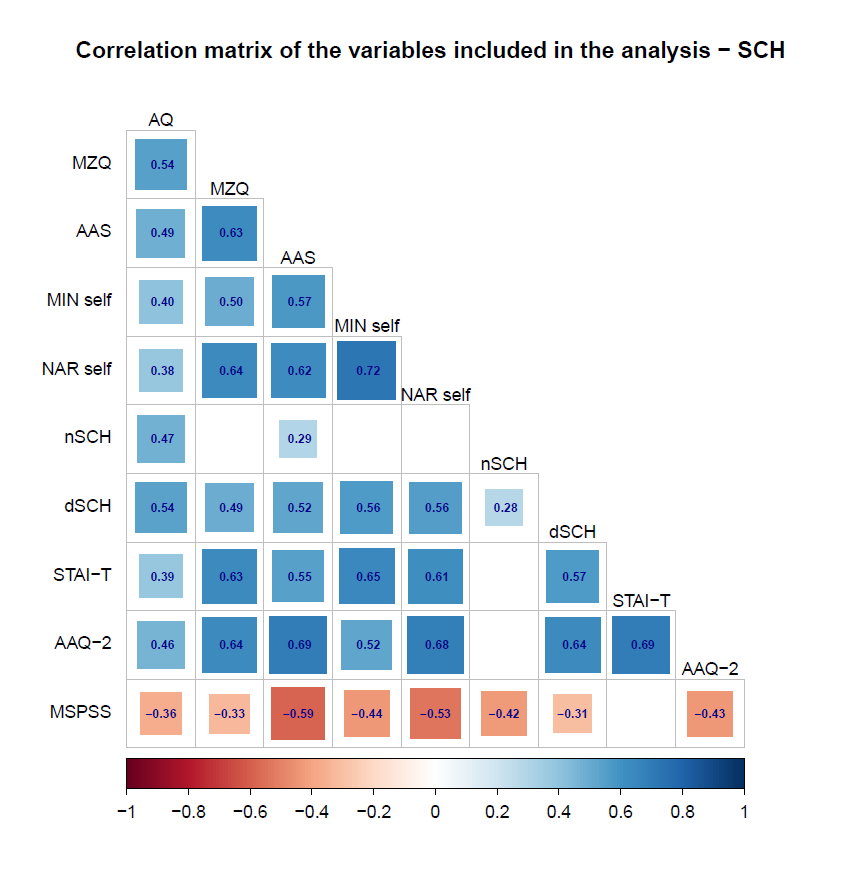


##

## Node predictability

### Table S6. Node predictability values

| **Node** | **ASD** | **mNTP** | **SCH** |
| --- | --- | --- | --- |
| AQ | 0.192 | 0.591 | 0.416 |
| MZQ | 0.554 | 0.702 | 0.585 |
| AAS | 0.496 | 0.682 | 0.603 |
| MIN self | 0.387 | 0.569 | 0.465 |
| NAR self | 0.459 | 0.660 | 0.693 |
| nSCH | 0.260 | 0.434 | 0.259 |
| dSCH | 0.328 | 0.542 | 0.491 |
| STAI-T | 0.667 | 0.657 | 0.591 |
| AAQ-2 | 0.595 | 0.667 | 0.677 |
| MSPSS | 0 | 0.347 | 0.444 |

## Centrality metrics

### Table S7. Node centrality metrics (strength, expected influence, closeness, and betweenness) across diagnostic groups

| **Group** | **Node** | **Strength** | **Expected influence** | **Closeness** | **Betweenness** | **Strength**  **z-score** | **Expected influence**  **z-score** | **Closeness**  **z-score** | **Betweenness**  **z-score** |
| --- | --- | --- | --- | --- | --- | --- | --- | --- | --- |
| **ASD** | AQ | 0.540 | 0.398 | 0.093 | 0 | -1.073 | -0.532 | -0.255 | -0.714 |
|  | MZQ | 1.160 | 1.005 | 0.130 | 15 | 1.454 | 0.848 | 1.863 | 2.345 |
|  | AAS | 0.923 | 0.717 | 0.114 | 4 | 0.487 | 0.192 | 0.955 | 0.102 |
|  | MIN self | 0.777 | 0.777 | 0.099 | 8 | -0.107 | 0.330 | 0.072 | 0.917 |
|  | NAR self | 0.926 | 0.792 | 0.093 | 3 | 0.501 | 0.364 | -0.251 | -0.102 |
|  | nSCH | 0.623 | 0.404 | 0.085 | 0 | -0.735 | -0.518 | -0.720 | -0.714 |
|  | dSCH | 0.609 | 0.609 | 0.079 | 0 | -0.790 | -0.052 | -1.020 | -0.714 |
|  | STAI-T | 1.086 | 1.086 | 0.108 | 5 | 1.153 | 1.034 | 0.600 | 0.306 |
|  | AAQ-2 | 0.960 | 0.960 | 0.103 | 0 | 0.638 | 0.746 | 0.325 | -0.714 |
|  | MSPSS | 0.428 | -0.428 | 0.070 | 0 | -1.527 | -2.413 | -1.569 | -0.714 |
| **mNTP** | AQ | 0.998 | 0.830 | 0.127 | 3 | 0.218 | 0.325 | 0.096 | -0.262 |
|  | MZQ | 1.145 | 1.145 | 0.153 | 6 | 1.005 | 0.996 | 1.678 | 0.862 |
|  | AAS | 1.138 | 0.642 | 0.139 | 7 | 0.966 | -0.074 | 0.810 | 1.237 |
|  | MIN self | 0.826 | 0.740 | 0.119 | 2 | -0.704 | 0.134 | -0.400 | -0.637 |
|  | NAR self | 1.005 | 1.005 | 0.129 | 7 | 0.254 | 0.698 | 0.206 | 1.237 |
|  | nSCH | 0.969 | 0.403 | 0.128 | 4 | 0.059 | -0.583 | 0.183 | 0.112 |
|  | dSCH | 0.833 | 0.833 | 0.111 | 1 | -0.669 | 0.332 | -0.856 | -1.012 |
|  | STAI-T | 1.124 | 0.821 | 0.137 | 6 | 0.893 | 0.306 | 0.695 | 0.862 |
|  | AAQ-2 | 1.004 | 0.886 | 0.118 | 1 | 0.246 | 0.445 | -0.445 | -1.012 |
|  | MSPSS | 0.535 | -0.535 | 0.093 | 0 | -2.267 | -2.579 | -1.967 | -1.386 |
|  |  |  |  |  |  |  |  |  |  |
| **Group** | **Node** | **Strength** | **Expected influence** | **Closeness** | **Betweenness** | **Strength**  **z-score** | **Expected influence**  **z-score** | **Closeness**  **z-score** | **Betweenness**  **z-score** |
| **SCH** | AQ | 0.857 | 0.857 | 0.132 | 4 | -0.301 | 0.536 | -0.500 | 0.621 |
|  | MZQ | 0.905 | 0.905 | 0.145 | 3 | 0.057 | 0.624 | 1.026 | -0.069 |
|  | AAS | 1.000 | 0.438 | 0.139 | 4 | 0.766 | -0.232 | 0.364 | 0.621 |
|  | MIN self | 0.866 | 0.824 | 0.125 | 1 | -0.232 | 0.477 | -1.253 | -1.449 |
|  | NAR self | 1.063 | 0.701 | 0.141 | 3 | 1.233 | 0.251 | 0.549 | -0.069 |
|  | nSCH | 0.678 | -0.020 | 0.123 | 2 | -1.632 | -1.072 | -1.458 | -0.759 |
|  | dSCH | 0.813 | 0.813 | 0.130 | 2 | -0.622 | 0.456 | -0.661 | -0.759 |
|  | STAI-T | 0.997 | 0.789 | 0.141 | 2 | 0.745 | 0.412 | 0.538 | -0.759 |
|  | AAQ-2 | 1.065 | 1.065 | 0.151 | 6 | 1.247 | 0.917 | 1.664 | 2.001 |
|  | MSPSS | 0.728 | -0.728 | 0.134 | 4 | -1.259 | -2.369 | -0.269 | 0.621 |

###

### Figure S8. Node centrality metrics (strength, expected influence, closeness, and betweenness) across diagnostic groups
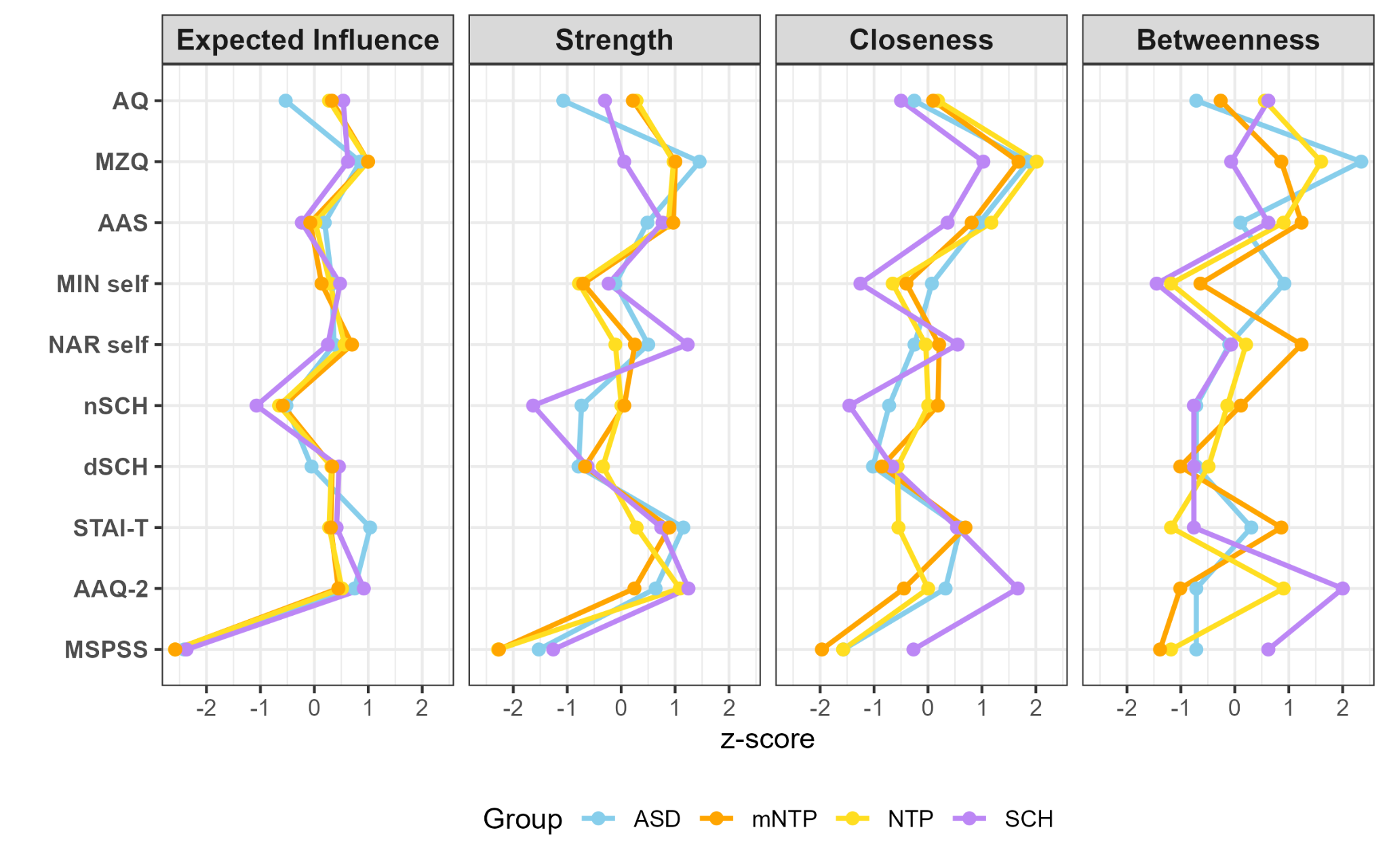


*Note*. ASD = autism spectrum disorder, mNTP = matched neurotypicals, NTP = non-matched neurotypicals, SCH = schizophrenia group, AQ = Autism Spectrum Quotient, dSCH = disorganized schizotypy, nSCH = negative schizotypy, STAI-T = trait anxiety, AAQ-2 = psychological inflexibility, AAS = insecure attachment, MZQ = mentalization, MIN self = minimal self, NAR self = narrative self.

## Stability

### Figure S9. Case dropping bootstrap in matched NTP group


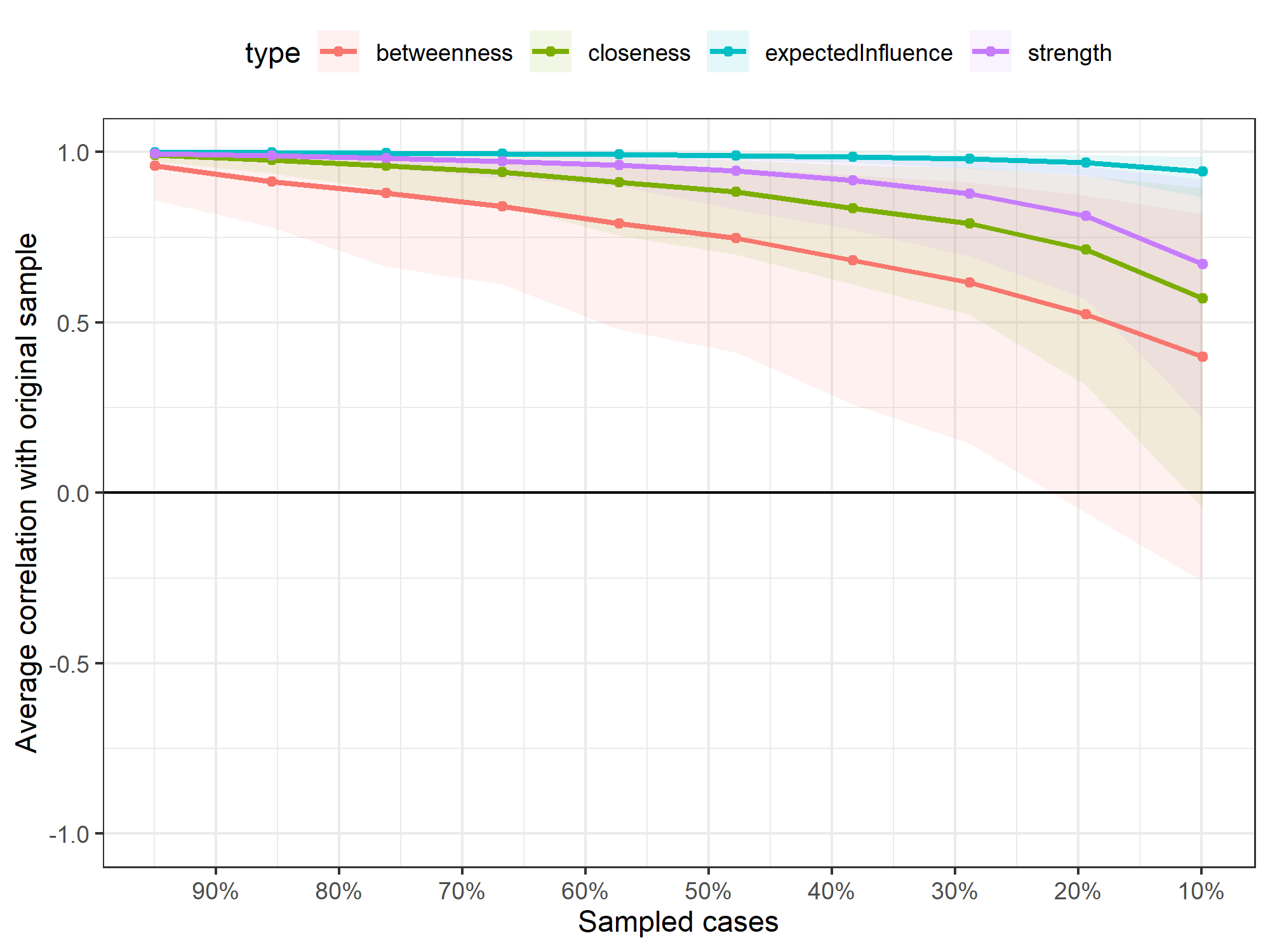


*Note*. Estimation of stability of node centralities with case-dropping bootstrap. Lines show the means and areas show the range between the 2.5th and the 97.5th quantiles

### Figure S10. Case dropping bootstrap in ASD group


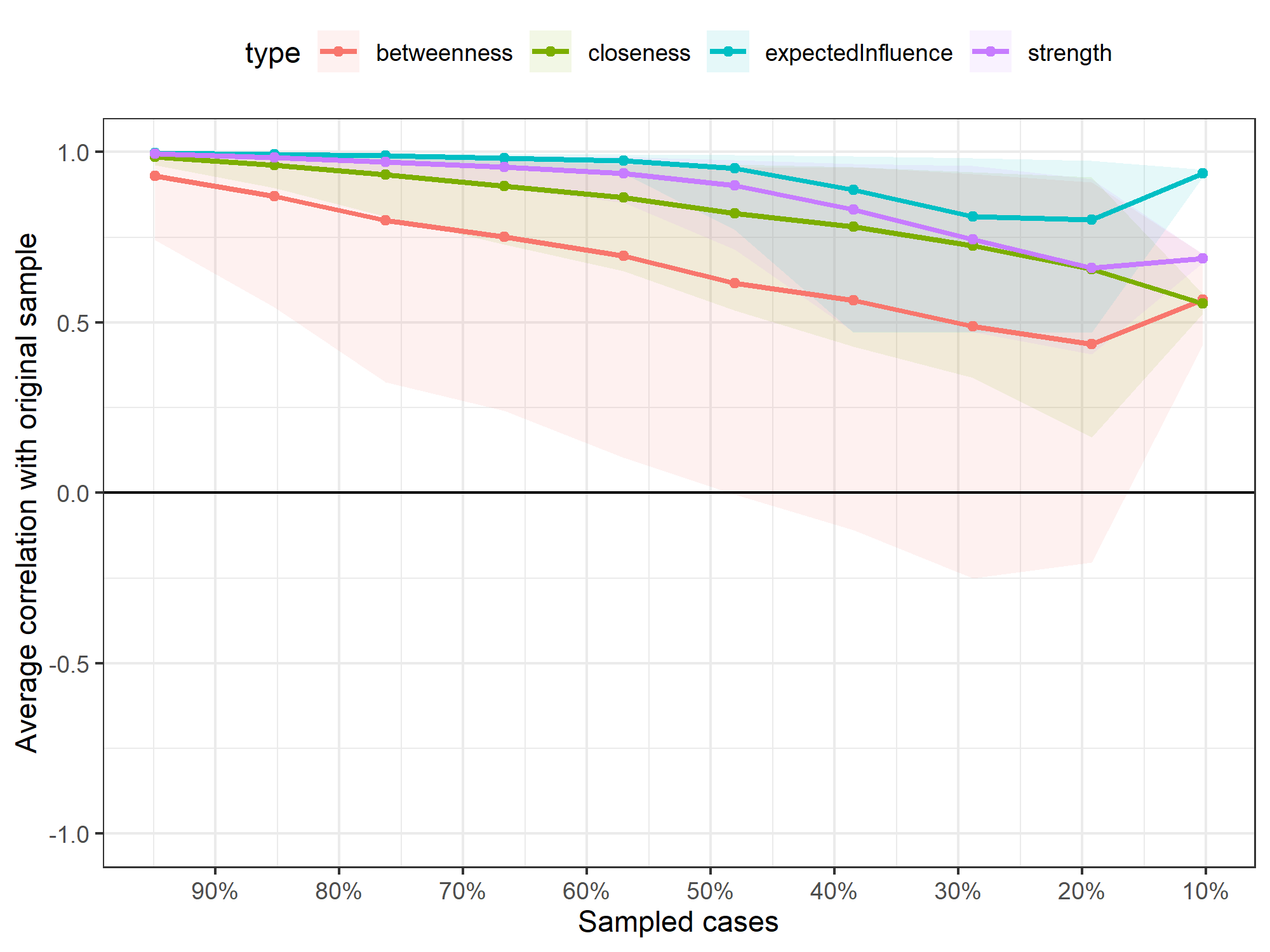


*Note*. Estimation of stability of node centralities with case-dropping bootstrap. Lines show the means and areas show the range between the 2.5th and the 97.5th quantiles

### Figure S11. Case dropping bootstrap in SCH group


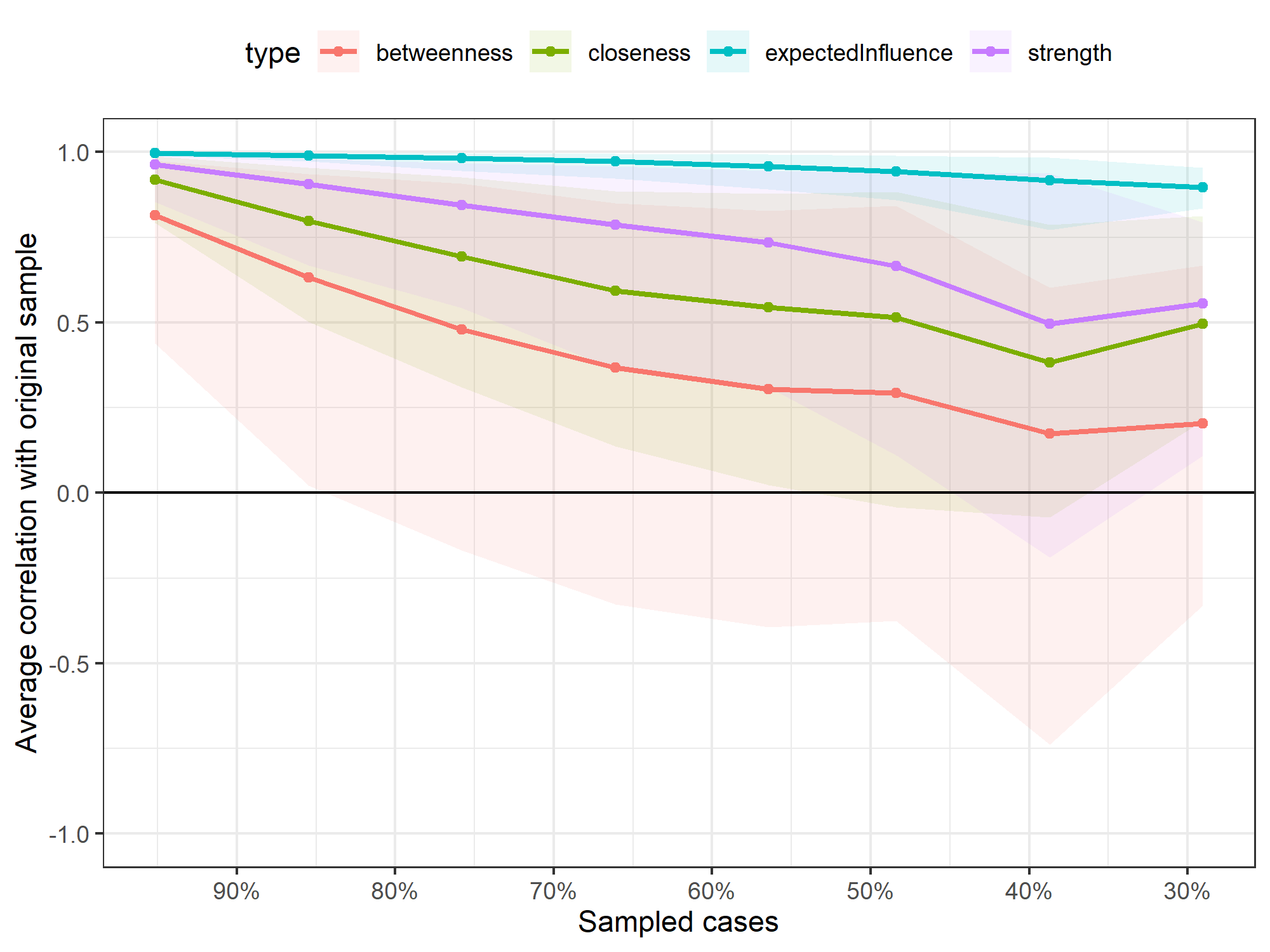


*Note*. Estimation of stability of node centralities with case-dropping bootstrap. Lines show the means and areas show the range between the 2.5th and the 97.5th quantiles

### Figure S12. Bootstrapped confidence intervals of all edges and stability test for edge-weight differences in mNTP group

###
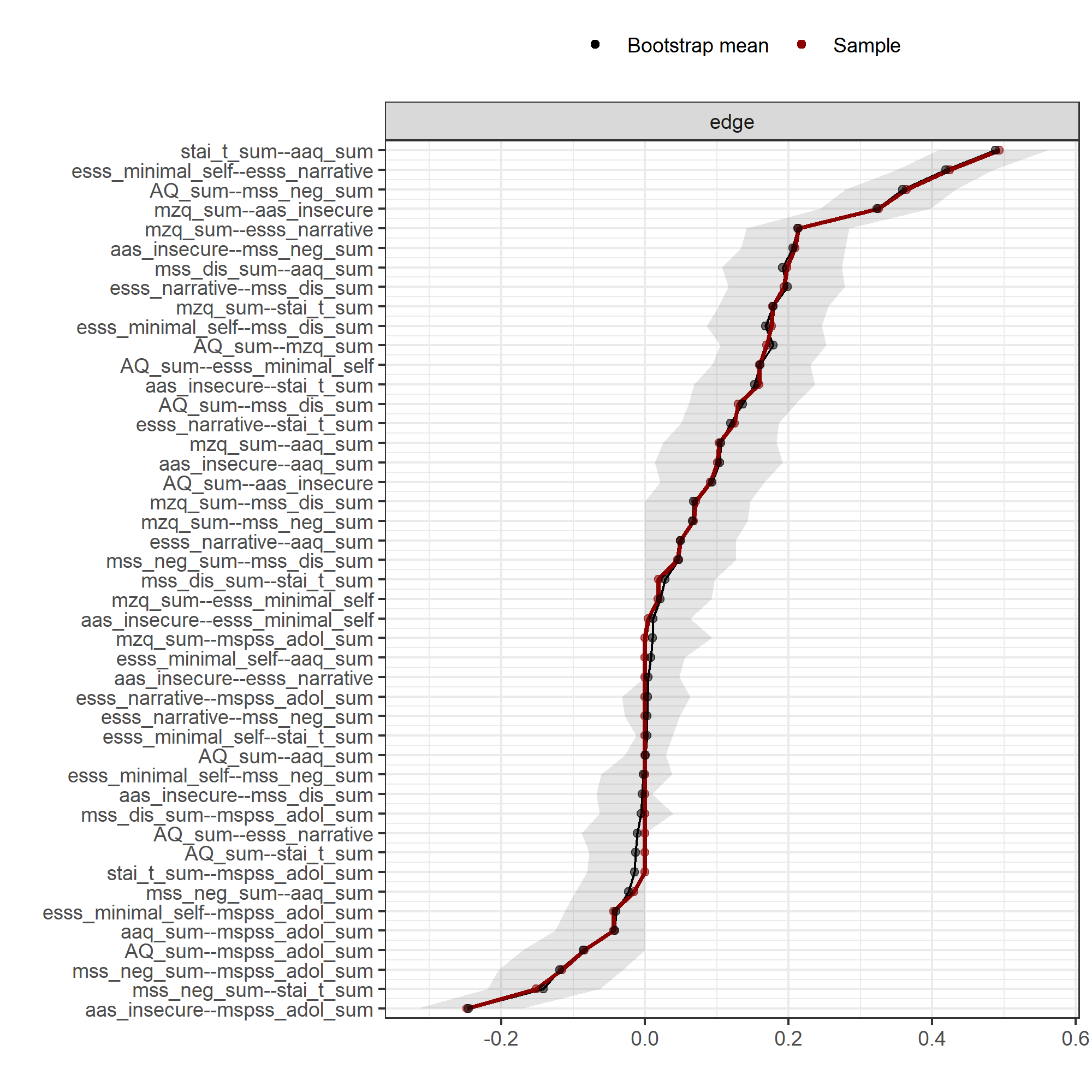

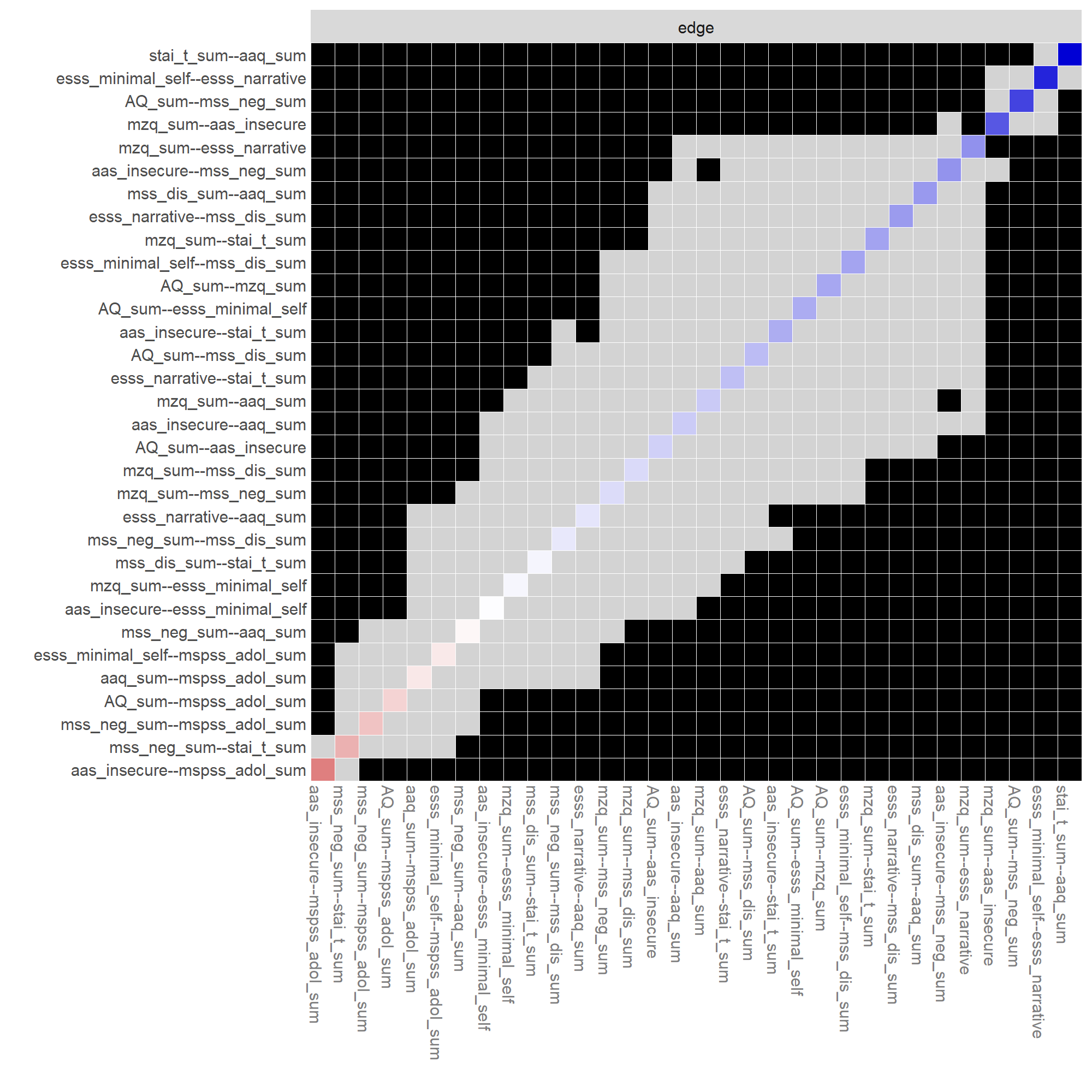


*Note*. Top: Bootstrapped confidence intervals (indicated by gray areas) and bootstrapped means for the estimated edge-weights. Bottom: Bootstrapped difference tests of edge weights in the mNTP network (α = 0.05). Significant differences are indicated by black boxes. Colors on the diagonal show edge weights.

### Figure S13. Bootstrapped difference tests between node centralities in mNTP group

###
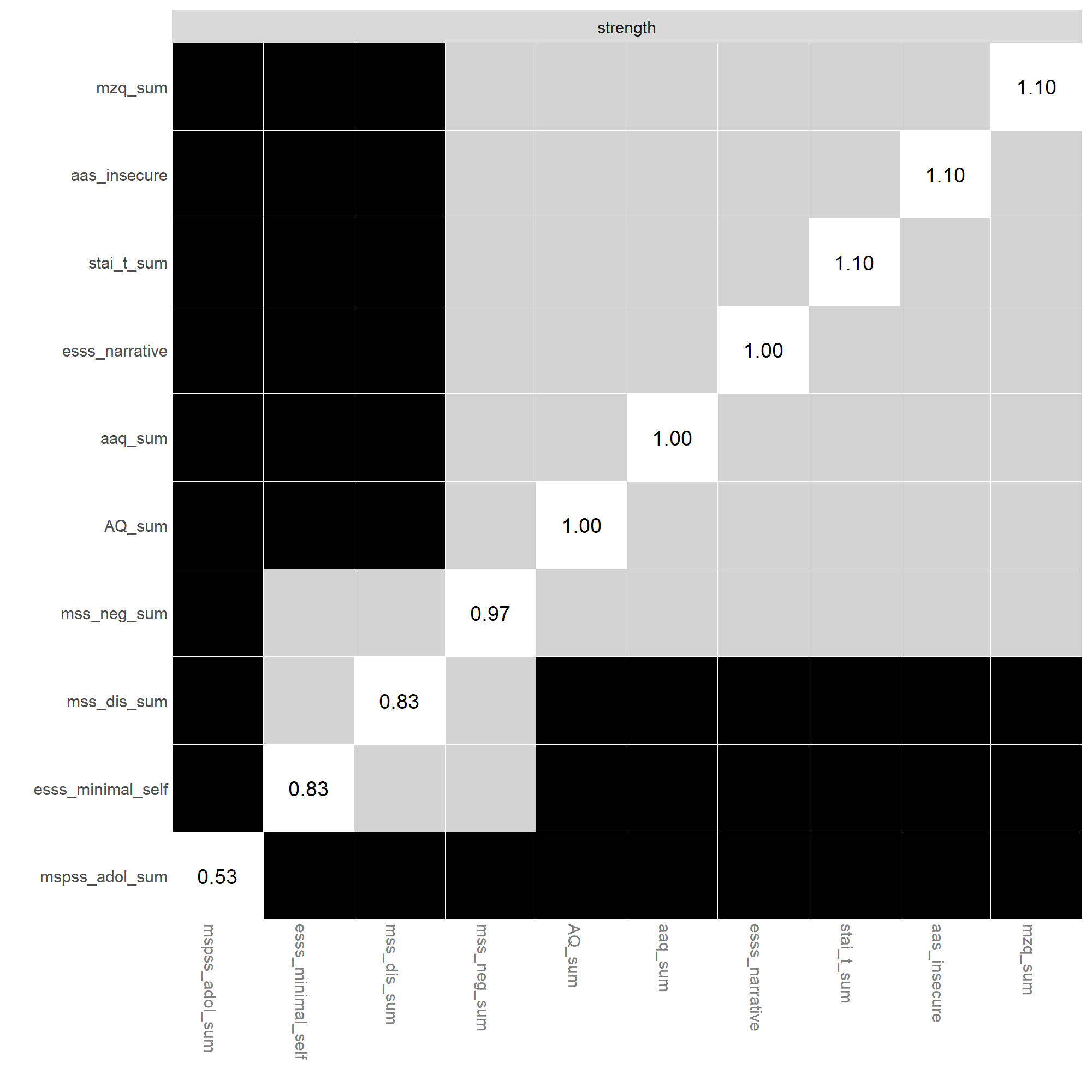

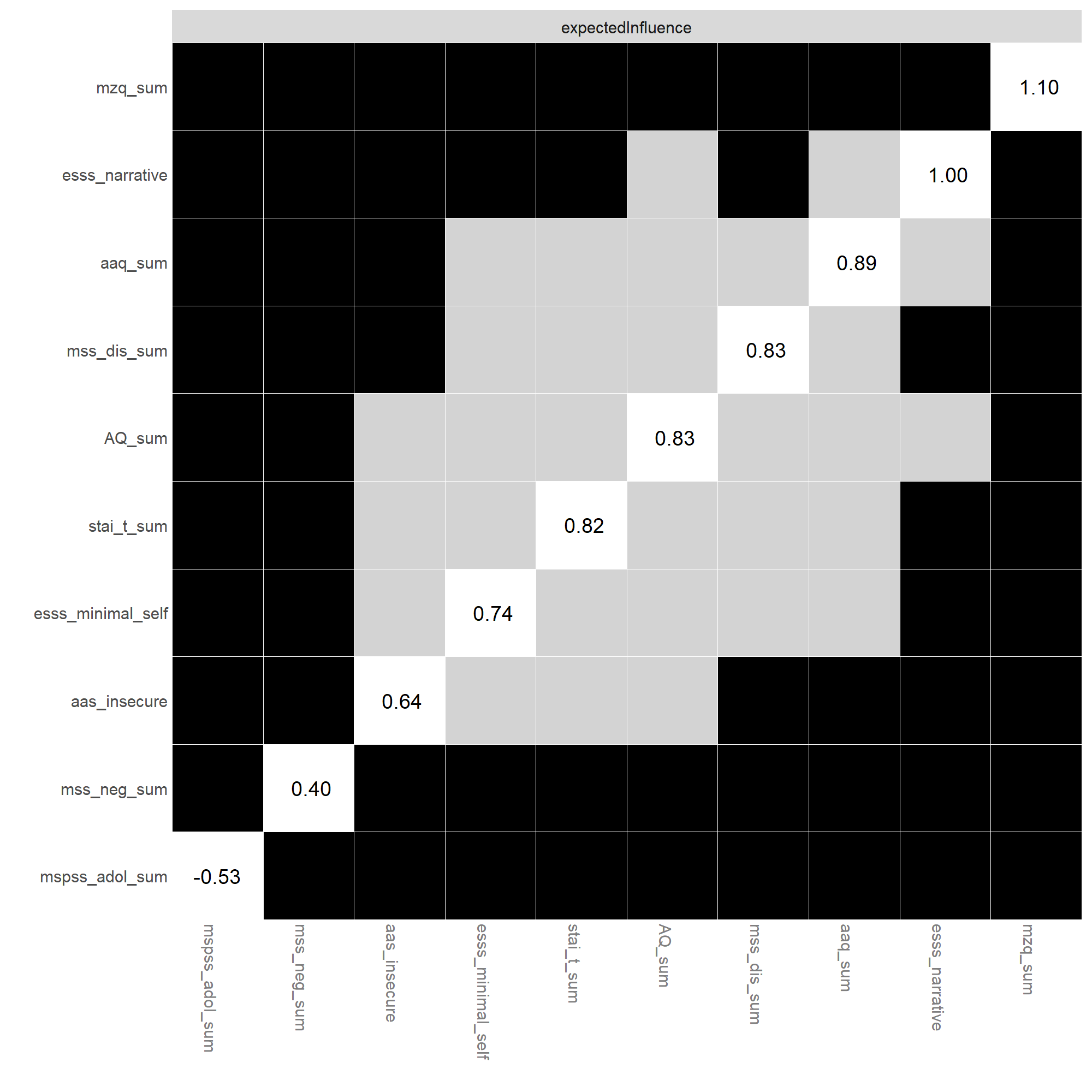

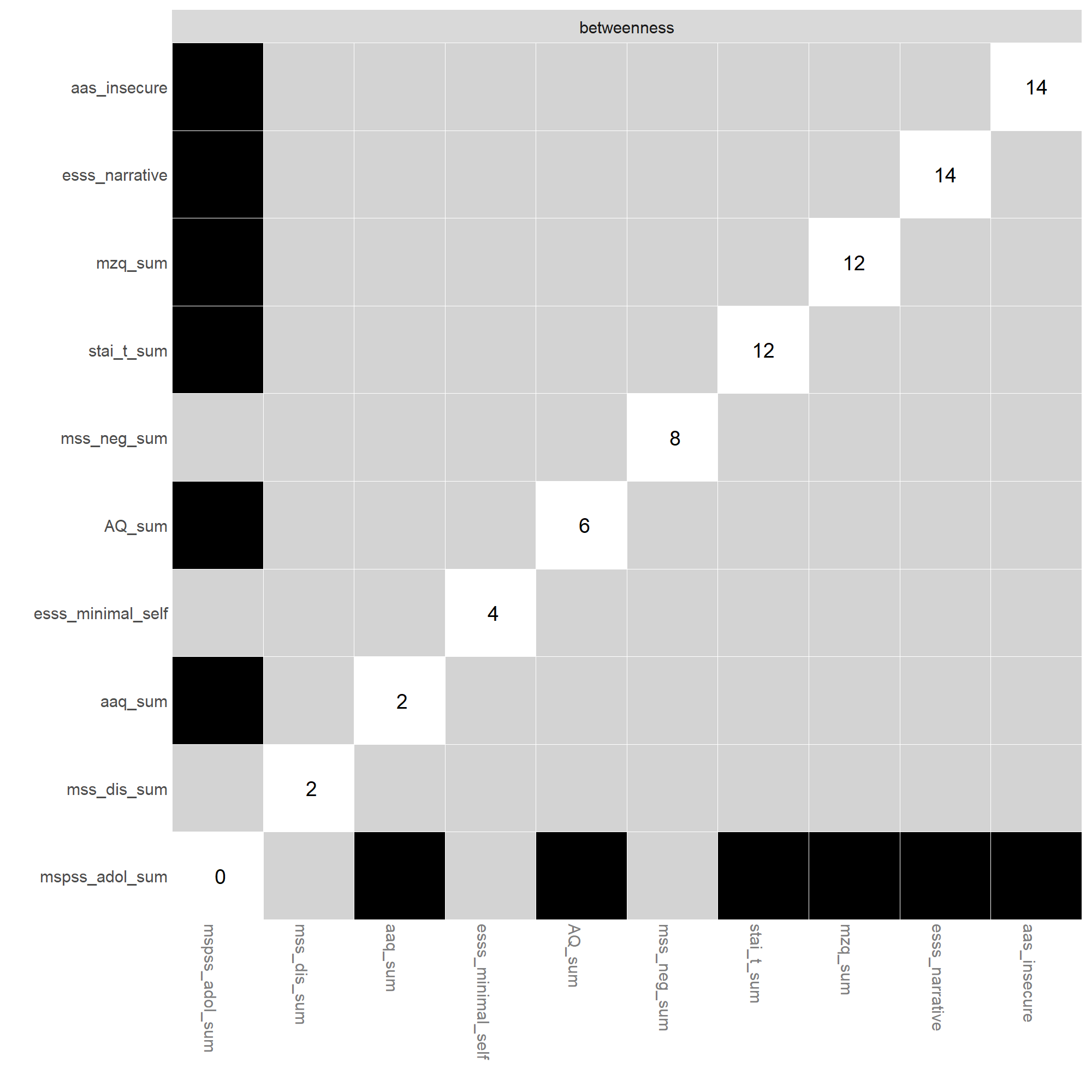

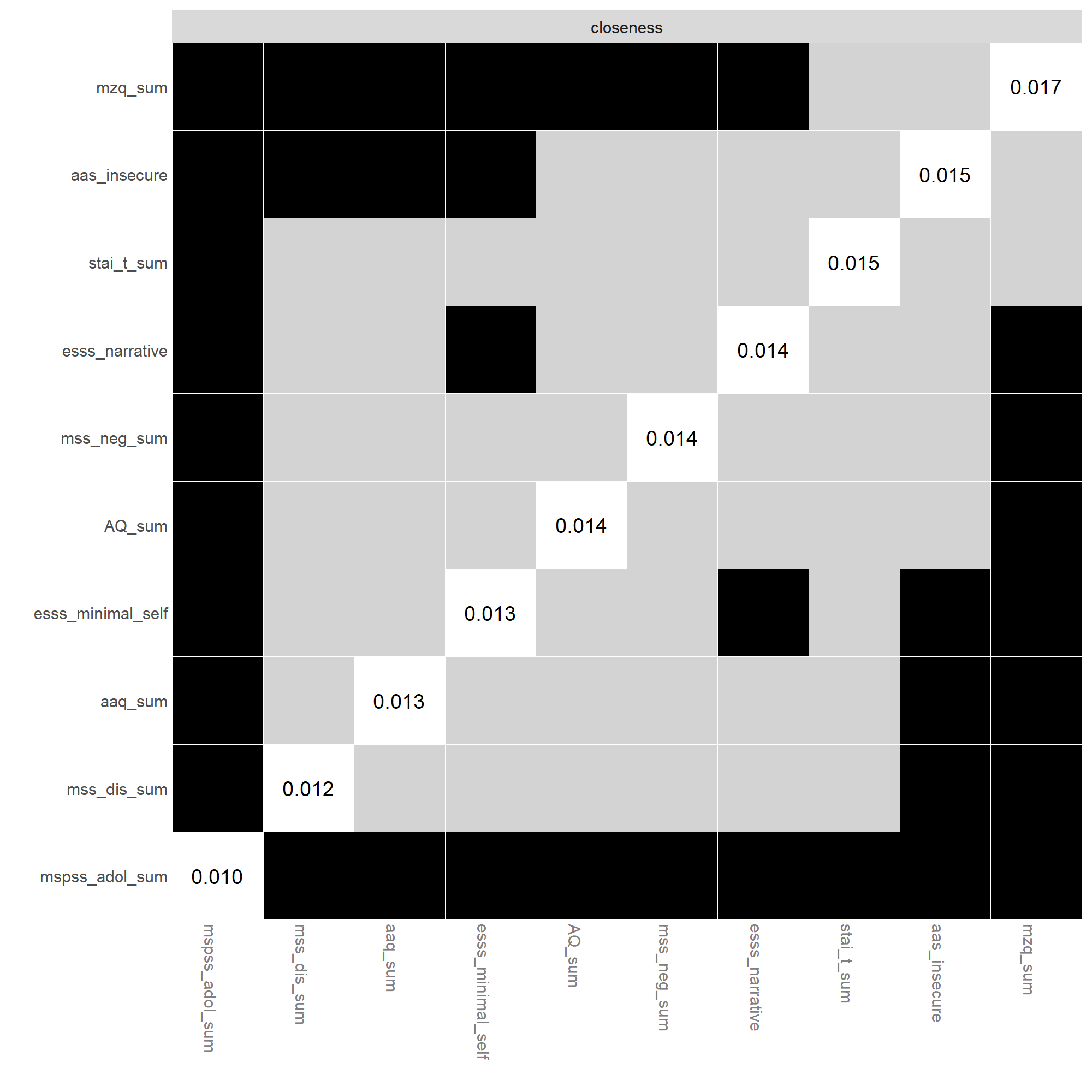


*Note*. Bootstrapped difference tests between node strength, expected influence, closeness and betweenness centralities in the ASD network. Significant differences (α = 0.05) are indicated by black boxes. Nodes are arranged in descending order according to centrality.

###

### Figure S14. Bootstrapped confidence intervals of all edges and stability test for edge-weight differences in the ASD group


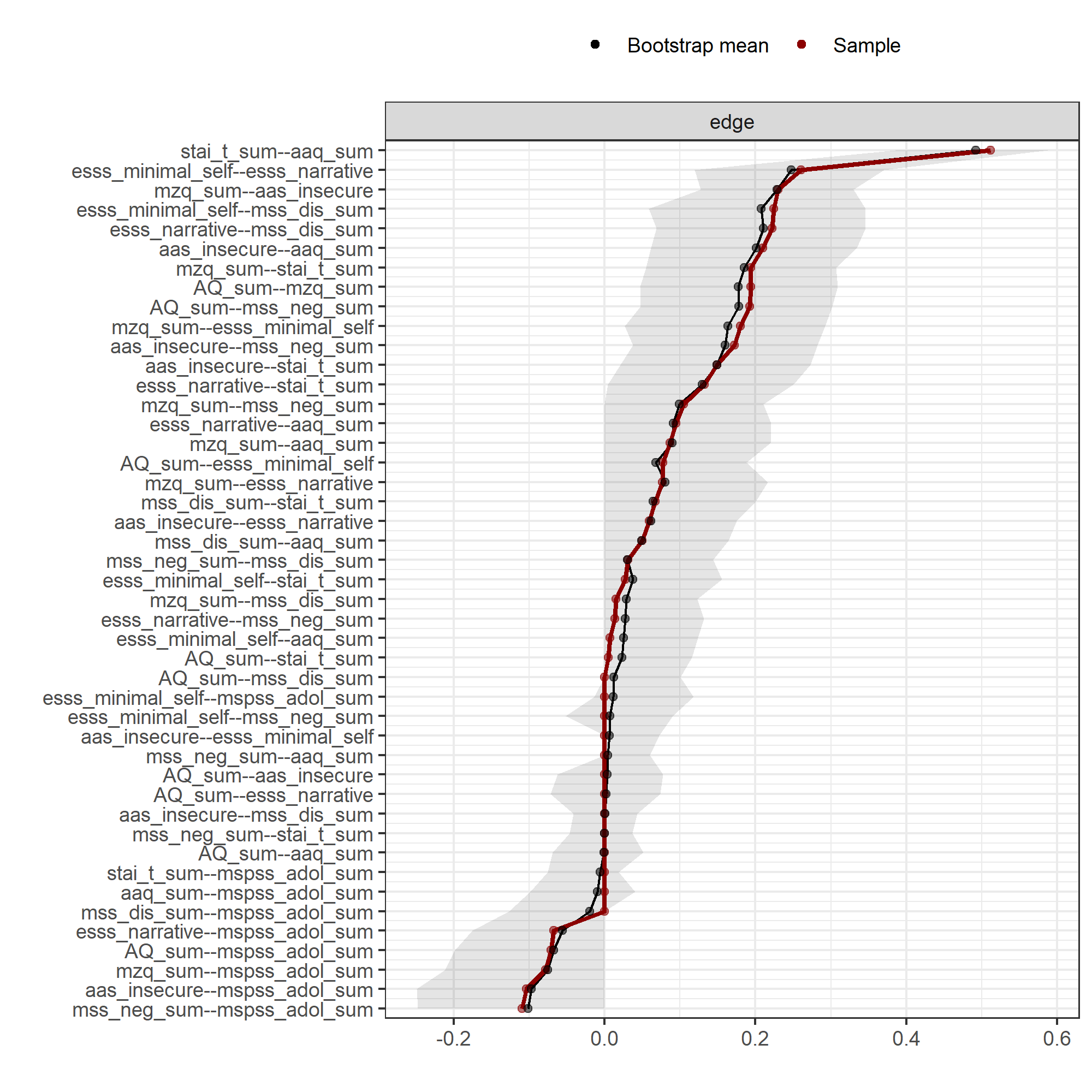

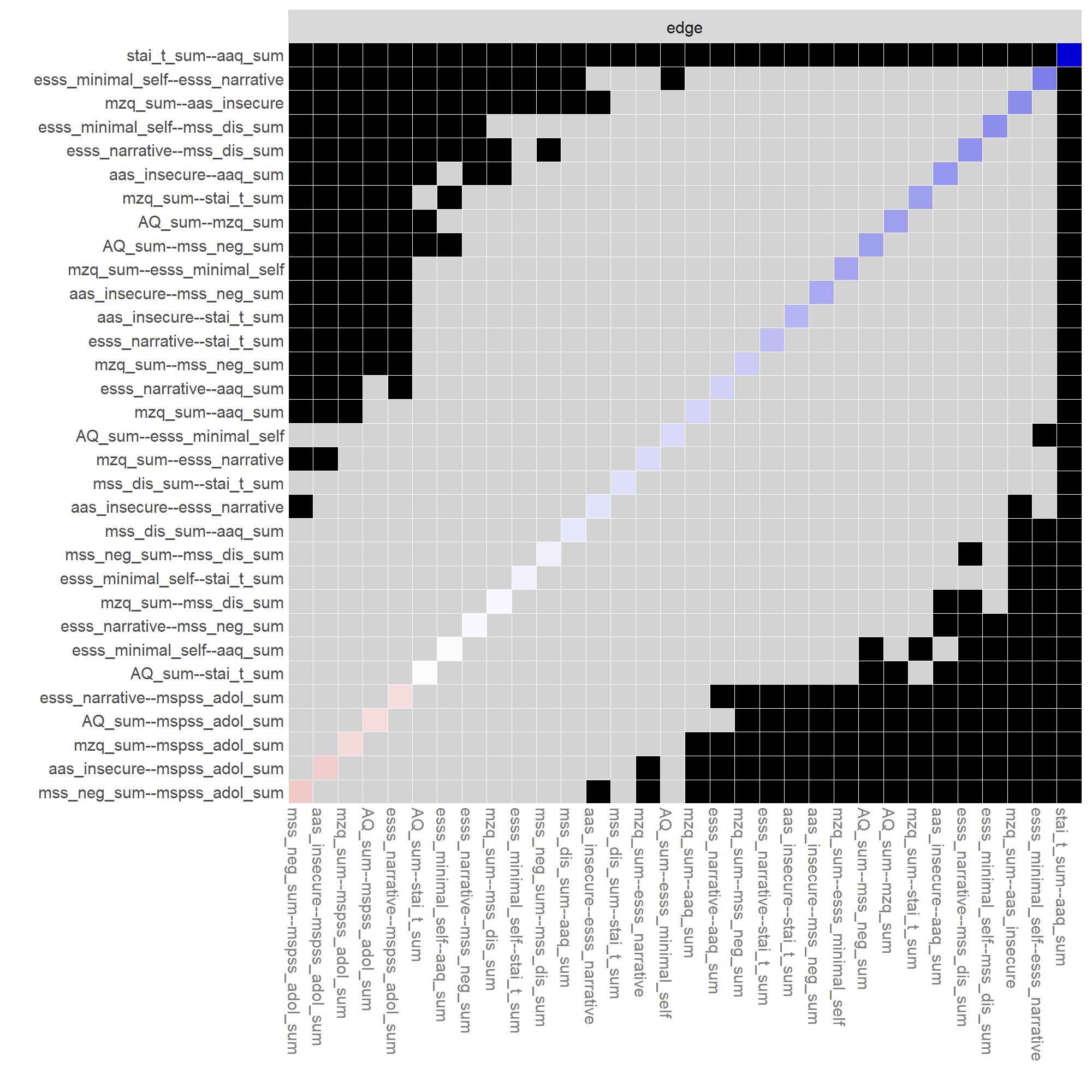


*Note*. Top: Bootstrapped confidence intervals (indicated by gray areas) and bootstrapped means for the estimated edge-weights. Bottom: Bootstrapped difference tests of edge weights in the ASD network (α = 0.05). Significant differences are indicated by black boxes. Colors on the diagonal show edge weights.

### Figure S15. Bootstrapped difference tests between node centralities in the ASD group


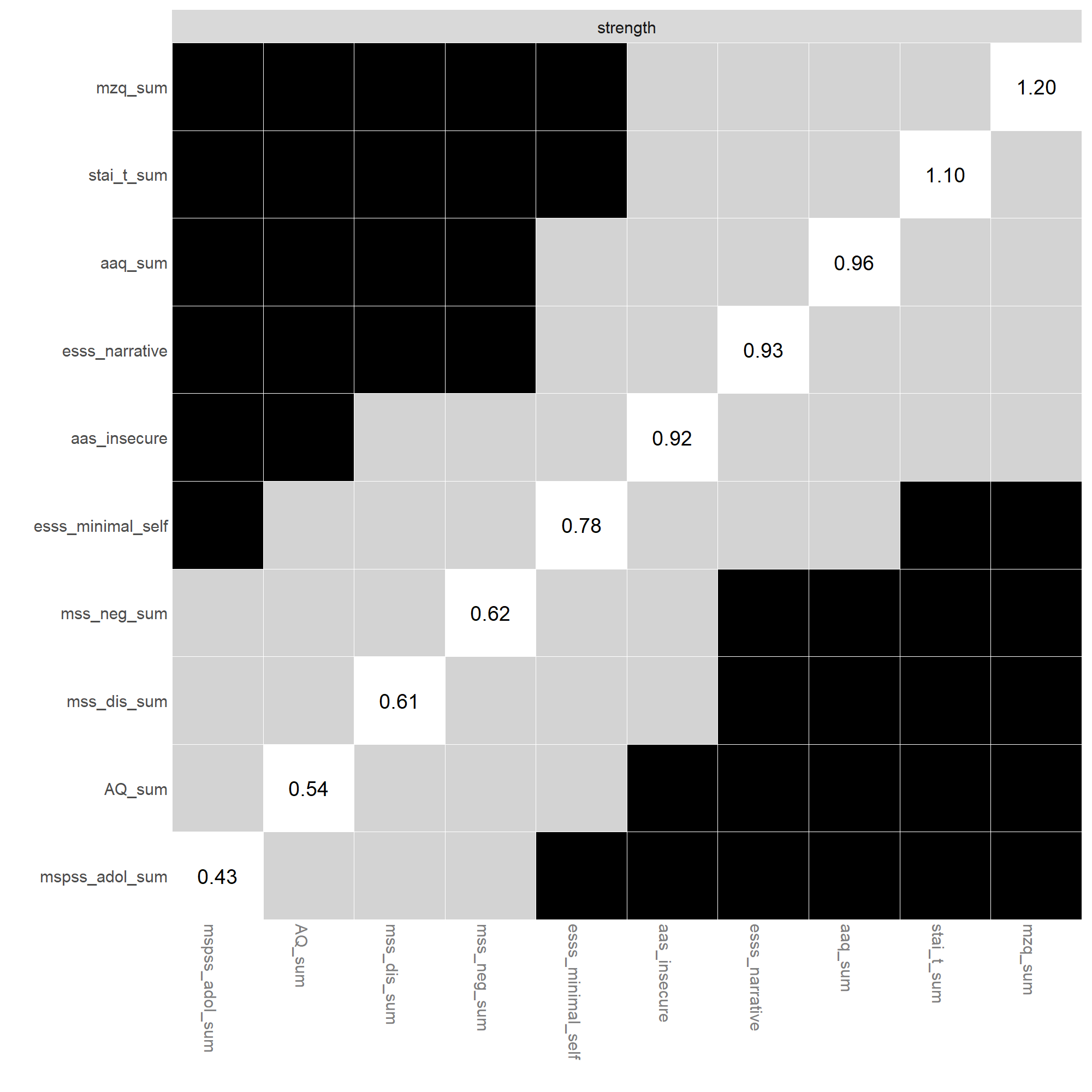

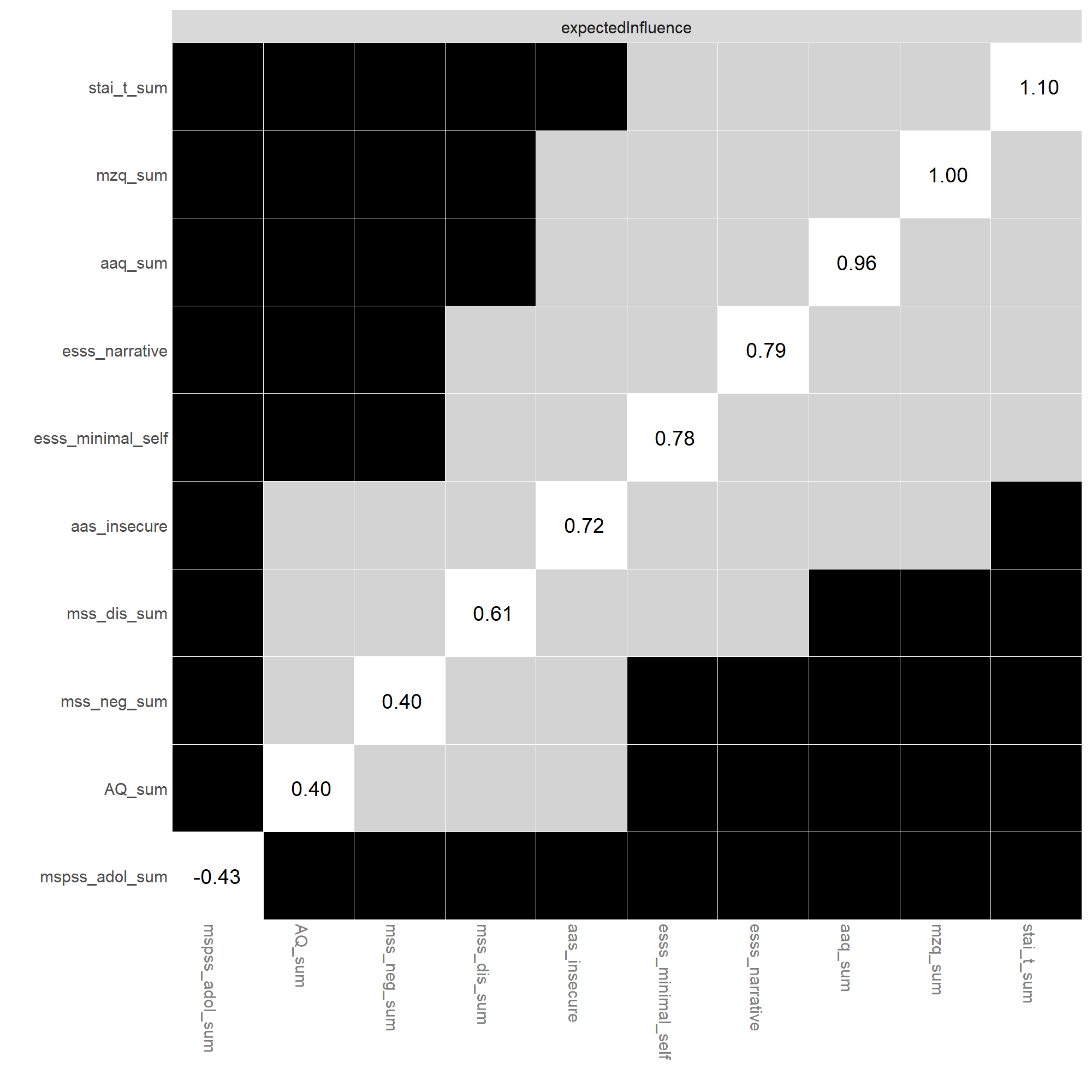


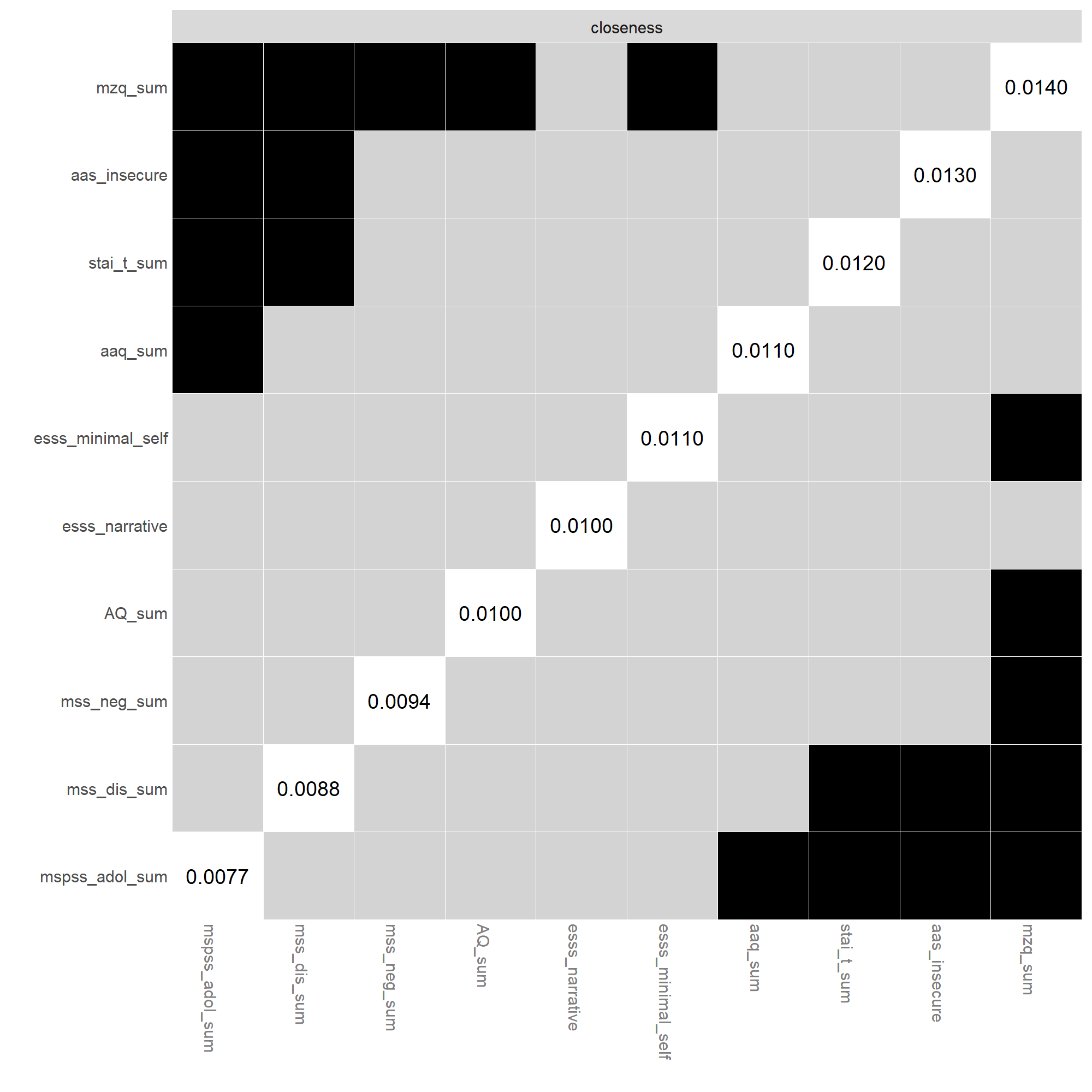

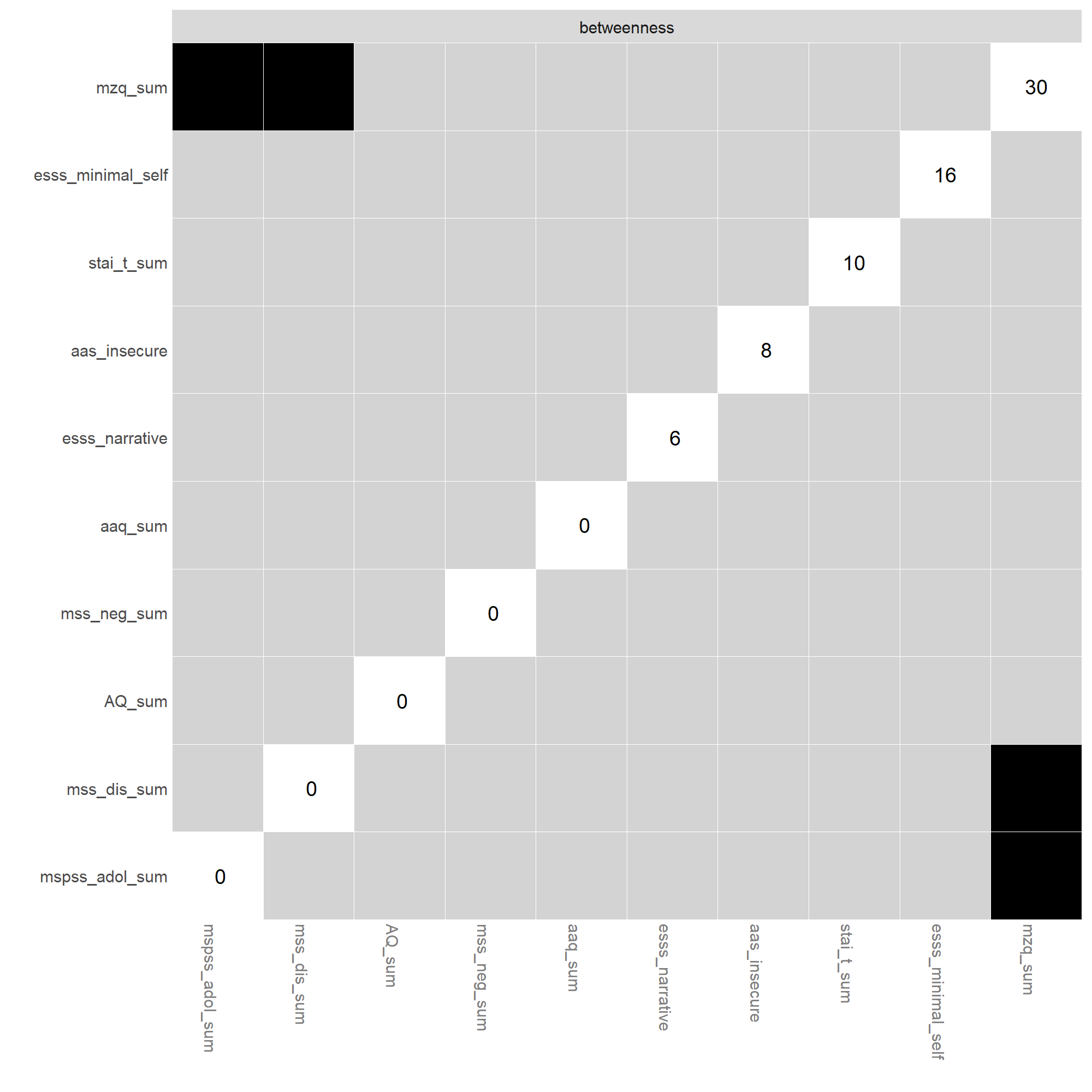


*Note*. Bootstrapped difference tests between node strength, expected influence, closeness and betweenness centralities in the ASD network. Significant differences (α = 0.05) are indicated by black boxes. Nodes are arranged in descending order according to centrality.

### Figure S16. Bootstrapped confidence intervals of all edges and stability test for edge-weight differences in SCH group


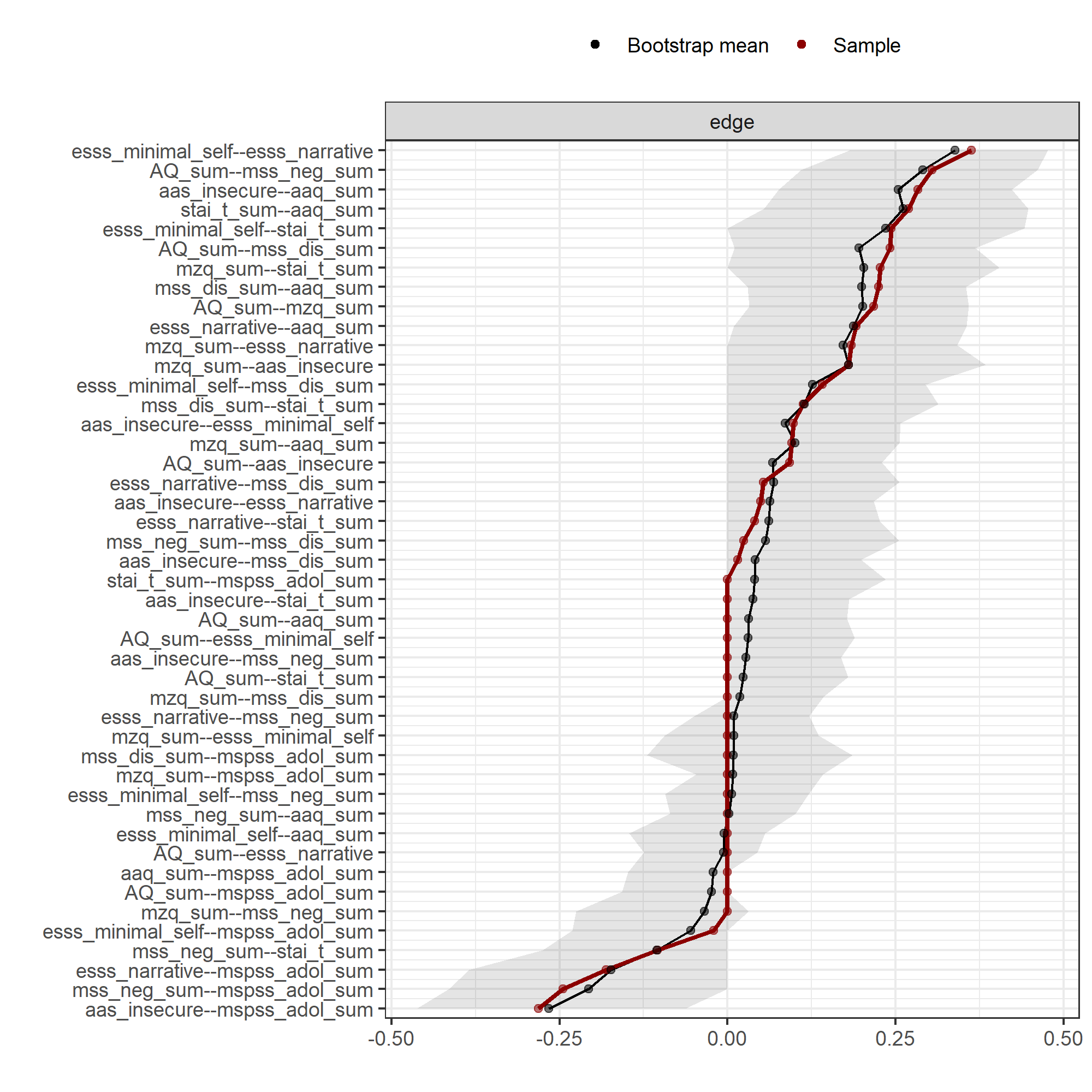

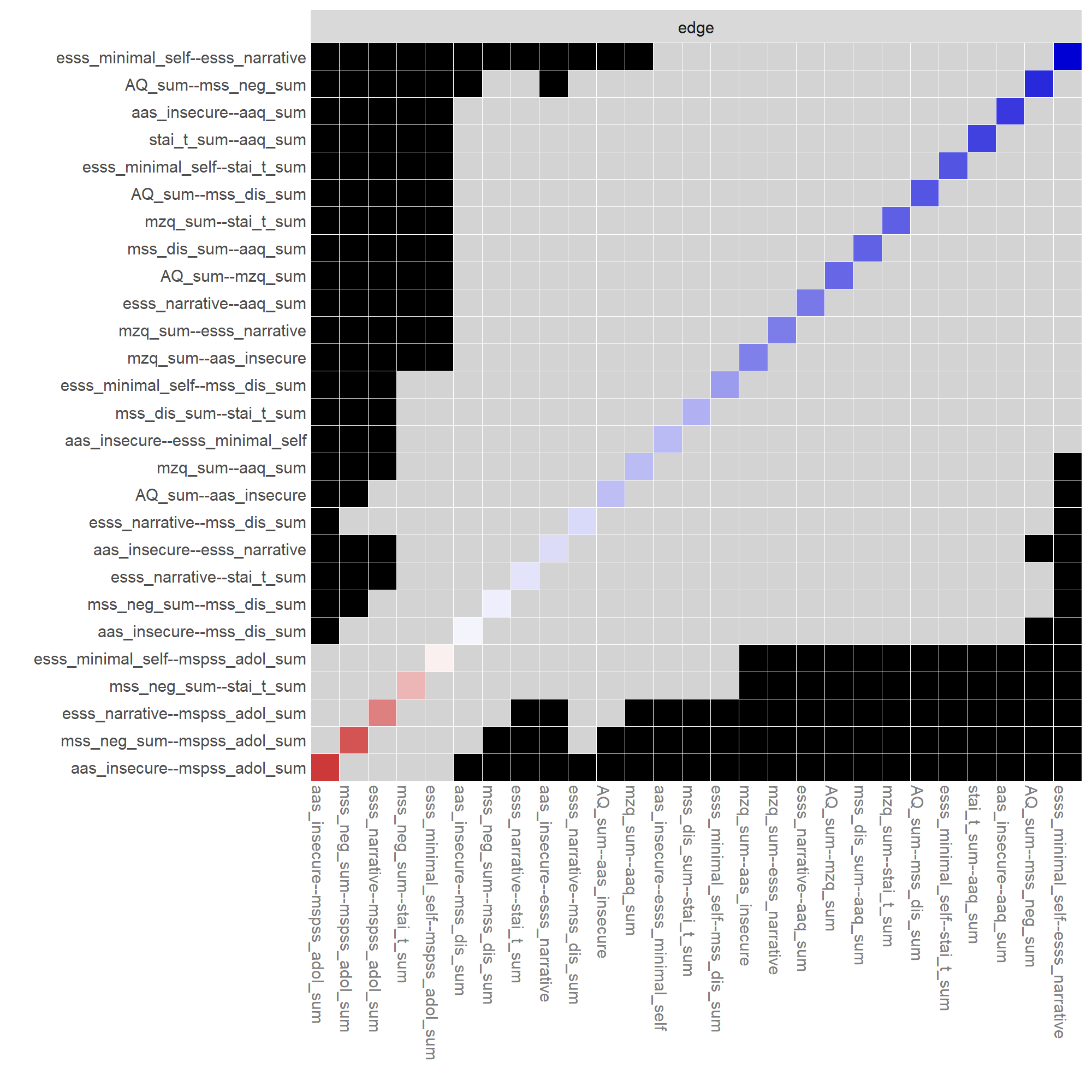


*Note*. Top: Bootstrapped confidence intervals (indicated by gray areas) and bootstrapped means for the estimated edge-weights. Bottom: Bootstrapped difference tests of edge weights in the SCH network (α = 0.05). Significant differences are indicated by black boxes. Colors on the diagonal show edge weights.

### Figure S17. Bootstrapped difference tests between node centralities in the SCH group


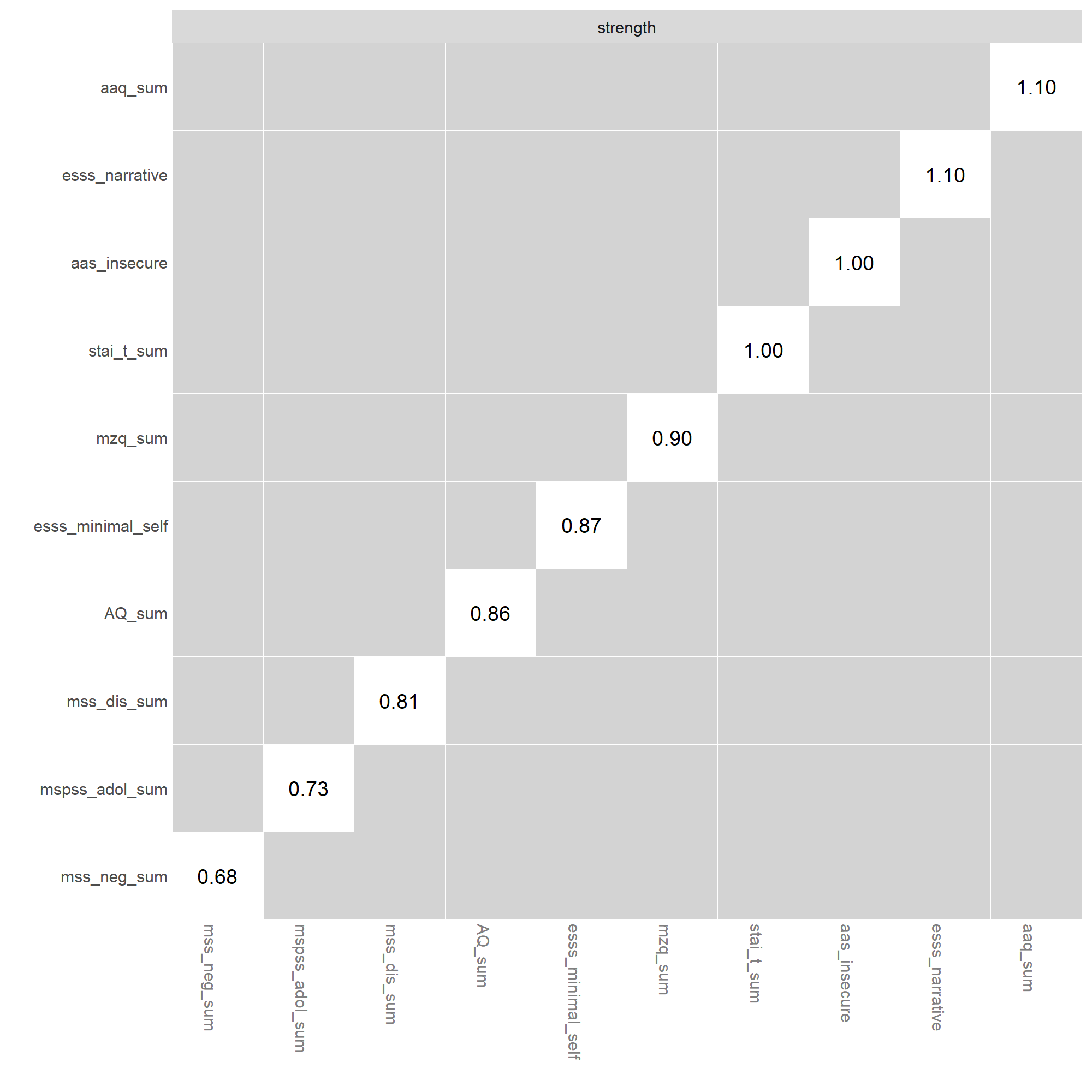

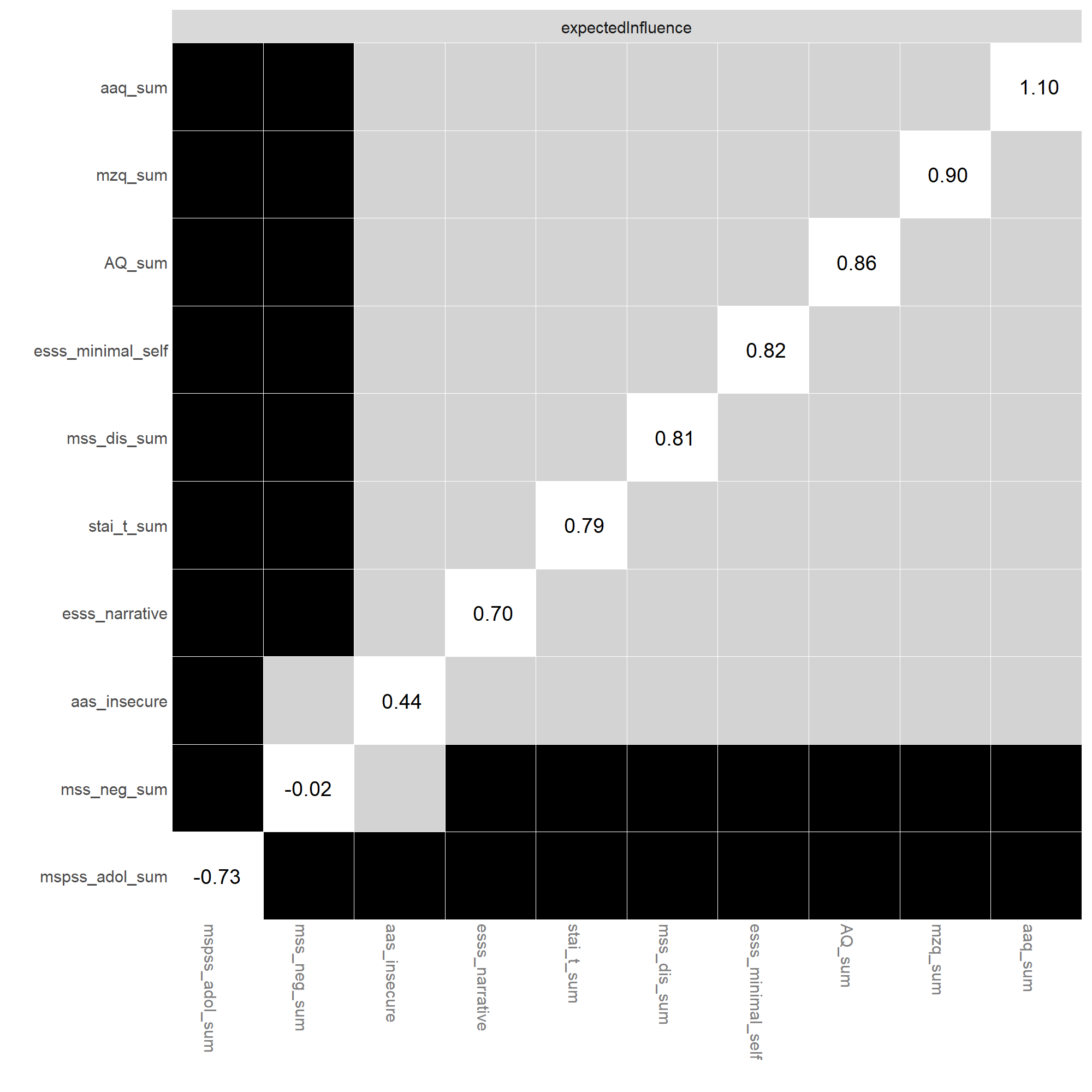


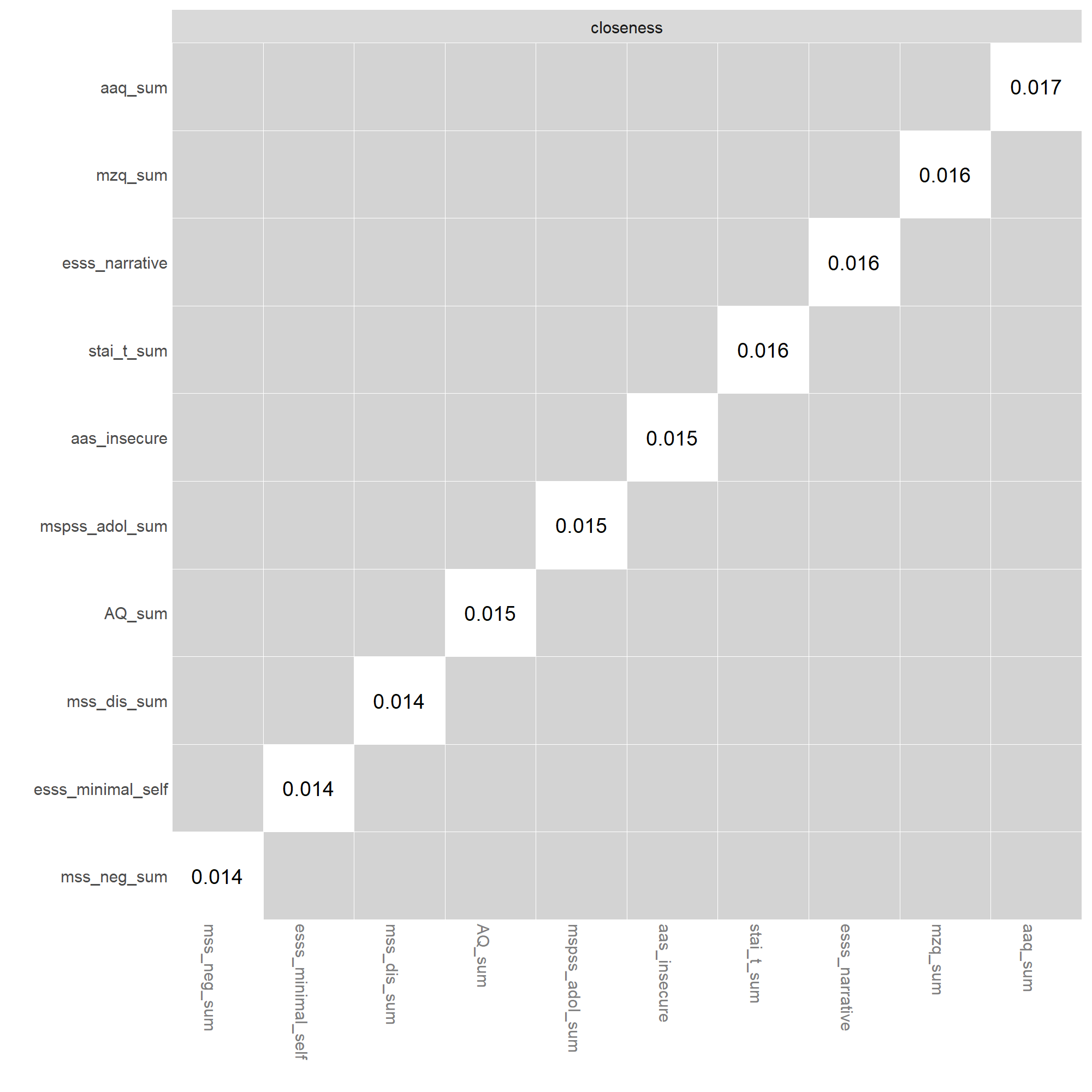

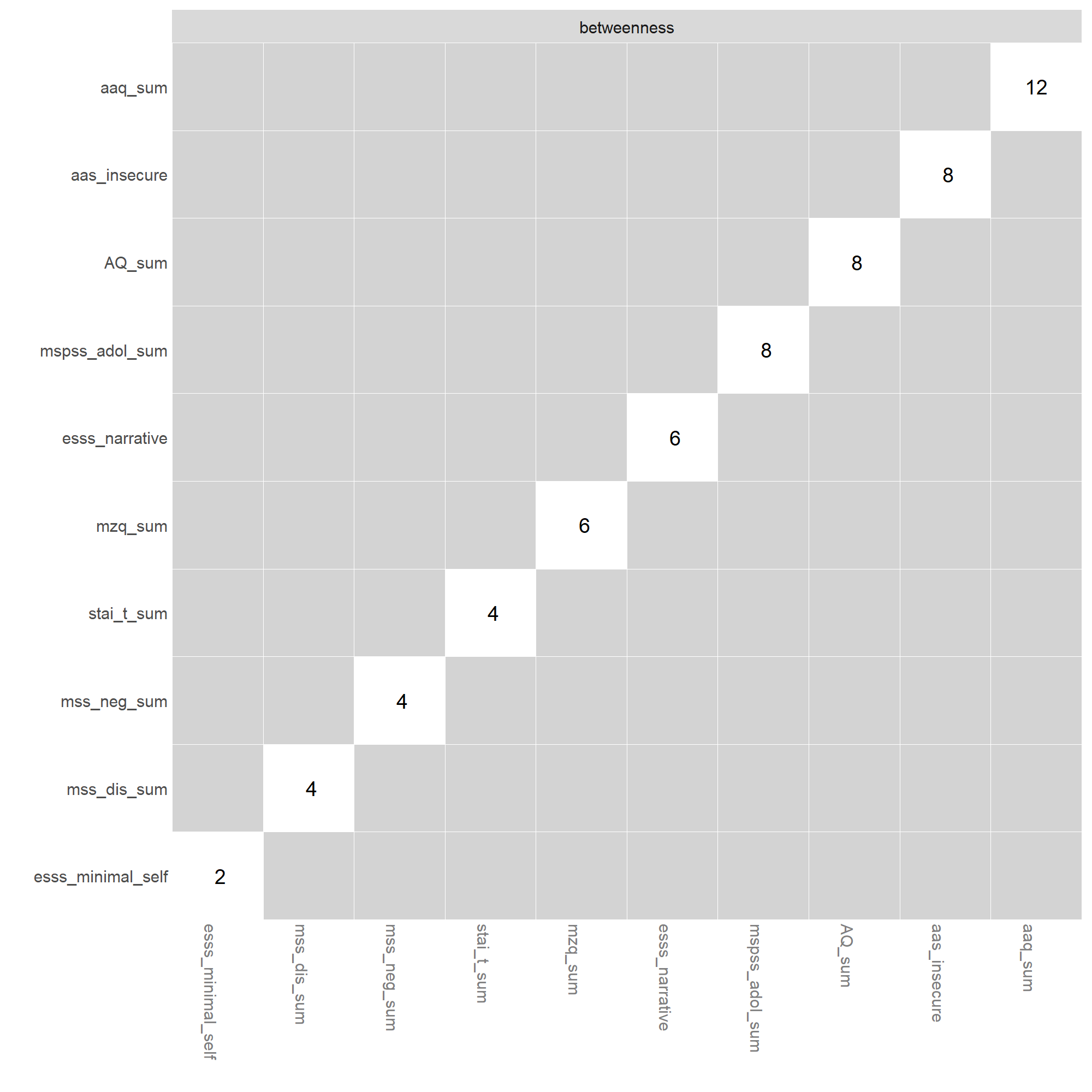


*Note*. Bootstrapped difference tests between node strength, expected influence, closeness and betweenness centralities in the SCH network. Significant differences (α = 0.05) are indicated by black boxes. Nodes are arranged in descending order according to centrality.

### Table S8. Correlation Stability (CS) coefficients

| **Centrality metric** | **ASD** | **mNTP** | **SCH** |
| --- | --- | --- | --- |
| Betweenness | 0.051 | 0.238 | 0 |
| Closeness | 0.333 | 0.522 | 0.048 |
| Expected influence | 0.519 | 0.901 | 0.145 |
| Strength | 0.519 | 0.711 | 0.145 |

*Note*. ASD = autism spectrum disorder, mNTP = matched neurotypicals, SCH = schizophrenia spectrum

#

# Network comparison

### Table S9. Node-level network invariance

|  | **Strength** | | **Expected influence** | | Closeness | |
| --- | --- | --- | --- | --- | --- | --- |
| **Node** | **difference** | **p-value** | **difference** | **p-value** | difference | p-value |
| AQ_sum | -0.459 | **0.027** | -0.431 | **0.047** | -0.004 | 0.089 |
| mzq_sum | 0.015 | 0.923 | -0.141 | 0.400 | -0.003 | 0.281 |
| aas_insecure | -0.215 | 0.185 | 0.074 | 0.725 | -0.003 | 0.158 |
| esss_minimal_self | -0.049 | 0.725 | 0.037 | 0.798 | -0.002 | 0.220 |
| esss_narrative | -0.079 | 0.579 | -0.213 | 0.158 | -0.004 | 0.089 |
| mss_neg_sum | -0.346 | 0.174 | 0.001 | 0.994 | -0.005 | 0.047 |
| mss_dis_sum | -0.224 | *0.096* | -0.224 | 0.159 | -0.004 | 0.096 |
| stai_t_sum | -0.038 | 0.877 | 0.265 | *0.096* | -0.003 | 0.185 |
| aaq_sum | -0.044 | 0.768 | 0.074 | 0.666 | -0.002 | 0.407 |
| mspss_adol_sum | -0.107 | 0.407 | 0.107 | 0.407 | -0.003 | 0.129 |

*Note*. Node-level network invariance in ASD vs. mNTP group comparison

### Figure S18. The partial correlation network structures of the three groups


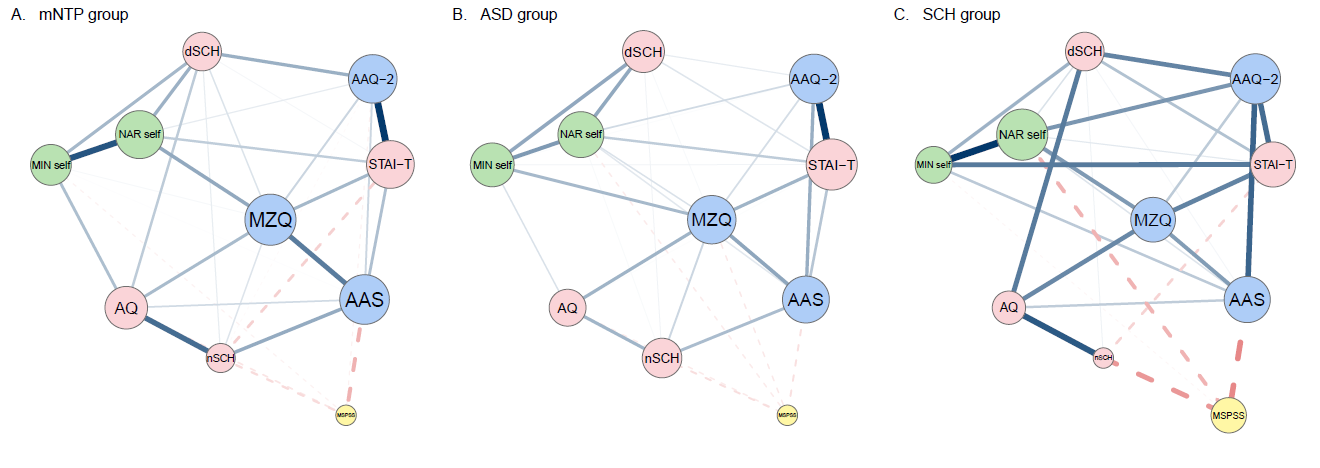


*Notes*. mNTP = matched neurotypicals, ASD = autism spectrum disorder, SCH = schizophrenia group. AQ = Autism Spectrum Quotient, dSCH = disorganized schizotypy, nSCH = negative schizotypy, STAI-T = trait anxiety, AAQ-II = psychological inflexibility, AAS = insecure attachment, MZQ = mentalization, MIN self = minimal self, NAR self = narrative self, MSPSS = perceived social support.

# Sensitivity analysis

### Methods S4.

We conducted a sensitivity analysis in which STAI-T scores were included in the matching procedure used to create the comparison NTP subsample. In the original analyses, the matched NTP group was constructed to be comparable to the ASD group only on demographic characteristics. In the sensitivity analysis, we added trait anxiety to this matching procedure and then repeated the same analytical steps as in the main analysis. This allowed us to evaluate whether the observed differences were attributable to underlying group differences in anxiety levels. Note, that this procedure resulted in removing one participant from the ASD group.

The descriptive pattern was also broadly similar after STAI-based matching, besides the matched neurotypical group shifting towards the ASD profile on several key variables (Figure S19). This suggests that matching on trait anxiety produced a control group that was not only more similar to the ASD group in anxiety, but also somewhat closer on several related self- and social-cognitive characteristics. The group-comparison results showed the same general pattern after STAI-based matching, with attenuated differences reflected in lower effect sizes (Table S10A).

###

### Figure S19. Questionnaire mean scores by group (ASD_STAI vs. mNTP_STAI)
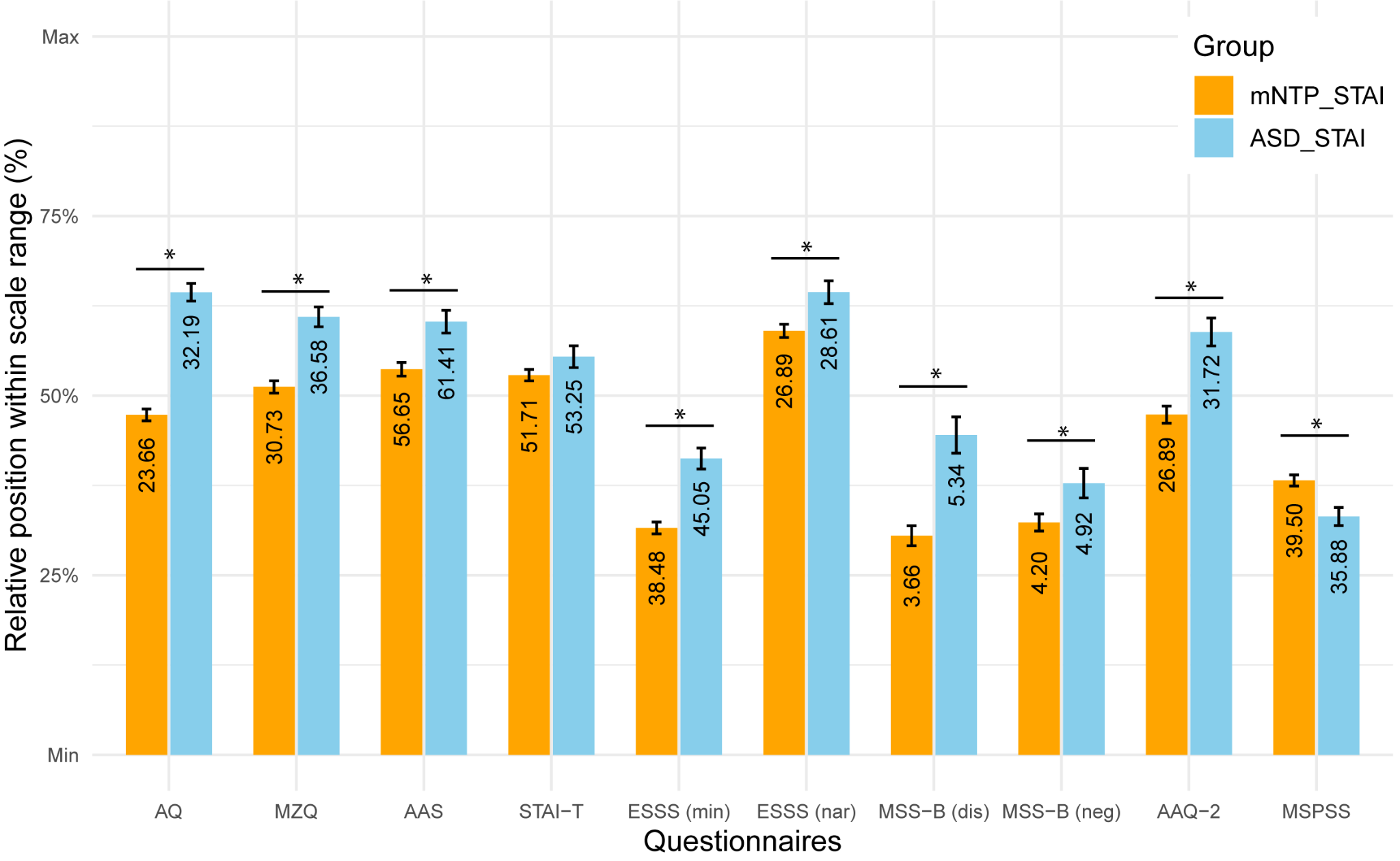


*Notes.* ASD_STAI = autism spectrum disorder in the STAI-T based matching, mNTP_STAI = matched neurotypical controls without psychiatric diagnosis matched based on STAI-T scores. AQ = Autism Spectrum Quotient, MZQ = Mentalization Questionnaire, AAS = Adult Attachment Scale, STAI-T = State-Trait Anxiety Inventory, trait anxiety, ESSS (min) and (nar) = Embodied Sense of Self Scale, MSS-B (dis) and (neg) = Multidimensional Schizotypy Scale - Brief disorganised and negative schizotypy subscales, AAQ-2 = Acceptance and Action Questionnaire-II), MSPSS = Multidimensional Scale of Perceived Social Support. error bars = standard error. Statistical significance for group differences is indicated by * (p < 0.05).

###

### Table S10. Descriptive statistics and group comparisons for psychological variables included in the analysis

| **Variable** | **Group** | ***Min - Max*** | ***Mean*** | ***SD*** | ***Mann-Whitney U*** | ***p-value*** | ***r (effect size)*** |
| --- | --- | --- | --- | --- | --- | --- | --- |
| Autistic traits (AQ-50) | mNTP_STAI | 4 – 43 | 23.66 | 8.72 | W = 53944 | < .001 | 0.40 |
|  | ASD_STAI | 12 – 49 | 32.19 | 7.63 |  |  |  |
| Mentalization (MZQ) | mNTP_STAI | 3 – 54 | 30.73 | 10.83 | W = 45342 | < .001 | 0.22 |
|  | ASD_STAI | 13 – 60 | 36.58 | 10.34 |  |  |  |
| Insecure attachment (AAS) | mNTP_STAI | 24 – 90 | 55.65 | 14.53 | W = 41492 | < .001 | 0.14 |
|  | ASD_STAI | 30 – 90 | 61.41 | 14.17 |  |  |  |
| Trait anxiety (STAI-T) | mNTP_STAI | 26 – 77 | 51.71 | 10.25 | W = 38270 | .103 | 0.07 |
|  | ASD_STAI | 25 – 77 | 53.25 | 11.31 |  |  |  |
| Minimal self (ESSS) | mNTP_STAI | 17 – 73 | 38.48 | 11.97 | W = 45492 | < .001 | 0.22 |
|  | ASD_STAI | 21 – 77 | 45.05 | 12.38 |  |  |  |
| Narrative self (ESSS) | mNTP_STAI | 10 – 40 | 26.89 | 6.27 | W = 40871 | .003 | 0.12 |
|  | ASD_STAI | 10 – 40 | 28.61 | 6.34 |  |  |  |
| Negative schizotypy (MSS-B) | mNTP_STAI | 0 – 13 | 4.20 | 3.30 | W = 39772 | .015 | 0.10 |
|  | ASD_STAI | 0 – 12 | 4.92 | 3.33 |  |  |  |
| Disorganized schizotypy (MSS-B) | mNTP_STAI | 0 – 12 | 3.66 | 3.57 | W = 44599 | < .001 | 0.20 |
|  | ASD_STAI | 0 – 12 | 5.34 | 3.79 |  |  |  |
| Perceived social support (MSPSS) | mNTP_STAI | 12 – 60 | 39.50 | 11.75 | W = 29102 | < .001 | 0.13 |
|  | ASD_STAI | 12 – 60 | 35.88 | 11.35 |  |  |  |
| Psychological inflexibility (AAQ-2) | mNTP_STAI | 7 – 49 | 26.89 | 10.63 | W = 44260 | < .001 | 0.19 |
|  | ASD_STAI | 7 – 49 | 31.72 | 10.12 |  |  |  |

*Notes.* mNTP_STAI = matched neurotypical controls without psychiatric diagnosis after matching on STAI-T scores, ASD_STAI = autism spectrum disorder in the STAI-T based matching

### Figure S20. Visual representation of the partial correlation network structure of the mNTP_STAI group

**
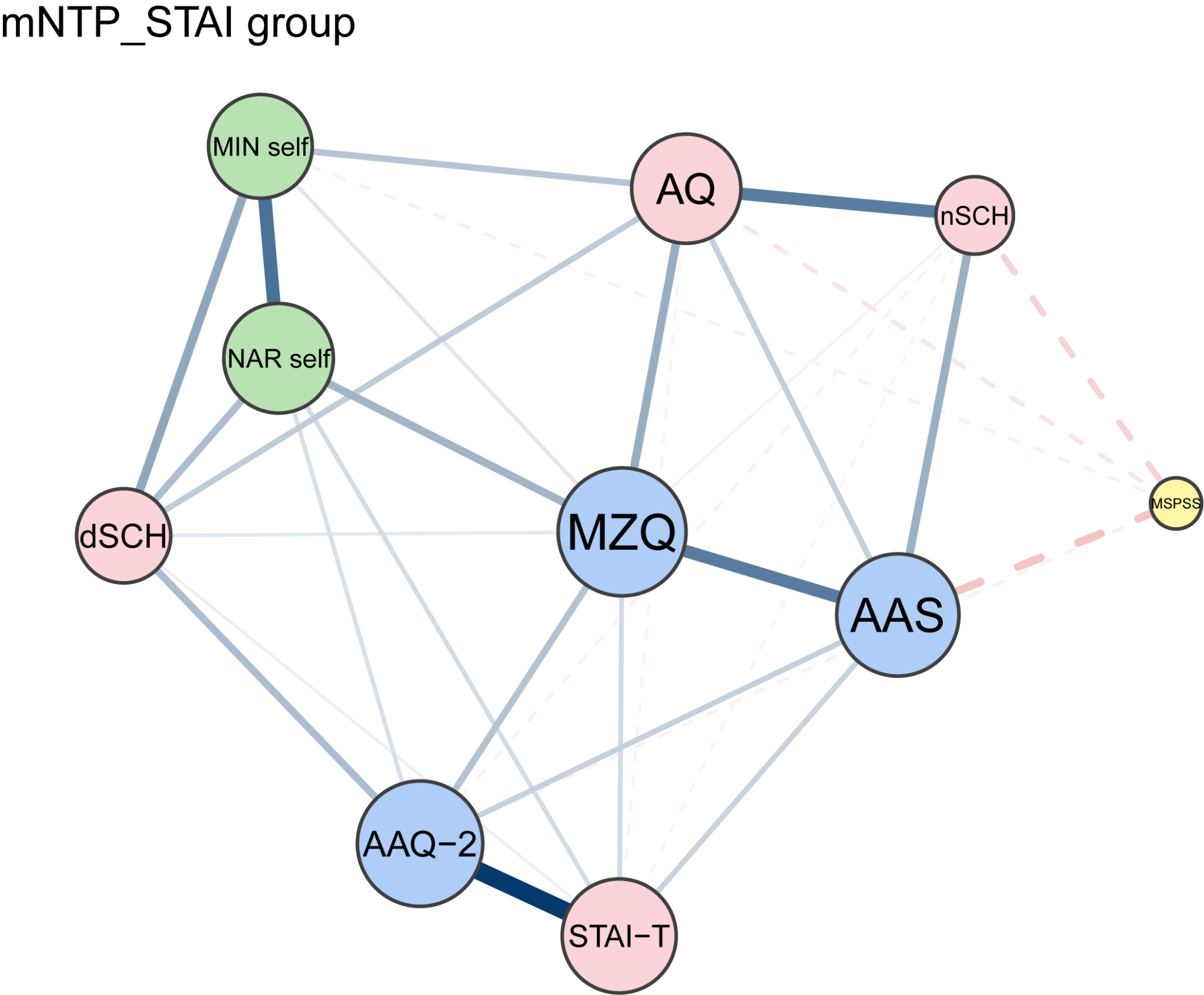
**

*Notes*. Each node represents a psychological construct, and edges represent regularized partial correlations between them. Blue edges indicate positive associations, while red edges indicate negative associations. The thickness of the edge corresponds to the magnitude of the partial correlation. Node size reflects the predictability of each variable. Node colors were assigned to visually distinguish conceptual domains within the network: psychopathology-related traits (pink), dimensional psychological factors (blue), self-related constructs (green), and perceived social support (yellow). The colors are intended for interpretive clarity and do not reflect statistical clustering. mNTP_STAI_group = matched neurotypical controls without psychiatric diagnosis after matching on STAI-T scores, AQ = Autism Spectrum Quotient, dSCH = disorganized schizotypy, nSCH = negative schizotypy, STAI-T = trait anxiety, AAQ-2 = psychological inflexibility, AAS = insecure attachment, MZQ = mentalization, MIN self = minimal self, NAR self = narrative self, MSPSS = perceived social support.

### Figure S21. Node centralities of the estimated STAI matched network models


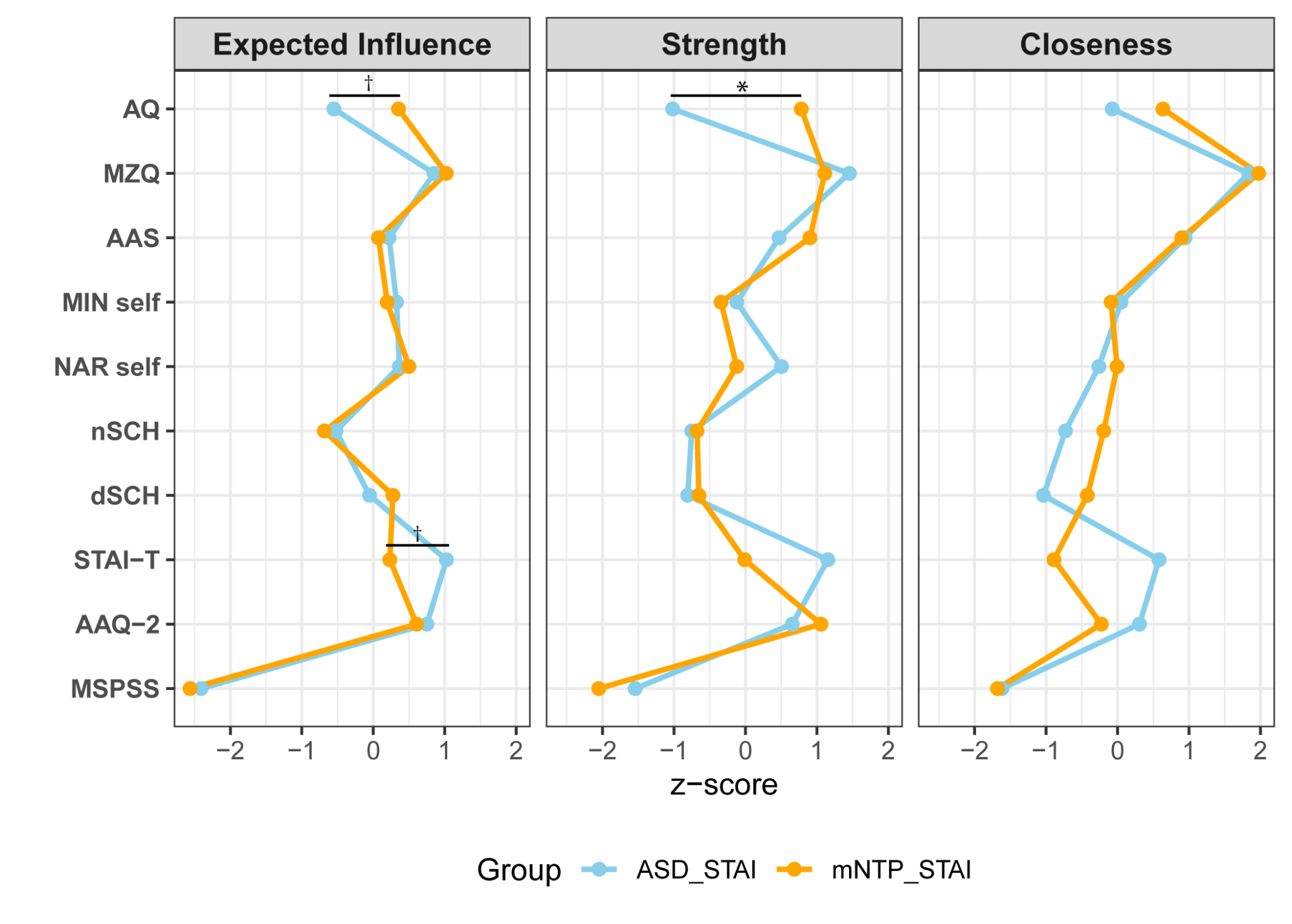


*Notes*: ASD_STAI = autism spectrum disorder in the STAI-T based matching, mNTP_STAI = matched neurotypical controls without psychiatric diagnosis matched based on STAI-T scores, AQ = Autism Spectrum Quotient, dSCH = disorganized schizotypy, nSCH = negative schizotypy, STAI-T = trait anxiety, AAQ-2 = psychological inflexibility, AAS = insecure attachment, MZQ = mentalization, MIN self = minimal self, NAR self = narrative self. Statistical significance is indicated by * (p < 0.05), and trend-level effects by † (p < 0.1).

### Table S11. Node-level network invariance

|  | **Strength** | | **Expected influence** | | Closeness | |
| --- | --- | --- | --- | --- | --- | --- |
| **Node** | **difference** | **p-value** | **difference** | **p-value** | difference | p-value |
| AQ_sum | -0.517 | **0.021** | -0.442 | *0.065* | -0.003 | 0.292 |
| mzq_sum | 0.017 | 0.898 | -0.132 | 0.536 | -0.002 | 0.514 |
| aas_insecure | -0.180 | 0.452 | 0.029 | 0.898 | -0.002 | 0.514 |
| esss_minimal_self | -0.080 | 0.582 | 0.018 | 0.898 | -0.002 | 0.514 |
| esss_narrative | -0.029 | 0.874 | -0.105 | 0.514 | -0.002 | 0.442 |
| mss_neg_sum | -0.166 | 0.582 | 0.051 | 0.874 | -0.003 | 0.292 |
| mss_dis_sum | -0.185 | 0.322 | -0.185 | 0.442 | -0.003 | 0.292 |
| stai_t_sum | 0.164 | 0.514 | 0.309 | *0.074* | 0.001 | 0.691 |
| aaq_sum | -0.166 | 0.514 | 0.016 | 0.898 | -0.001 | 0.740 |
| mspss_adol_sum | -0.088 | 0.582 | 0.088 | 0.582 | -0.002 | 0.452 |

*Note*. Node-level network invariance in ASD_STAI vs. mNTP_STAI group comparison
